# An efficacy analysis of whole-body magnetic resonance imaging in the diagnosis and follow-up of polymyositis and dermatomyositis

**DOI:** 10.1371/journal.pone.0181069

**Published:** 2017-07-17

**Authors:** Zhen-guo Huang, Bao-xiang Gao, He Chen, Min-xing Yang, Xiao-liang Chen, Ran Yan, Xin Lu, Kai-ning Shi, Queenie Chan, Guo-chun Wang

**Affiliations:** 1 Department of Radiology, China-Japan Friendship Hospital, Beijing, China; 2 Department of Rheumatology, China-Japan Friendship Hospital, Beijing, China; 3 Philips Healthcare, Beijing, China; RIKEN Advanced Science Institute, JAPAN

## Abstract

**Objectives:**

To evaluate the value of whole-body magnetic resonance imaging (WBMRI) in diagnosing muscular and extra muscular lesions in patients with polymyositis (PM) and dermatomyositis (DM).

**Methods:**

A retrospective analysis of WBMRI data from PM/DM patients who met the Bohan and Peter diagnostic criteria was performed. X^2^ test was used to compare the rate of positive diagnosis of newly diagnosed patients using WBMRI, serum creatine kinase test, and EMG. McNemar test was used to compare the performance of WBMRI and chest CT in detecting interstitial lung disease (ILD).

**Results:**

The study included 129 patients (30 PM cases and 99 DM cases). Of them, 81.4% (105/129) showed a visible inflammatory muscular edema on their WBMRI; 29.5% (38/129) had varying degrees of fatty infiltration (9 cases with clear muscular atrophy). Of the 66 newly diagnosed patients, the positive rates of WBMRI, muscle biopsy, serum creatine kinase test and EMG were 86.4% (57/66), 92.4% (61/66), 71.2% (47/66) and 71.1% (32/45), respectively. There was no significant difference in the positive rates between WBMRI and muscle biopsy (X^2^ = 1.28, P = 0.258). The WBMRI had a higher positive rate than both serum creatine kinase test (X^2^ = 4.53, P = 0.033) and EMG (X^2^ = 3.92, P = 0.047). In addition to muscular changes, WBMRI also detected interstitial lung disease (ILD) in 38 cases (29.5%), osteonecrosis in 15 cases (11.6%), and neoplastic lesions (5 malignant; 7 benign) in 12 cases (9.3%). Of the 61 patients who underwent routine chest CT examinations, the WBMRI and CT revealed ILD in 29 cases and 35 cases respectively. There was no significant difference in the sensitivity between WBMRI and CT (p = 0.146).

**Conclusions:**

WBMRI is a sensitive, non-invasive and efficient imaging method. It comprehensively displays the extent of muscular involvement in PM/DM patients, and it has the ability to diagnose other associated extra muscular diseases, such as ILD and systemic malignancy. WBMRI can also help screen steroid-induced osteonecrosis.

## Introduction

Polymyositis (PM) and dermatomyositis (DM) fall into the class of idiopathic inflammatory myopathies, a group of autoimmune diseases characterized by inflammatory changes of the skeletal muscle [1]. Magnetic resonance imaging (MRI) is a noninvasive examination which has no ionizing radiation and can scan a large range of the body. Although MRI findings may not be specific, it can clearly show muscular and subcutaneous inflammatory lesions, evaluate its extent, identify potential sites for biopsy, assess disease burden, and help monitor the progression or regression of disease [2–6]. Conventional MRI usually includes limited scanning of the proximal lower limb muscle girdle or scanning of the proximal upper limb girdle. In recent years, there have been reports that demonstrate the success of whole-body magnetic resonance imaging (WBMRI) through short tau inversion recovery (STIR) sequence in the diagnosis of PM / DM [7–9]. WBMRI has the advantage of documenting inflammatory myopathy of the whole body, including the psoas, intercostal, and neck muscles, which can not be done by conventional MRI.

Although muscular and skin changes are characteristic presentations, PM / DM is a systemic disease. Lungs are the second most involved organ after the skin and muscular system. Interstitial lung disease (ILD) is the most frequent manifestation, reported in up to 35–40% of DM patients [10]. Meanwhile, a considerable proportion of PM / DM patients were also report malignance. ILD and cancer are important factors affecting the prognosis of PM / DM patients [10]. Furthermore, glucocorticoids are preferred treatments for PM / DM patients, but are also the primary cause of non-traumatic osteonecrosis [11]. WBMRI scan covers the whole body. It can comprehensively display the whole body muscular involvement in patients with PM / DM. However, its value in the diagnosis of ILD, cancer and other PM / DM associated extra muscular lesions remains unclear.

This study includes a retrospective analysis of 129 PM / DM patients and of their clinical and WBMRI data. The objective is to evaluate the value of WBMRI in displaying PM / DM related muscular changes and extra muscular lesions (ILD, systemic malignancies), as well as the occurrence of osteonecrosis after glucocorticoids therapy. To the best of our knowledge, there has been no report assessing the value of WBMRI in diagnosing extra muscular manifestations (ILD, systemic malignancies) in PM / DM cases. So far, the use of WBMRI in the diagnosis of osteonecrosis in PM / DM patients has been limited only to the case report [12], and we are not aware of any report documenting WBMRI for the diagnosis of extra muscular manifestations (ILD, systemic malignancies).

## Methods and materials

### Study population

Inclusion criteria: (1) Met the Bohan and Peter diagnostic criteria of PM / DM, including ① symmetrical myasthenia; ② characteristic pathological changes in muscle biopsy; ③ elevation of serum muscle enzymes; ④ myogenic damage on electromyogram (EMG); and ⑤ characteristic dermatological lesions. The patient was diagnosed as PM if three out of the four criteria were met, and was diagnosed as DM if dermatological lesions were also present [13]. (2) Received muscle biopsy. (3) Underwent WBMRI examination. (4) Willing to participate in the study. The study was approved by the ethics committee of China-Japan friendship hospital, and the individual in this manuscript has given written informed consent to publish these case details.

### MRI examination

All patients underwent whole-body coronal and thigh axial MRI scan using a Philips-Ingenia 3.0T MRI machine (Philips Medical Systems, Best, the Netherlands), which employed orthogonal body coil and automatic moving-bed technology. The WBMRI coronal scan used a STIR sequence, and the scan parameters were: TR / TE = 3996 / 70ms, TI = 230ms, ETL = 53, 20 layers; layer thickness 7mm; interlayer spacing 0.7mm; FOV = 512 × 320mm; matrix: 480 × 480. The coronal scan from head to toe was performed in six consecutive segments, and the breath holding technique was applied for chest and abdomen scans. The scanning time for each segment was 17.8 seconds. The coronal WBMRI using STIR sequence took a total of 10–12 minutes, which included the time to position the image, move the bed, shimming, etc. After the completion of the coronal scan, built-in MobiView software was used to integrate 6 segmental images into coronal STIR-WBMRI images. The bilateral axial scan of the thigh area was done by using a T1-weighted (T1W) and T2-weighted (T2W) / spectrally selective attenuated inversion recovery (SPAIR) sequence. Scan parameters were: T1W: TR / TE = 566 / 10ms, layer thickness 5mm, interlayer spacing 1mm, FOV = 380 × 192mm, matrix 380 × 179, scanning time 25 seconds; T2W / SPAIR: TR / TE = 3464 / 70ms, layer thickness 5mm, interlayer spacing 1mm, FOV = 380 × 192mm, matrix: 476 × 229, scanning time one minute and 25 seconds. The examination took about 15 minutes.

### Image analysis

Inflammatory muscular edema was defined as increased muscle signal on the STIR images; the degree of the increased signal indicating the severity of the edema. Muscle adipose infiltration was defined as T1W high signal caused by intramuscular abnormal fat deposition. Muscle atrophy was defined as the reduction of muscle volume. ILD was defined as the presence of one or more of the following changes on lung imaging: reticular nodule, ground-glass or patchy opacities, consolidation, irregular linear opacities, honeycombing or stretching, and dilatation of the bronchi. Osteonecrosis was defined as map-like lesions surrounded by a curving high signal line in epiphyses or metaphyses on the STIR images. Two experienced and study-blind radiologists independently reviewed all of the images. Muscular and extra muscular manifestations on the WBMRI and changes on the chest CT were recorded. A third radiologist with more than 20 years of experience adjudicated disagreements in musculoskeletal imaging diagnoses. Chest CT evaluation was conducted one week after completing WBMRI imaging analysis to avoid influence from the WBMRI on the ILD diagnosis.

### Statistical analysis

Disease duration was defined as the interval from the onset of symptoms to the time of WBMRI exam. Positive PM / DM cases were defined as those with characteristic pathological changes in muscle biopsy, serum creatine kinase ≥ 200 U / L, myogenic damage visible on the EMG, or inflammatory intramuscular edema on the WBMRI. SPSS17.0 was used for statistical analysis. X^2^ test was used to compare the rate of positive diagnosis of newly diagnosed patients using WBMRI, muscle biopsy, serum creatine kinase test, and EMG. McNemar test was used to compare the performance of WBMRI and chest CT in detecting interstitial lung disease (ILD). The sensitivity, specificity, diagnostic accuracy, and the positive and negative predictive value of WBMRI were also calculated. The results of the WBMRI for detecting ILD were measured against the results of the Chest CT scan. Independent samples t test was used to analyze the effect of disease duration to muscle adipose infiltration. A Kappa test was used to evaluate the agreement between two observers on the evaluation of muscular and extra muscular changes on WBMRI.

## Results

### Patient characteristics

In our hospital, patients that were suspected PM / DM and had not contraindications of MRI examination routine underwent WBMRI. From August 2013 to April 2015, WBMRI was undergone in 165 patients. Of them, 129 patients met the inclusion criteria and were enrolled in this study. There were a total of 30 PM cases and 99 DM cases. Of the PM patients, 11 were males and 19 were females, with ages ranging from 12 to 62 years, and a mean age of 38.3 (± 15.3) years. Of the DM patients, 31 were male, 68 were female, with ages ranging from 10 to 86 years, and a mean age of 50.7 (± 15.4) years. 66 cases were newly diagnosed patients. 63 cases were previously diagnosed as PM / DM and underwent WBMRI examination due to suspicion of relapse. The clinical and examination results of the 129 patients were shown in Table 1. The disease duration ranged from 10 days to 19 years with a mean time-span of 30.8 (± 47.9) months. 18 cases received two or more WBMRI exams. 13 of the 66 newly diagnosed patients and all 63 previously diagnosed patients had a history of corticosteroid use before the first WBMRI examination. In addition, 61 patients took chest CT scan within one week of receiving their WBMRI exam. Of the 66 newly diagnosed patients, serum muscle enzyme tests were performed within the three days prior to the MRI examination.

**Table 1:** The clinical and examination results of the 129 patients with PM / DM.

	Positive cases (newly diagnosed / reviously diagnosed cases)	Negative cases (newly diagnosed / reviously diagnosed cases)	Total cases (newly diagnosed / reviously diagnosed cases)
erythra	62/59	4/4	66/63
dermatological lesions	52/47	14/16	66/63
Muscle biopsy	61/57	5/6	66/63
serum muscle enzyme	47/47	19/16	66/63
EMG	32/33	13/16	45/49

### Intramuscular change

**Fig 1:**
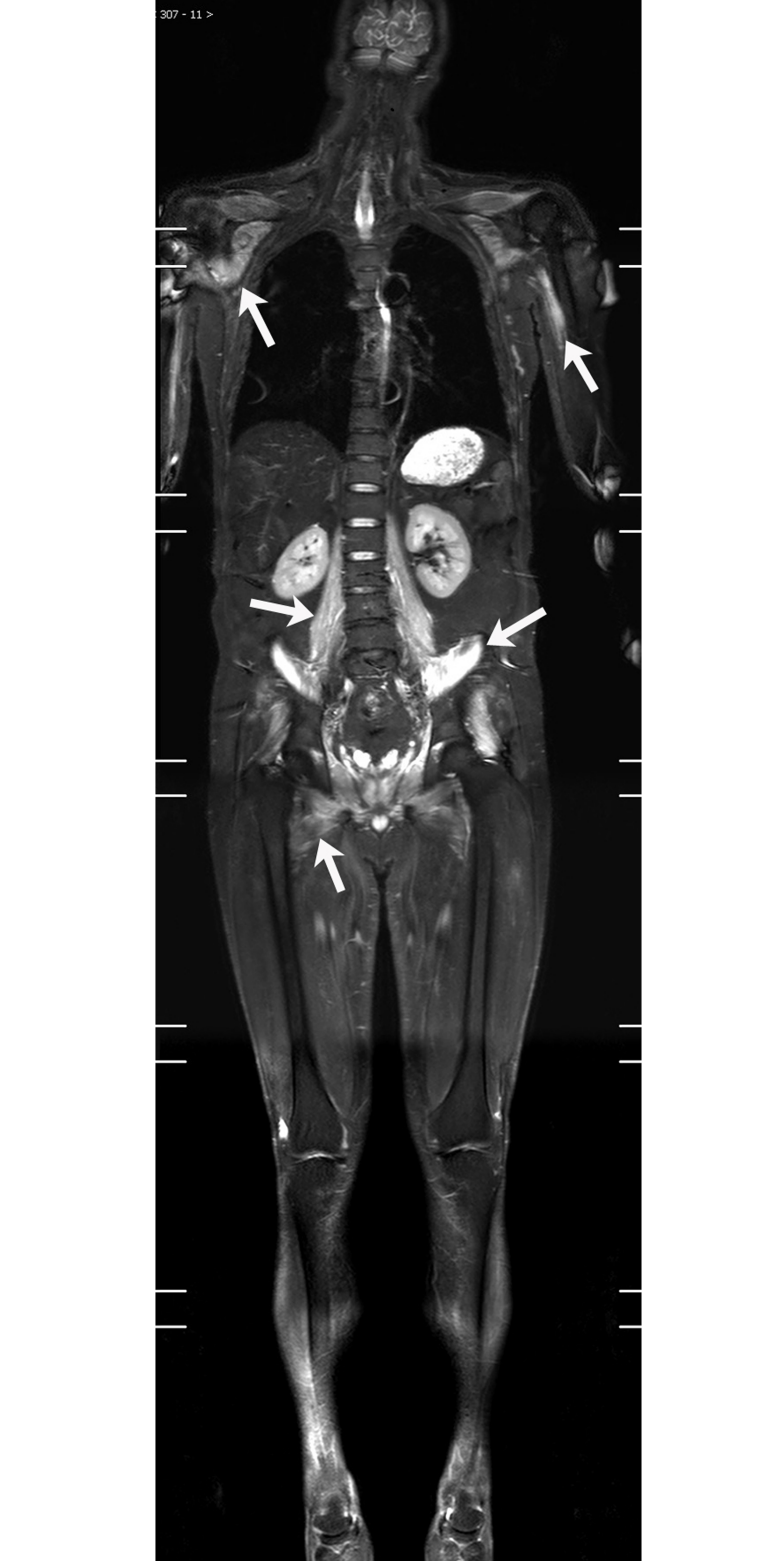
49-year-old male DM patient. WBMRI showed significantly increased signal intensity (white arrow) in both shoulders, upper extremities, pelvic muscles and lumbar muscles. The severity of muscle edema in both thighs was significantly lower than that in the above-mentioned sites.

There was a clear inflammatory muscular edema on the WBMRI in 105 patients (81.4%), including 57 (86.4%) newly diagnosed patients and 48 (76.2%) follow-up patients. The location and distribution characteristics of the inflammatory edema were shown in Table 2. Among the 105 patients with inflammatory muscular edema, the most frequently affected area was the thigh muscle, presenting in 99.0% (104/105) cases. The degree of edema was consistent throughout the whole body in 21.9% (23/105) of cases, and inconsistent in 78.1% (82/105) of cases. Among the 82 cases with various degrees of muscular edema, the body parts that had the most serious inflammatory edema were the thigh (n = 45), shoulder (n = 14), leg (n = 8), waist (n = 7), pelvis (n = 5) and upper limb (n = 3). In 35.2% (37/105) of patients, the severity of muscle edema in both thighs was significantly lower than that in other parts of the body (Fig 1). Among the 66 newly diagnosed patients, nine patients had mild fatty infiltration of the thigh muscles (no muscular atrophy), while in the 63 follow-up patients, 29 had varying degrees of fatty infiltration in the thigh muscles (nine cases with obvious muscular atrophy). The disease duration of patients with muscular fatty infiltration (ranging from 4 to 229 monthes, mean 62.96 ±65.39 monthes) was significantly higher than that of patients without muscular fatty infiltration (ranging from 1 to 196 monthes, mean 20.46 ±36.28 monthes), (t = -3.737, P<0.01). Of the 18 cases that underwent two or more WBMRI, 10 cases showed significant improvement of inflammatory muscular edema through the follow-up WBMRI (post treatment), 5 cases showed no significant changes, and 3 cases showed further progression.

**Table 2:** Characteristics of 105 patients presented with inflammatory lesions on WBMRI.

	Involved body parts						Subcutaneous inflammation	Lesion distribution	severity
	Lower extremity	Pelvis	Lumbar muscle	Chest	Upper extremity	neck	Yes/No	Symmetrical / unsymmetrical	Symmetrica [/ unsymmetrical
DM	80	73	67	62	72	64	**52/29**	71/10	14/67
PM	24	21	18	18	21	14	5/19	21/3	3/21
Total	104	94	85	80	93	78	57/48	92/13	17/88

### ILD, osteonecrosis and malignant lesions (Table 3)

**Table 3:** Extramuscular changes on STIR-WBMRI in 129 patients.

	ILD (Y/N)	Malignancy (Y/N)	Osteonecrosis (Y/N)
PM	5/25	0/30	3/27
DM	33/66	5/94	12/87
Total	38/91	5/125	15/114

**Fig 2:**
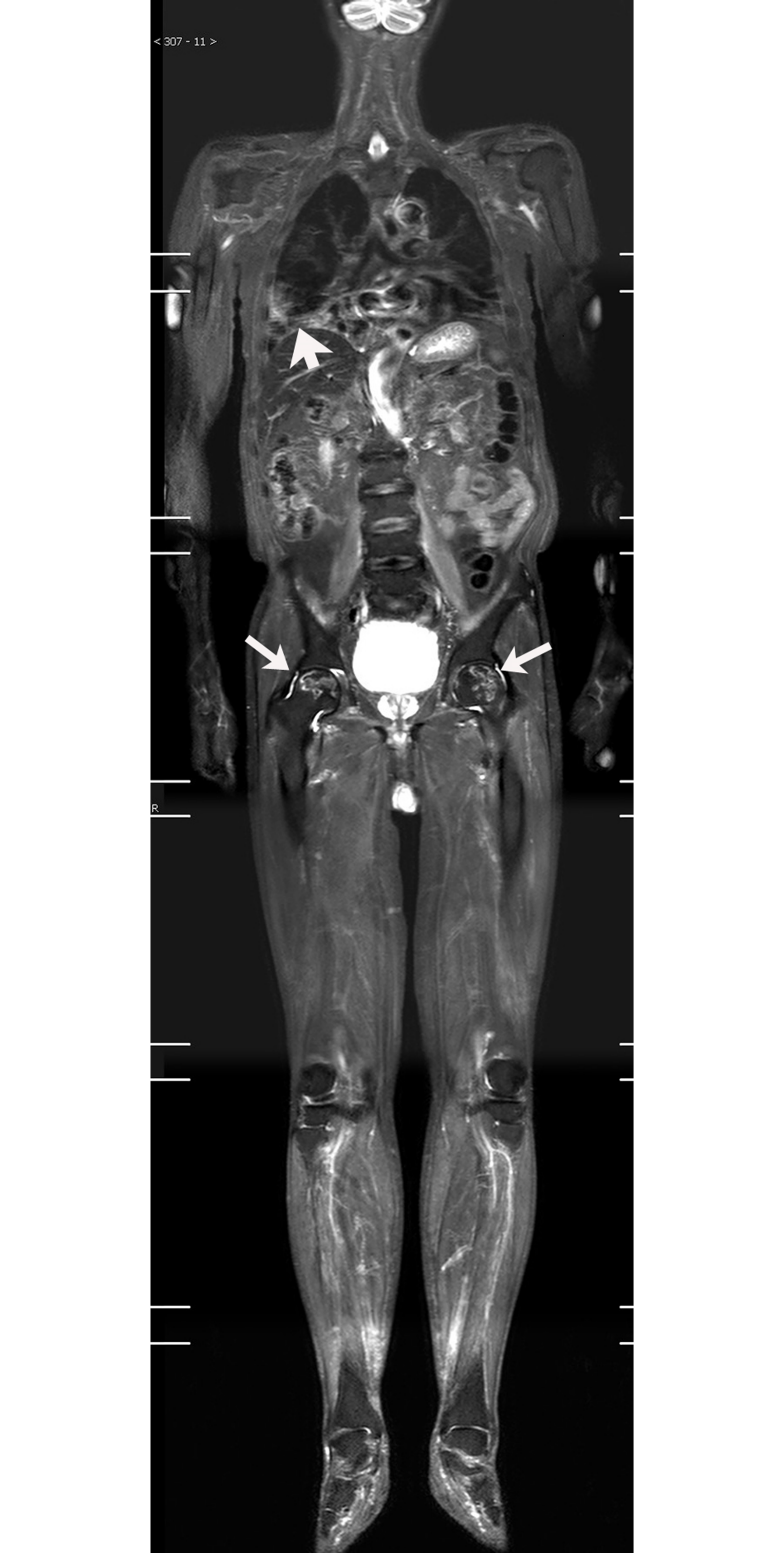
75-year-old male patient, displaying DM with bilateral lung ILD, bilateral femoral head necrosis. Follow-up WBMRI showed patchy, reticulonodular and ground glass opacities in bilateral lungs; the most pronounced in the lower right lobe (white arrow). Both femoral heads showed the osteonecrosis area (white arrow) surrounded by curving high signal.

**Fig 3:**
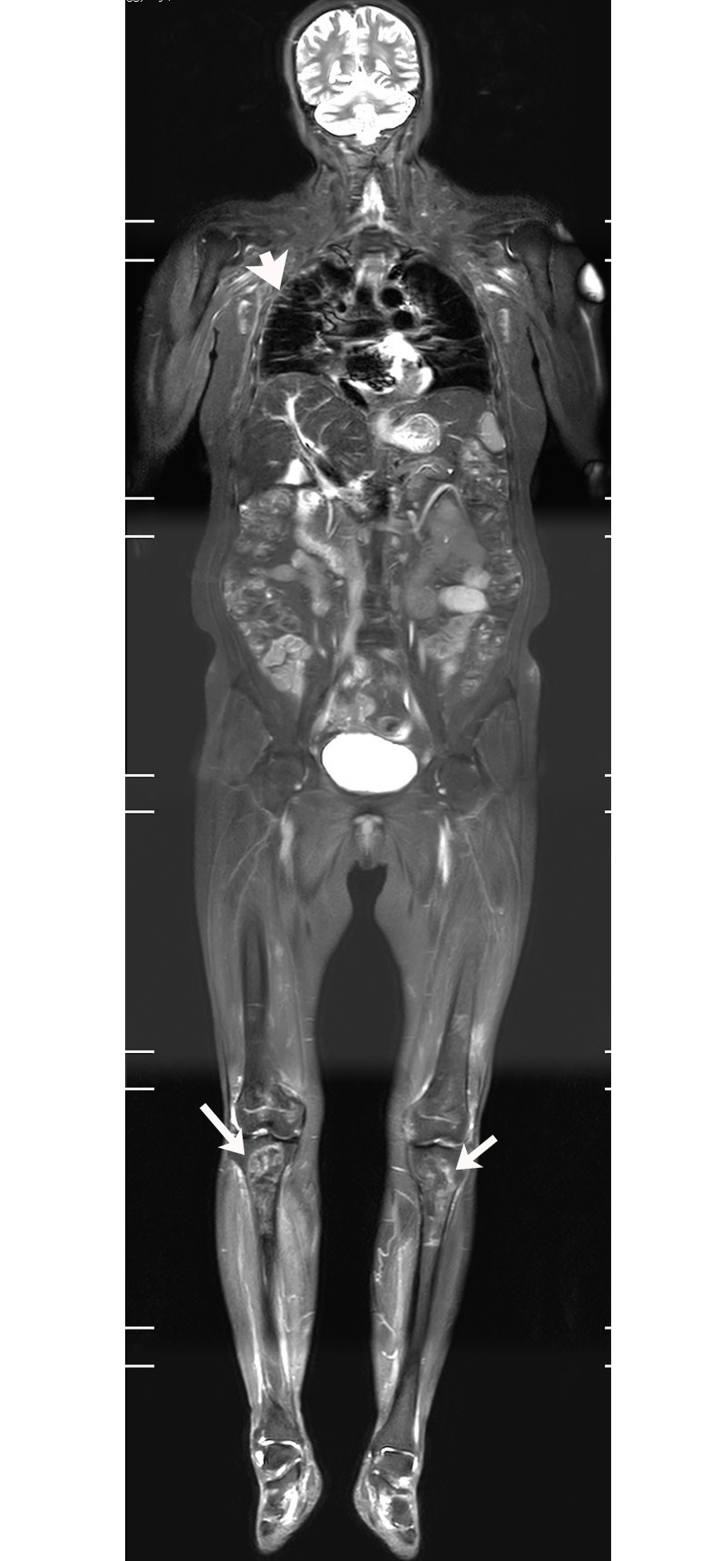
61-year-old female patient, displaying DM with lung ILD, osteonecrosis in both knees. WBMRI showed bilateral tibia osteonecrosis (white arrow) and fibrous streaks (white arrow) in the outer lung field, suggesting the presence of ILD.

The WBMRI showed ILD in 29.5% (38/129) of patients (Figs 2 and 3). Of the 61 patients who underwent routine chest CT examinations, the comparison between their chest CT and WBMRI in terms of sensitivity in diagnosing ILD is summarized in Table 4. There was no significant difference in the sensitivity between WBMRI and CT (P = 0.146). Using chest CT as the standard for diagnosis of ILD, the sensitivity, specificity, diagnostic accuracy, and the positive and negative predictive value of WBMRI were 74.3%, 88.5%, 80.3%, 89.7% and 71.9%, respectively.

**Table 4:** Comparison of WBMRI and CT on ILD diagnosis in 61patients.

WBMRI	CT		Total
	+	−	
+	26	3	29
−	9	23	32
Total	35	26	61

**Fig 4:**
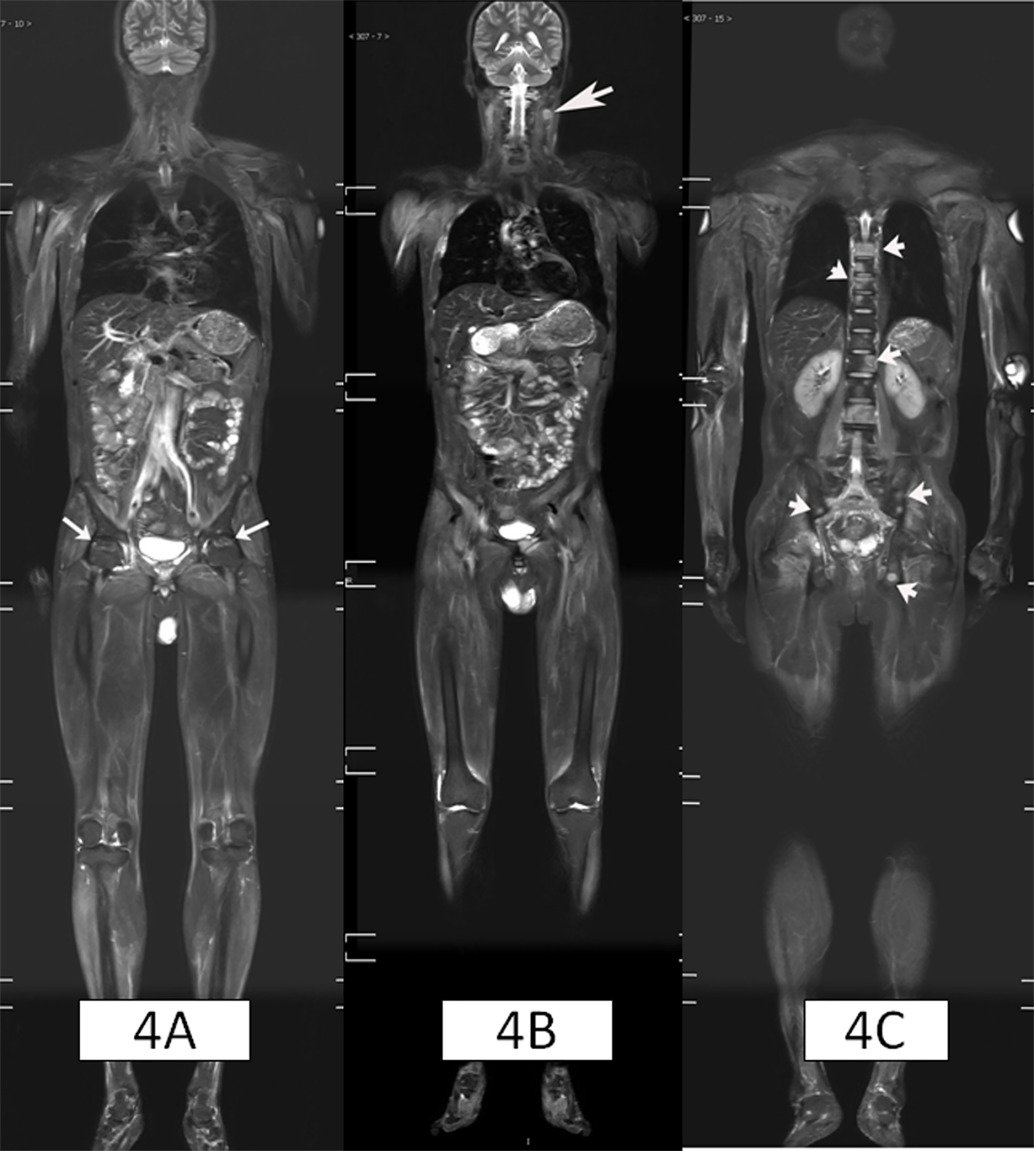
43-year-old male patient, displaying DM with nasopharyngeal cancer and cervical lymph nodes metastases, multiple bone metastases and bilateral femoral head necrosis. Figure 4A showed the avascular necrosis at bilateral femoral head (white arrows); and Figure 4B showed swollen lymph node on the left side of the neck (white arrow); Figure 4C showed patchy abnormal high signals in the thoracic and lumbar spine and pelvis (white arrow). Vertebral biopsy confirmed skeletal metastases.

**Fig 5:**
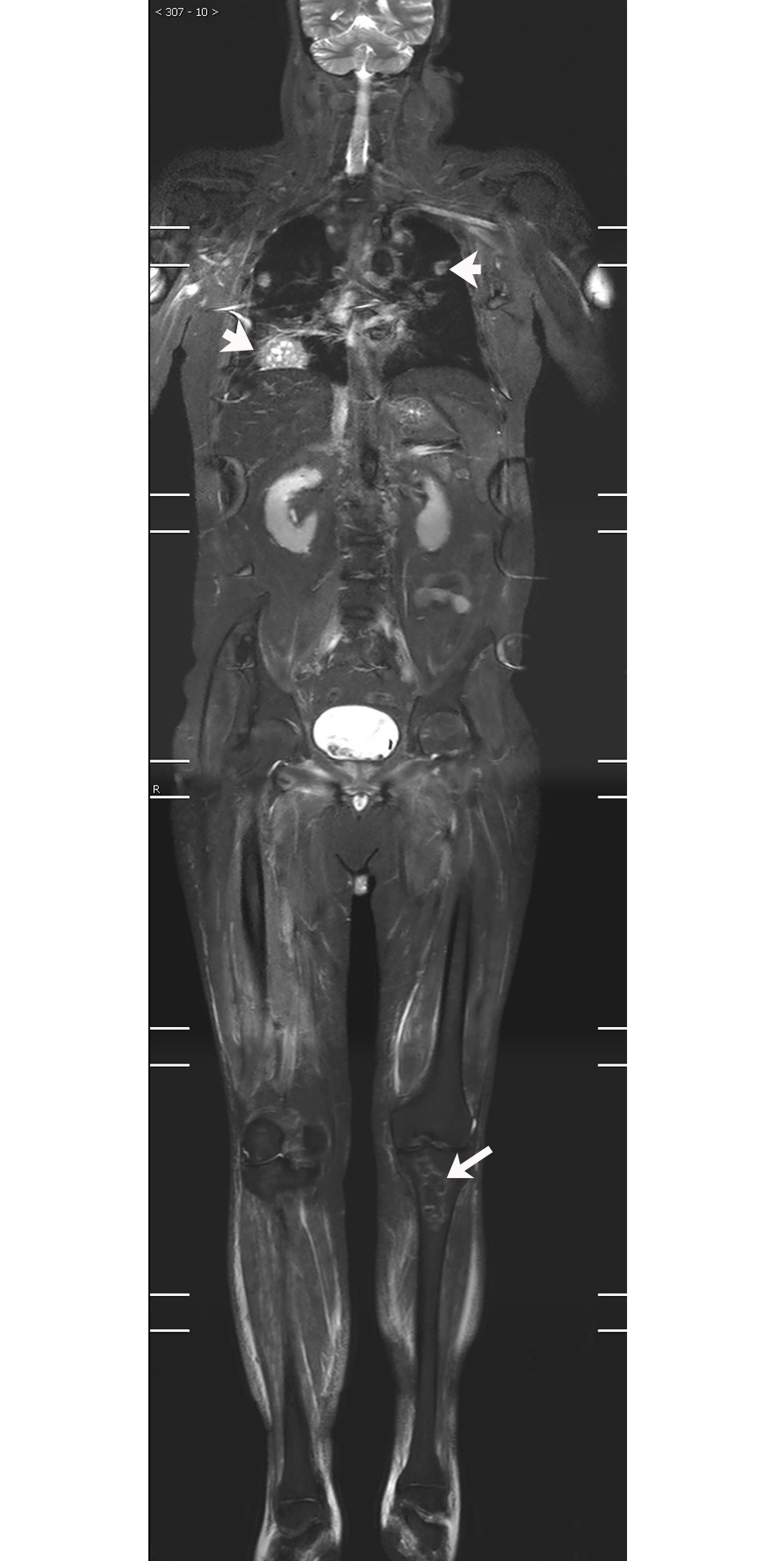
35-year-old male DM patient, with lung fungal infection and bilateral osteonecrosis of the knee after steroid therapy. WBMRI showed multiple spherical lesions in bilateral lungs (white arrow), and the osteonecrosis area (white arrow) surrounded by curvy high signal in the upper left tibia.

WBMRI detected osteonecrosis in 15 patients. All patients with osteonecrosis had a history of steroid use before the WBMRI examination. Until the diagnosis on WBMRI, the total dose of steroid ranged from 3100 to 18950 mg prednisolone or its equivalent (mean 6354 mg) and the duration of steroid treatment ranged from 1 month to 84 months (mean 23.5 months). Thirty-eight joints were affected (mean, 2.5 joints per patient; range, 1–5 joints). The hip was the most often affected body part (19 hips in 11 patients) (Figs 2 and 4A), followed by knee (13 knees in 7 patients) (Figs 3 and 5), shoulder (3 shoulders in 2 patients), and ankle (3 ankles in 2 patients). Of the 38 joints affected by osteonecrosis, 33 had no clinical symptoms. No articular surface collapse occurred except in one hip with femoral head osteonecrosis. A regional MRI was also performed in five patients with osteonecrosis. The WBMRI detected all 10 osteonecrotic sites seen on the regional MRI. The location, shape, and size of the osteonecrotic lesions revealed on the regional MRI were in accordance with those displayed on the WBMRI. Among the 18 cases that underwent two or more WBMRI, the first STIR-WBMRI scan revealed that there was no osteonecrosis in any skeleton and the second STIR-WBMRI scan detected osteonecrosis in 2 patients. Of the 15 patients with osteonecrosis, routine radiography of the affected joints was performed in six of them, and it did not reveal osteonecrotic lesions.

**Fig 6:**
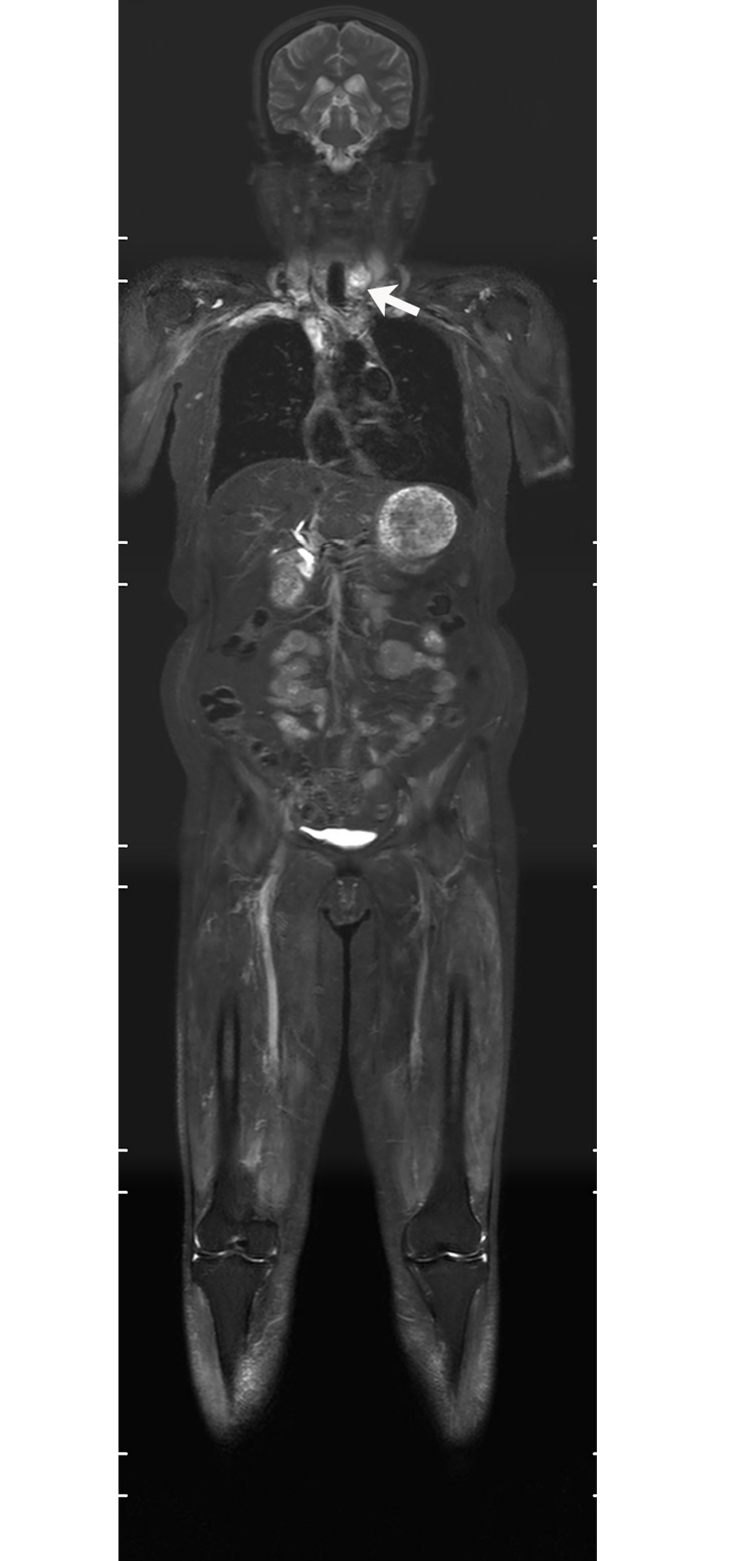
60-year-old woman, displaying DM with thyroid cancer. WBMRI showed enlargement of the left thyroid, in which there were oval-shaped, abnormally high signals (white arrow) that was diagnosed as thyroid cancer by biopsy.

The WBMRI discovered tumors in 12 patients (9.3%). Five were later diagnosed as malignant, and seven were diagnosed as benign tumors. Cervical lymphadenopathy was identified in two cases. Subsequent nasopharyngoscopy revealed a nasopharyngeal tumor, and patients were diagnosed as nasopharyngeal carcinoma after biopsy. The final clinical diagnosis was cervical lymph node metastasis of nasopharyngeal carcinoma. In another case, WBMRI showed swollen cervical lymph nodes (Fig 4B) and multiple abnormal signals in the thoracic and lumbar vertebrae, as well as the pelvis (Fig 4C). Bone biopsy of the lumbar lesion showed bone metastasis of the nasopharyngeal carcinoma. Subsequent nasopharyngoscopy revealed a nasopharyngeal tumor. The final clinical diagnosis was cervical lymph node and multiple bone metastasis of nasopharyngeal carcinoma. One case had ovarian cancer before the diagnosis of DM and had already undergone a surgical resection. The WBMRI detected solitary liver nodules combined with ascites. The patient was later diagnosed as liver metastasis by PET-CT scan. WBMRI identified thyroid nodules in four cases, which were later biopsied and diagnosed as thyroid cancer in 1 case (Fig 6), thyroid adenoma in 3 cases. In addition, the WBMRI also showed two cases of adrenal adenoma (confirmed in combination with CT scan and blood tests), two cases of hepatic hemangioma (confirmed in combination with enhanced CT scan).

### Other changes on WBMRI

The WBMRI also showed four cases of pleural effusion, three cases of ascites, four cases of pericardial effusion, two cases of gallbladder stones, six cases of uterine fibroids, and one case with multiple spherical lung cavity lesions (later confirmed to be a fungal infection) (Fig 5).

### Comparison of positive diagnosis rates between the WBMRI, muscle biopsy, serum creatine kinase enzyme test, and EMG

Of 66 newly diagnosed patients, the positive rates for WBMRI, muscle biopsy, serum creatine kinase test and EMG were 86.4% (57/66), 92.4% (61/66), 71.2% (47/66) and 71.1% (32/45), respectively. There was no significant difference in the positive rates between WBMRI and muscle biopsy (X^2^ = 1.28, P = 0.258). The WBMRI had a higher positive rate than both serum creatine kinase test (X^2^ = 4.53, P = 0.033) and EMG (X^2^ = 3.92, P = 0.047).

### Comparison of WBMRI findings between patients with DM and PM

The rate of muscle involvement, the location and distribution of muscle inflammatory edema were similar in patient with DM and PM (Table 2). However, in patients with muscle involvement, DM patients had higher subcutaneous tissue involvement rate than PM patients (64.2% versus 20.8%, X^2^ = 14.03, P < 0.01) (Table 2). The prevalence of ILD in DM patients (33.3%) was higher than that in PM patients (16.7%), but the difference was no statistically significant (X^2^ = 3.078, p = 0.079) (Table 3). The rate of osteonecrosis was similar in patient with DM and PM (12.1% versus 10%, X^2^ = 0.10, P = 0.75) (Table 3). Among 129 patients, 5 patients with malignant tumor were all DM patients.

### Comparison of thigh changes with whole body changes on WBMRI

There was a clear inflammatory muscular edema on the WBMRI in 105 patients. Although 99.0% (104/105) cases had definite thigh muscle involvement, the severity of muscle edema in both thighs was significantly lower than that in other parts of the body in 35.2% (37/105) cases (Fig 1). In addition to muscular changes, WBMRI also detected interstitial lung disease (ILD) in 38 cases (29.5%), osteonecrosis in 15 cases (11.6%), and neoplastic lesions (5 malignant; 7 benign) in 12 cases (9.3%).

### The agreement between two observers on the assessment of muscular and extra muscular changes using WBMRI

Two radiologists had good consistency in determining whether there are muscle inflammatory lesions, ILD, osteonecrosis, or a tumor using a WBMRI. Kappa values were 0.773, 0.816, 0.963 and 0.792, respectively.

## Discussion

Serum muscle enzymes test, EMG and muscle biopsy are the main conventional diagnostic methods for PM / DM. All three tests have limitations. First, the muscle biopsy and EMG are invasive and not ideal for repetitive testing. Secondly, both the muscle biopsy and the EMG can only display the muscular condition of a particular site. When inflammation is unevenly distributed, biopsy or EMG conducted at a site without obvious inflammation may lead to a false negative or undetermined diagnosis. More than 10% of muscle biopsies conducted in PM / DM patients were false negatives [14]. Serum muscle enzymes tests are easy, convenient and repeatable, but not comprehensive. Some PM / DM patients have normal or only slightly elevated serum muscle enzyme levels, and various causes other than PM / DM can also lead to increased serum muscle enzyme levels. This study showed that detection rates of muscular changes using a WBMRI was higher than the serum creatine enzyme test and the EMG examination. This suggests that WBMRI has higher sensitivity in diagnosing muscle lesions in patients with PM / DM. This study also found that although thigh musculature was the most frequently involved part in PM / DM patients, the severity of muscle edema in both thighs was significantly lower than that in other parts of the body in about 1/3 of patients, indicating that muscular inflammation in PM / DM patients is often unevenly distributed, and that relying on MRI findings of thigh muscles alone may lead to misjudgment.

WBMRI examination takes about 15 minutes, similar to the time length of a conventional single-site MRI. However, WBMRI can reveal muscular involvement throughout the whole body, evaluate the severity of the disease more objectively and accurately, assess the burden of disease more effectively, and determine the optimal biopsy site. Our study found that in addition to displaying muscular changes, a WBMRI also identified ILD in 38 cases, tumor lesions in 12 patients (five malignant and seven benign), and osteonecrosis in 15 cases.

The lungs are the second most involved organ, besides the muscular system, in PM / DM. ILD is the main manifestation of PM / DM in the lung, an important prognostic factor [15–17]. The assessment of WBMRI in the detection of ILD has not been reported before. In our study, 61 patients underwent chest CT examinations. The sensitivity of WBMRI in identifying ILD was similar to the sensitivity of CT scan. Although the chest CT scan is the best imaging modality for diagnosing ILD, it emits ionizing radiation. In contrast, WBMRI is harmless and is also capable of revealing the existence and severity of ILD, in addition to assessing the muscular inflammatory changes associated with PM / DM. Most importantly, WBMRI does not increase a patient’s health care burden.

Although the correlation mechanism remains unclear, it is well recognized that PM / DM is associated with increased risk of malignancy. The incidence of malignant tumors in patients with DM has been reported to be up to 24.4% (60/246) [18]. Tumors can develop prior to, simultaneously with, or after the occurrence of PM / DM. Most malignant tumors are identified within 1 year of PM / DM diagnosis [18–20], and malignance is an important prognostic factor for PM / DM patients [7]. The assessment of WBMRI in screening malignant tumors in patients with PM / DM has never been reported before. Our results demonstrated the value of WBMRI in screening malignancy. On the other hand, since the main purpose of WBMRI is to observe muscular involvement in patients with PM / DM. To shorten the scan time, applying to a larger scanner field, a thicker layer and interlayer space, as well as coronal scanning only, those resulted WBMRI was not sensitive to small lesions. In this study, the WBMRI failed to display the primary lesions in all 3 cases of nasopharyngeal cancer. Furthermore, a STIR single sequence scan also had difficulty in distinguishing between benign and malignant lesions.

The preferred therapy for PM / DM is corticosteroid, which is also one of the primary causes of osteonecrosis [11]. Osteonecrosis is a progressive and debilitating disease. Early clinical diagnosis and intervention are key for clinical prognosis [11, 21]. Thus, screening osteonecrosis in PM / DM patients has important clinical significance. Conventional regional MRI is the most sensitive imaging method for the diagnosis of osteonecrosis since it can identify signs of early osteonecrosis that are invisible on plain films and CT scans [22–25]. Early stages of osteonecrosis generally lack clinical symptoms, and steroid-induced osteonecrosis can occur at one or multiple parts of the body simultaneously. The conventional regional MRI examination can only cover a single site at one time. So, a complete screening of osteonecrosis requires multiple regional MRI examinations. Therefore, its application in screening steroid-induced osteonecrosis is limited. WBMRI scan covers the whole body. In this study, the WBMRI detected osteonecrosis in 15 patients, of whom the involvement of multiple joints was identified in 14 patients. Most joints shown to be involved with osteonecrosis had no clear clinical symptoms associated with osteonecrosis. Five patients with osteonecrosis underwent regional MRI, the findings of the regional MRI were consistent with that of the WBMRI, suggesting that WBMRI reveals muscular changes throughout the whole body and also displays early signs of osteonecrosis accurately.

There have been a few reports in recent years on the application of WBMRI in DM / PM diagnosis, but the number of cases was relatively small (the largest report included 41 patients), and the topic of those studies only concerned the performance of WBMRI in displaying muscular and subcutaneous tissue inflammation [7–9]. In this study, we performed a large case series of 129 patients to evaluate the performance of WBMRI on the diagnosis of both muscular and extra muscular changes, such as ILD, and systemic malignancy, as well as osteonecrosis in PM / DM patients.

This study has a few limitations. First, it was a single-center retrospective study. Secondly, Newly and previously diagnosed patients with PM / DM were included. Meanwhile, only 18 cases (14.0%) received follow-up WBMRI. Therefore, the number of PM / DM associated cancer and steroid therapy related osteonecrosis might be underestimated. Thirdly, we used only WBMRI and regional MRI to diagnose osteonecrosis since bone biopsies were considered invasive and impractical for multisite osteonecrosis. We used an MRI definition of osteonecrosis that is characterized by the presence of the classic band sign and has been validated by histological correlation [26].

In conclusion, WBMRI is a sensitive, non-invasive and efficient imaging method. It can provide a comprehensive assessment of muscular involvement in PM / DM patient cases, detect PM/DM associated extra muscular diseases such as ILD and systemic malignancy, and help screen steroid-induced osteonecrosis. Thus, it should be considered a promising examination for PM / DM diagnosis and follow-up.

## Supporting information

S1 Fig Coronal WBMRI imagings of Fig 1 patient. (ZIP)

S2 Fig Coronal WBMRI imagings of Fig 2 patient. (ZIP)

S3 Fig Coronal WBMRI imagings of Fig 3 patient. (ZIP)

S4 Fig Coronal WBMRI imagings of Fig 4 patient. (ZIP)

S5 Fig Coronal WBMRI imagings of Fig 5 patient. (ZIP)

S6 Fig Coronal WBMRI imagings of Fig 6 patient. (ZIP)
