# Supplementary figures and images for: An efficacy analysis of whole-body magnetic resonance imaging in the diagnosis and follow-up of polymyositis and dermatomyositis (part 1 of 2)
Source: PLoS One. 2017 Jul 17;12(7):e0181069. doi: 10.1371/journal.pone.0181069 (PMC5513424; doi:10.1371/journal.pone.0181069)

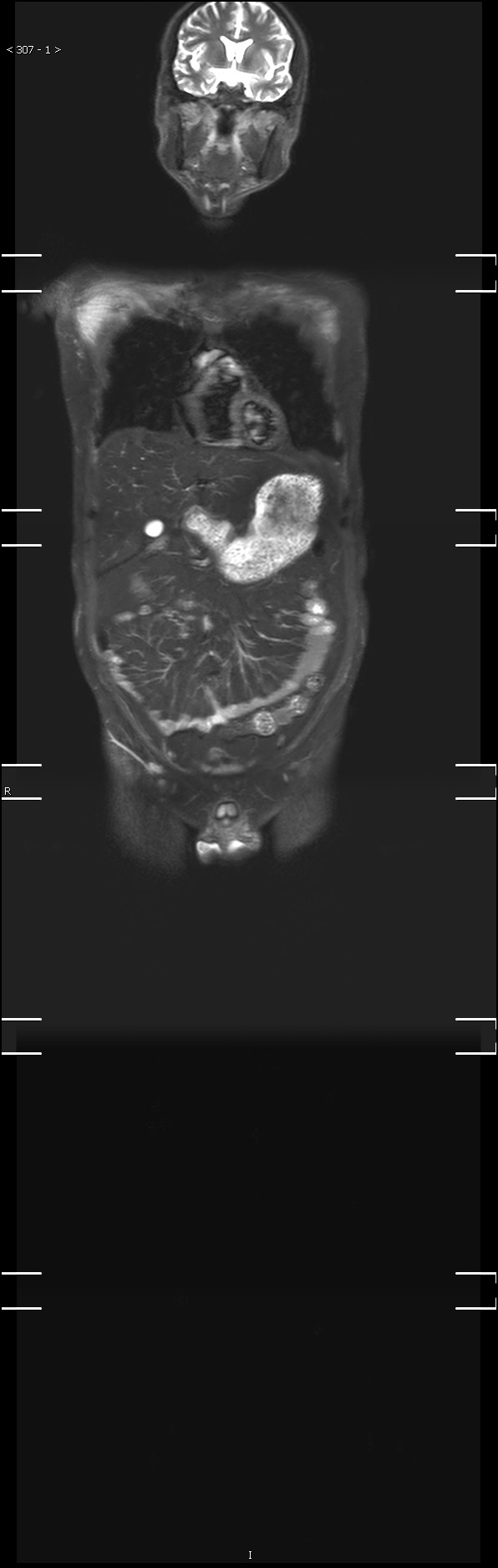

Supplement: S1 Fig — (ZIP) [file pone.0181069.s001.zip › S1/01.jpg]

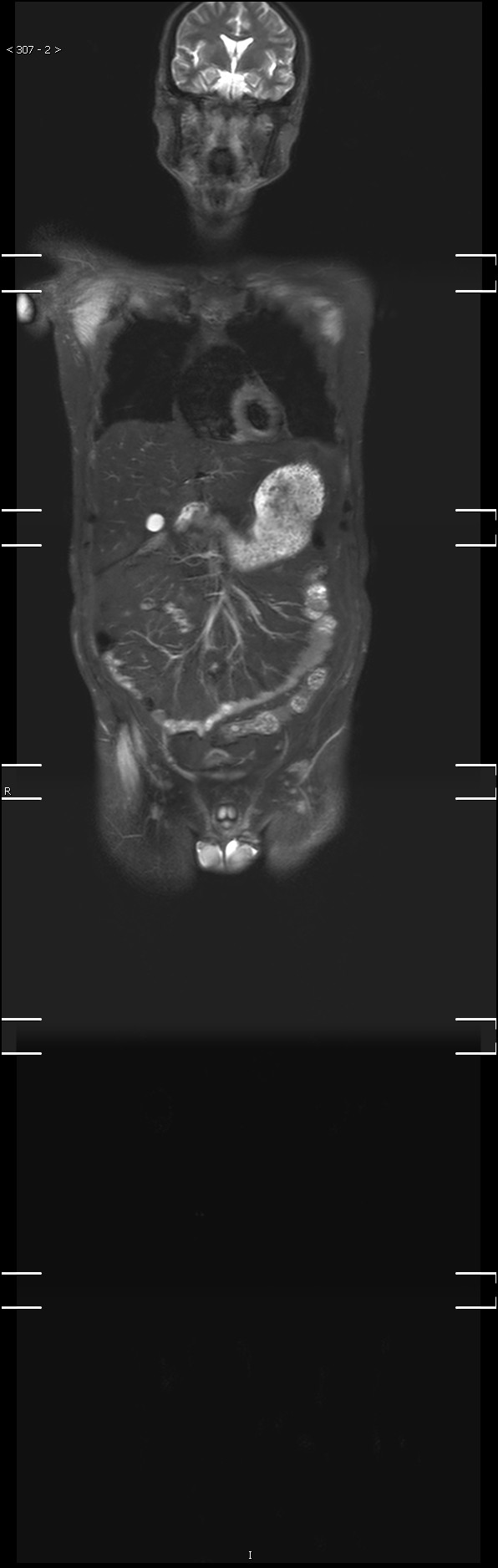

Supplement: S1 Fig — (ZIP) [file pone.0181069.s001.zip › S1/02.jpg]

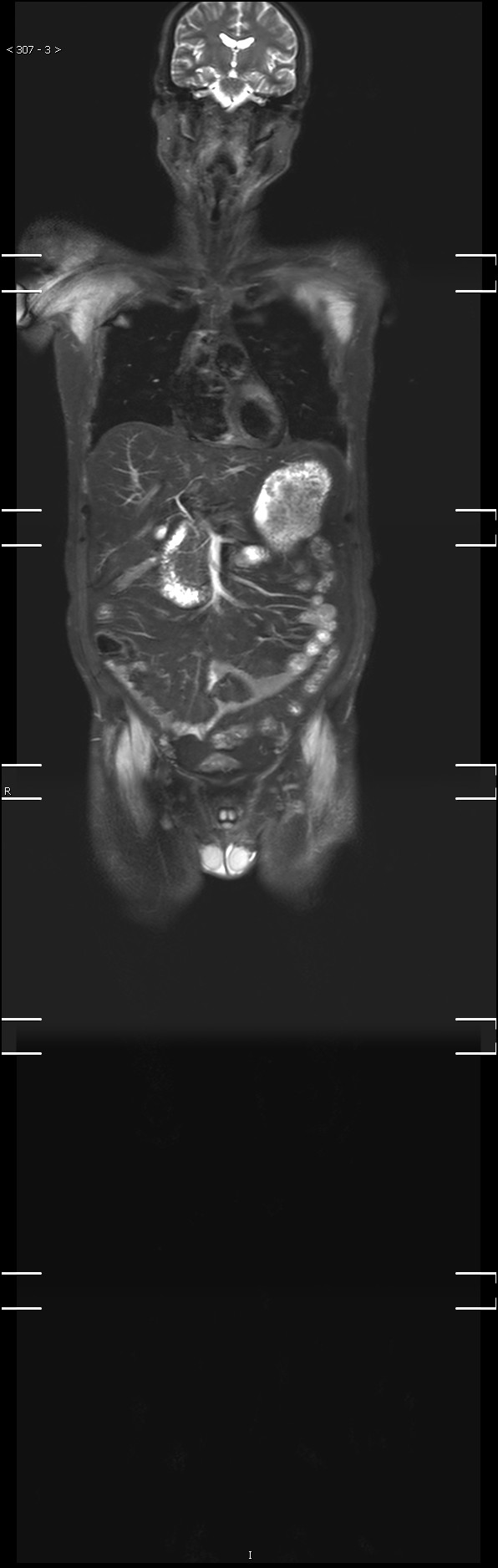

Supplement: S1 Fig — (ZIP) [file pone.0181069.s001.zip › S1/03.jpg]

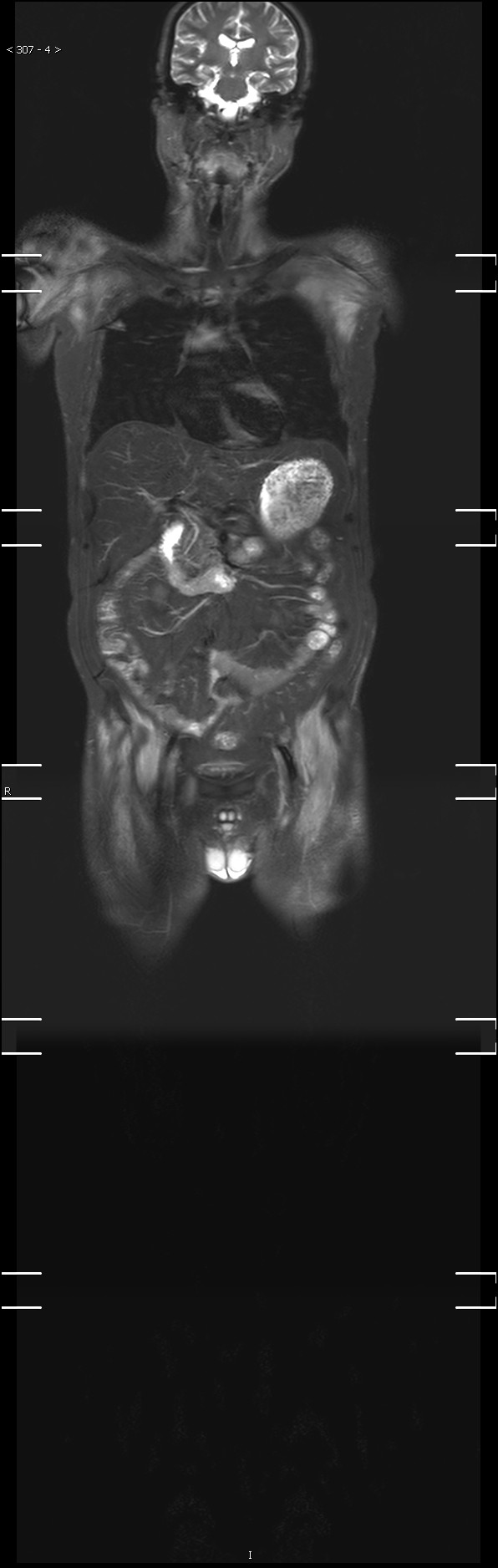

Supplement: S1 Fig — (ZIP) [file pone.0181069.s001.zip › S1/04.jpg]

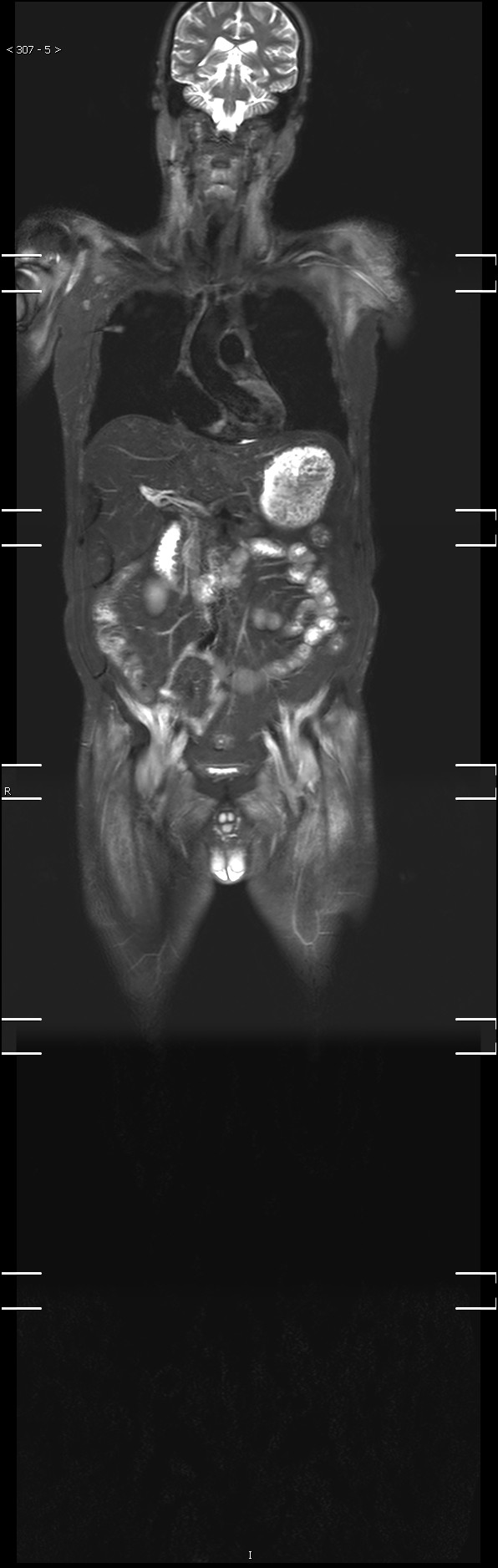

Supplement: S1 Fig — (ZIP) [file pone.0181069.s001.zip › S1/05.jpg]

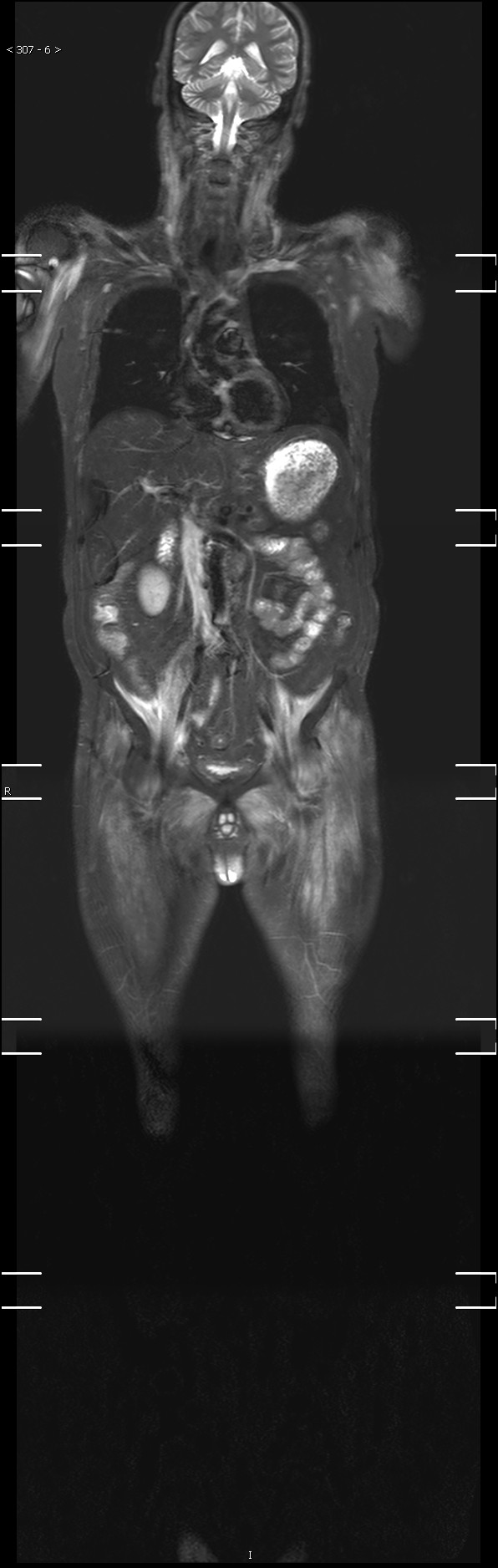

Supplement: S1 Fig — (ZIP) [file pone.0181069.s001.zip › S1/06.jpg]

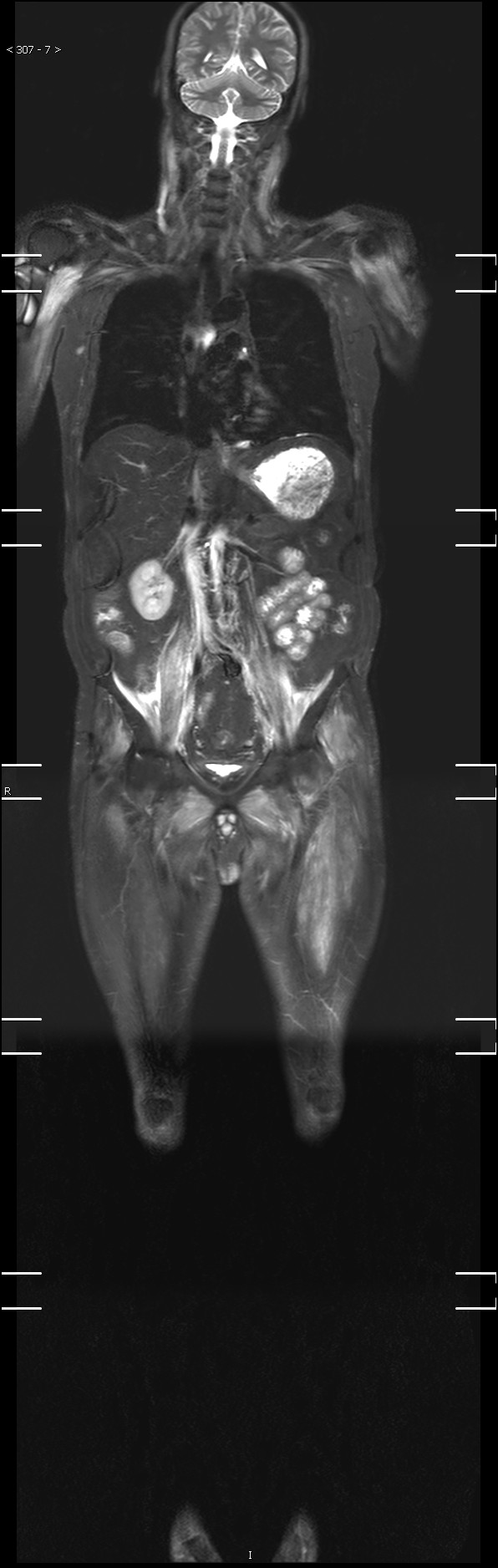

Supplement: S1 Fig — (ZIP) [file pone.0181069.s001.zip › S1/07.jpg]

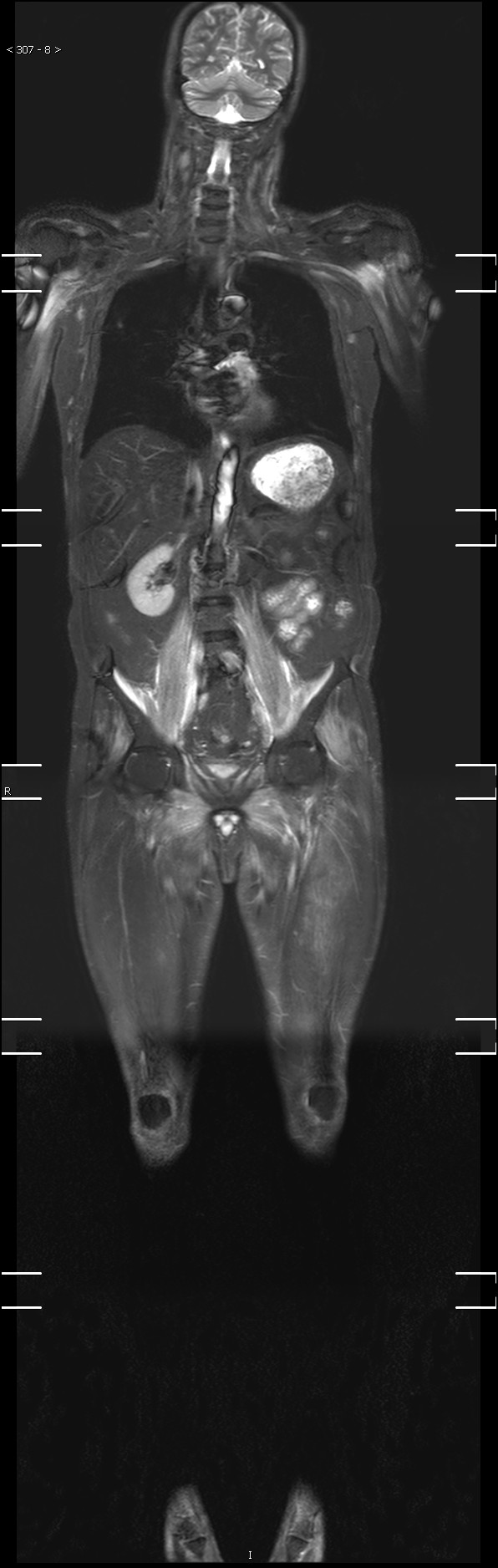

Supplement: S1 Fig — (ZIP) [file pone.0181069.s001.zip › S1/08.jpg]

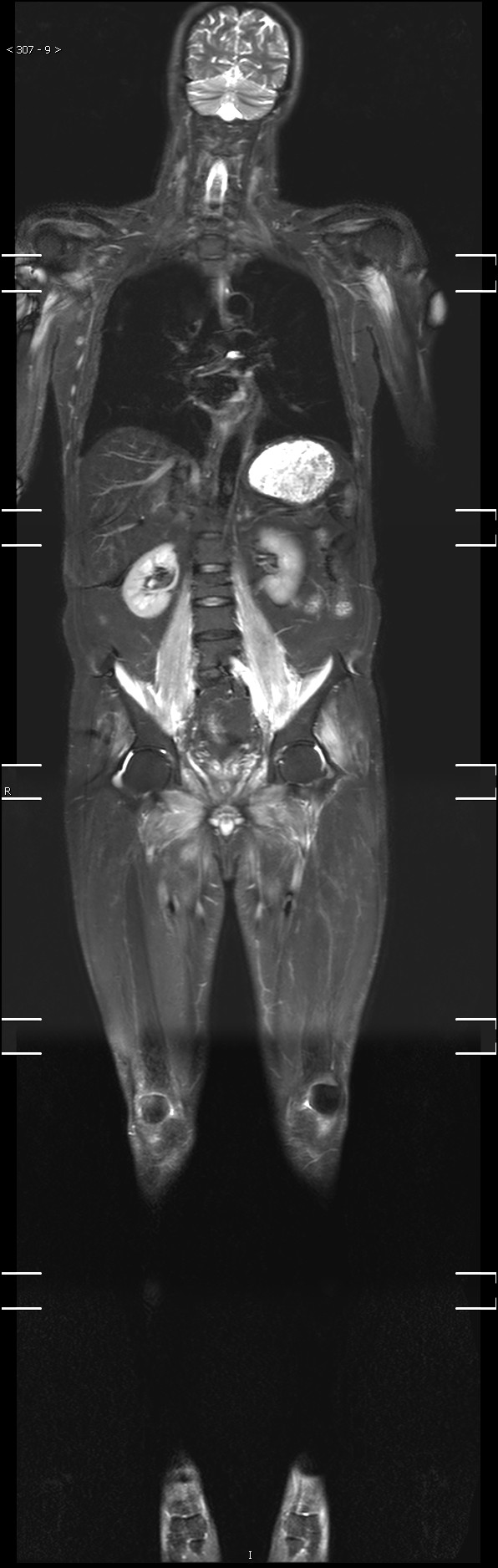

Supplement: S1 Fig — (ZIP) [file pone.0181069.s001.zip › S1/09.jpg]

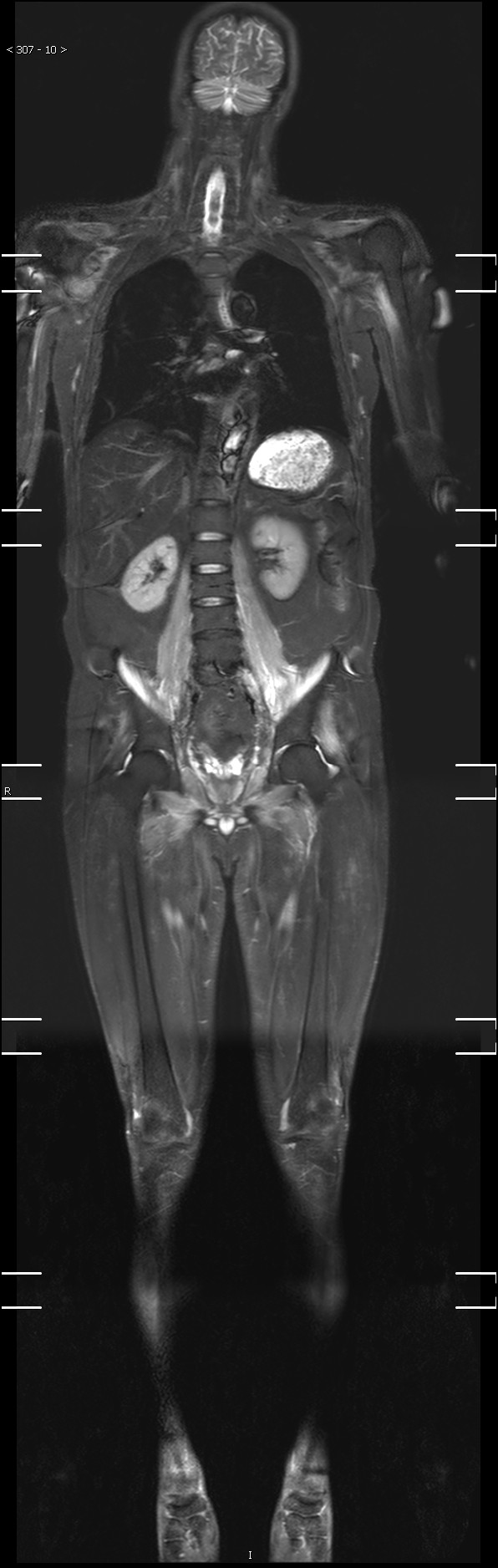

Supplement: S1 Fig — (ZIP) [file pone.0181069.s001.zip › S1/10.jpg]

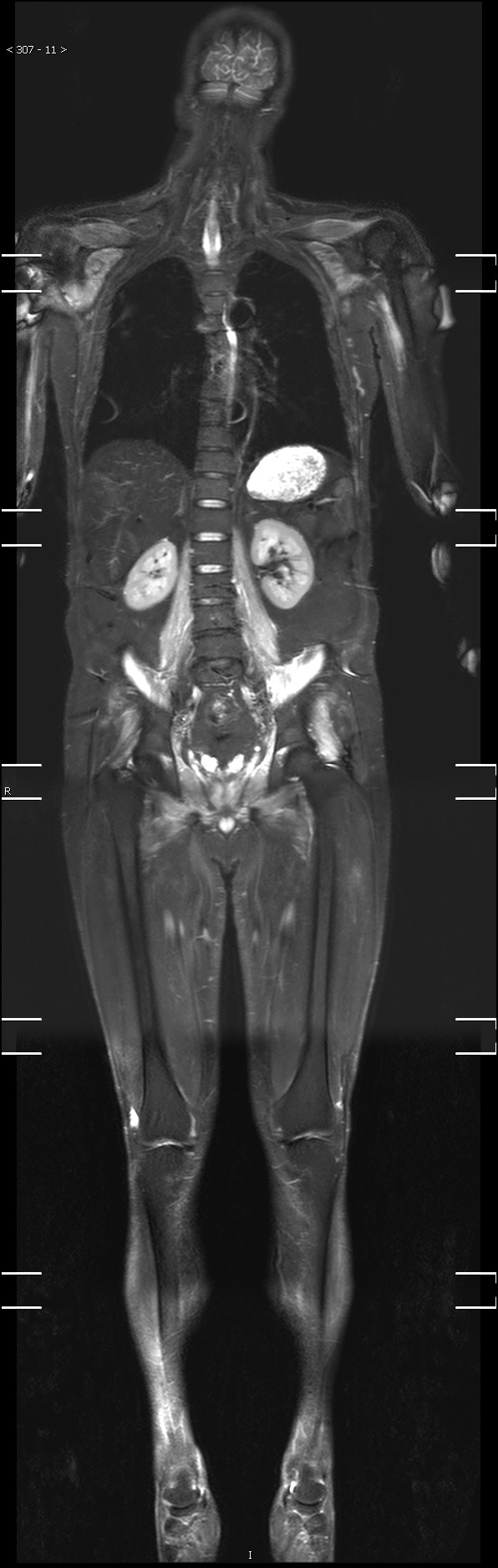

Supplement: S1 Fig — (ZIP) [file pone.0181069.s001.zip › S1/11.jpg]

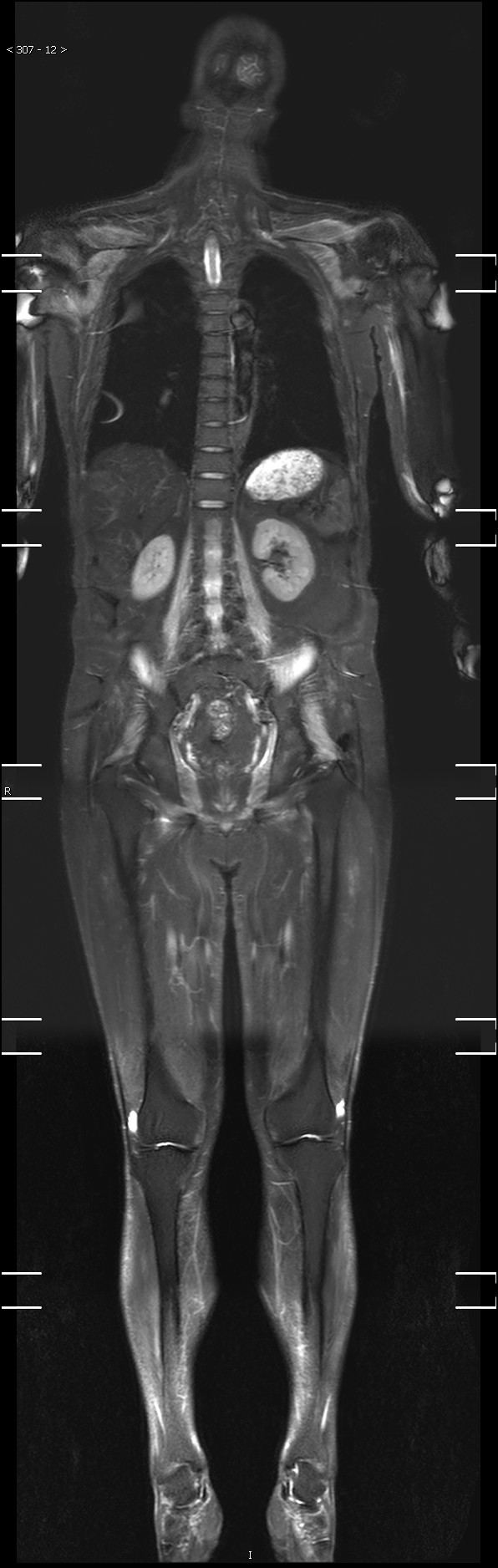

Supplement: S1 Fig — (ZIP) [file pone.0181069.s001.zip › S1/12.jpg]

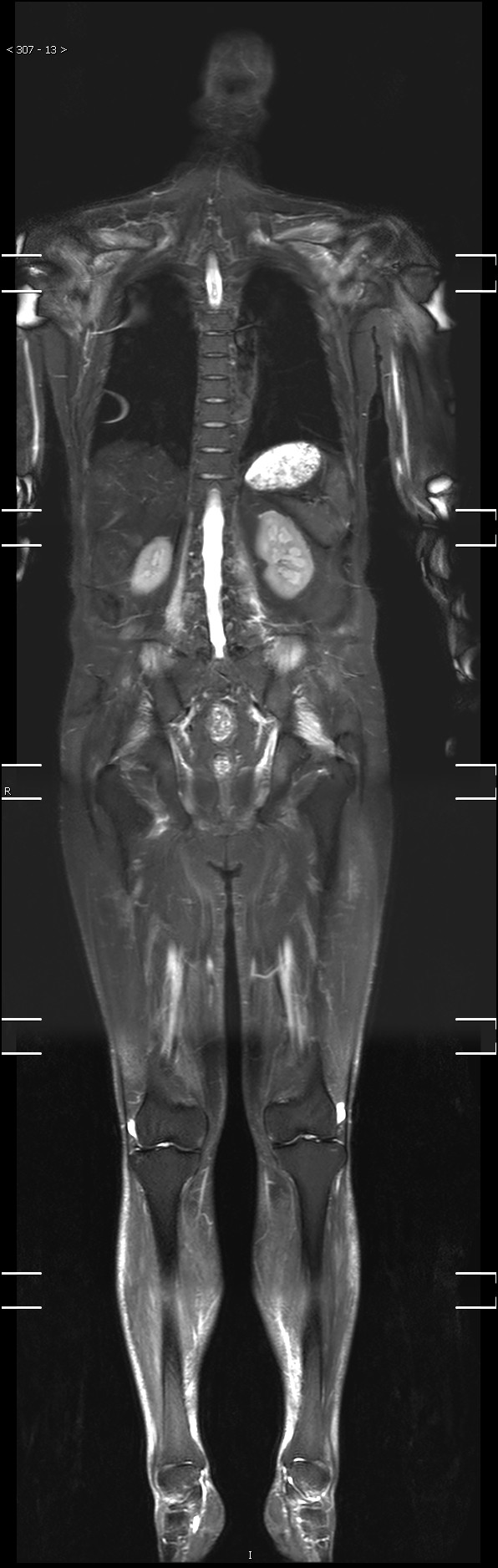

Supplement: S1 Fig — (ZIP) [file pone.0181069.s001.zip › S1/13.jpg]

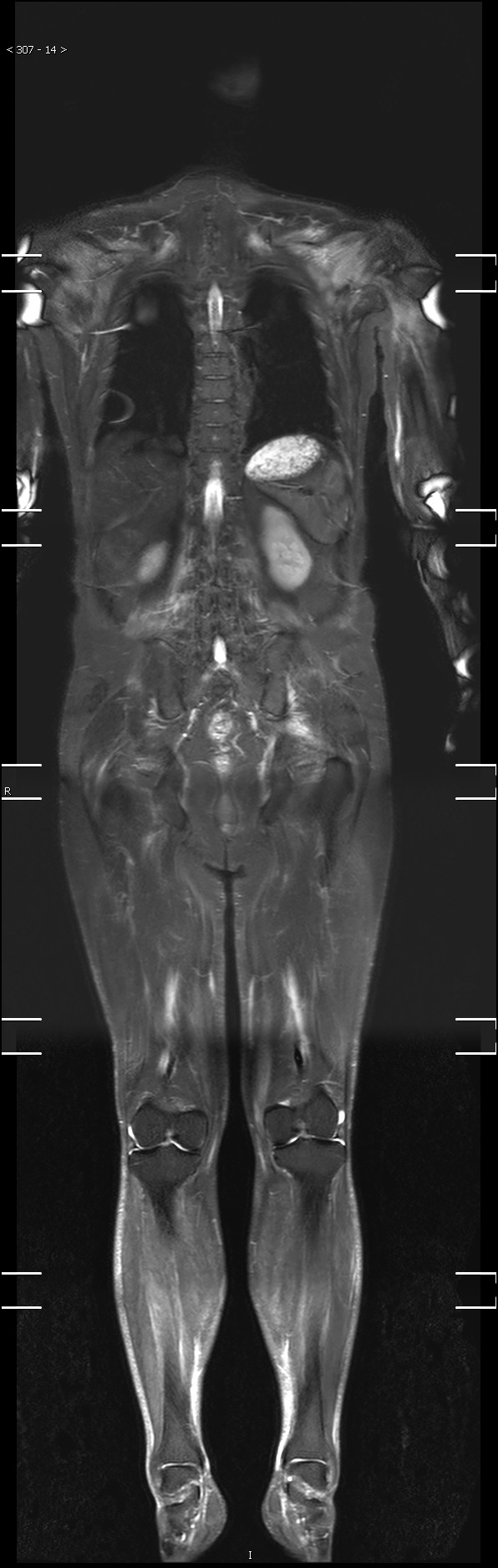

Supplement: S1 Fig — (ZIP) [file pone.0181069.s001.zip › S1/14.jpg]

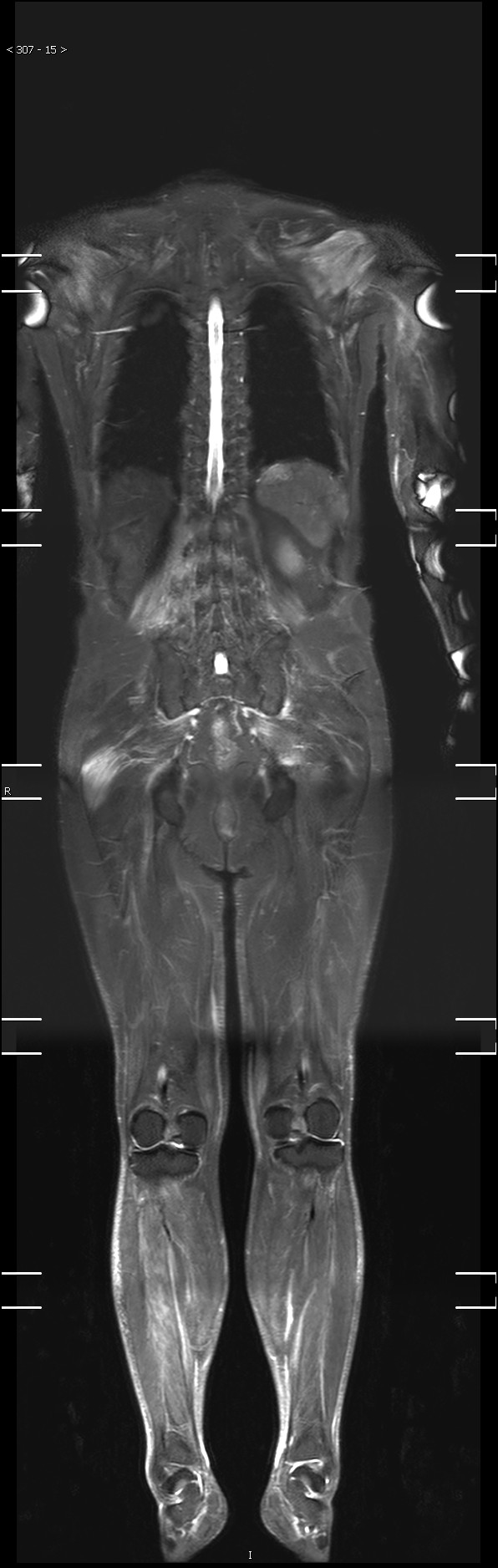

Supplement: S1 Fig — (ZIP) [file pone.0181069.s001.zip › S1/15.jpg]

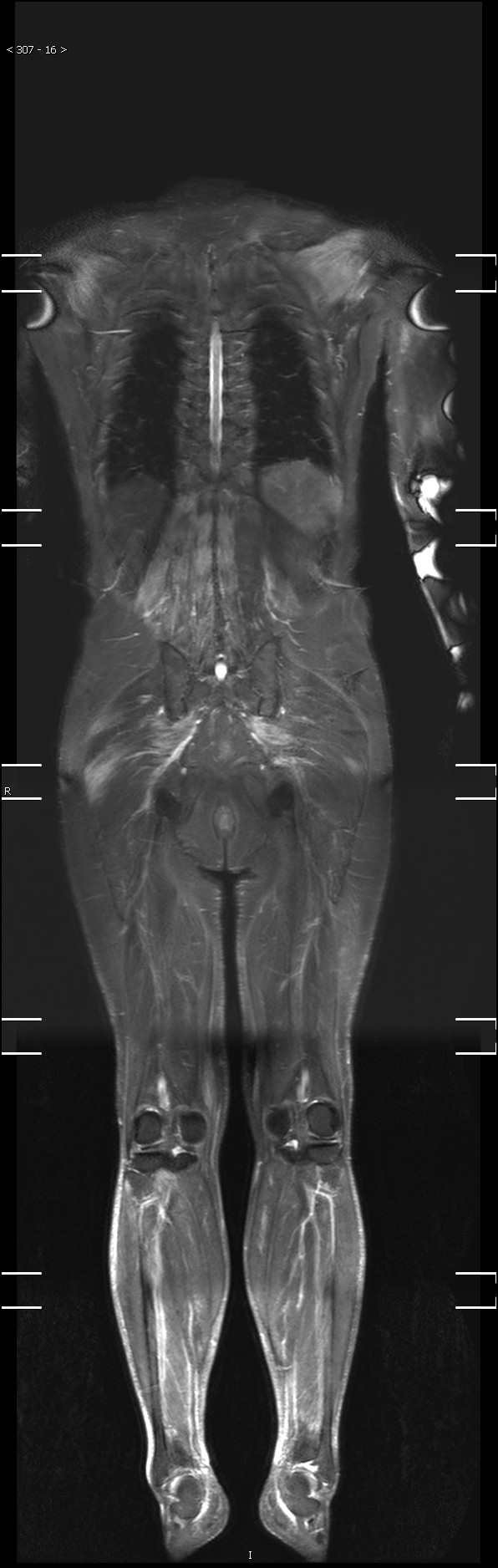

Supplement: S1 Fig — (ZIP) [file pone.0181069.s001.zip › S1/16.jpg]

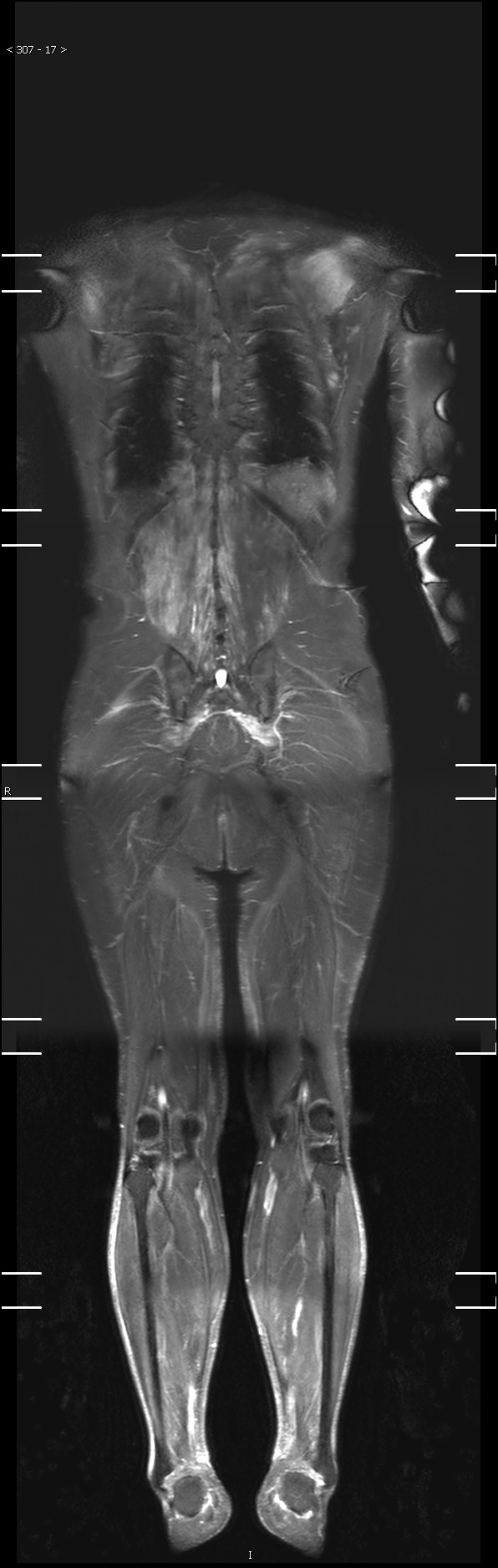

Supplement: S1 Fig — (ZIP) [file pone.0181069.s001.zip › S1/17.jpg]

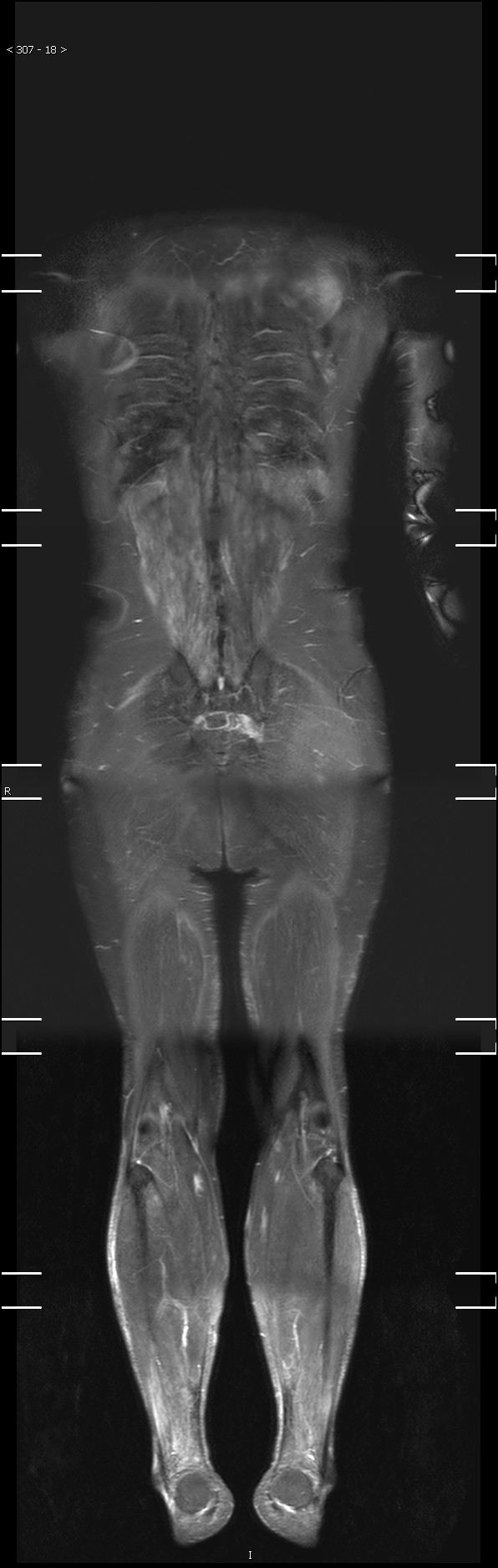

Supplement: S1 Fig — (ZIP) [file pone.0181069.s001.zip › S1/18.jpg]

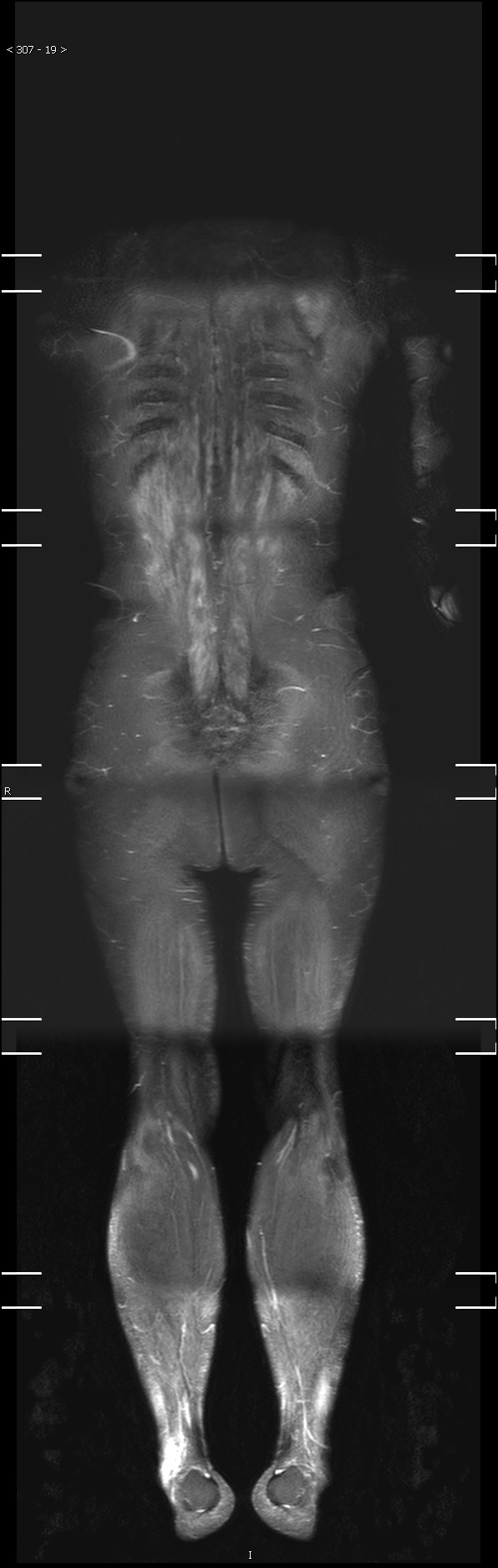

Supplement: S1 Fig — (ZIP) [file pone.0181069.s001.zip › S1/19.jpg]

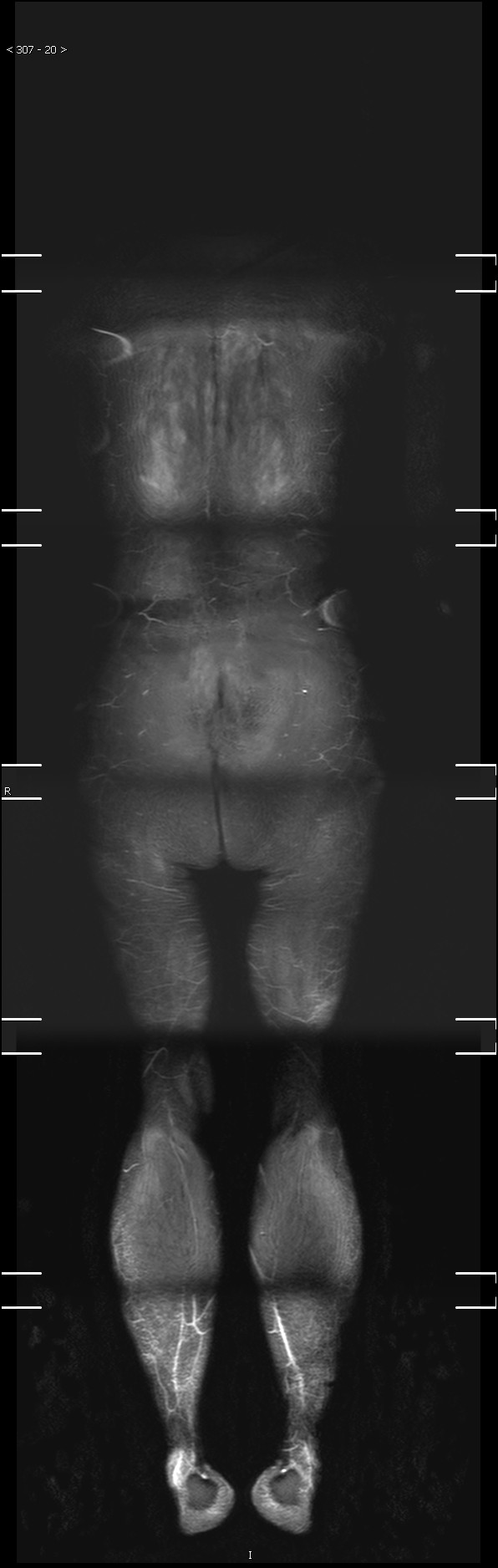

Supplement: S1 Fig — (ZIP) [file pone.0181069.s001.zip › S1/20.jpg]

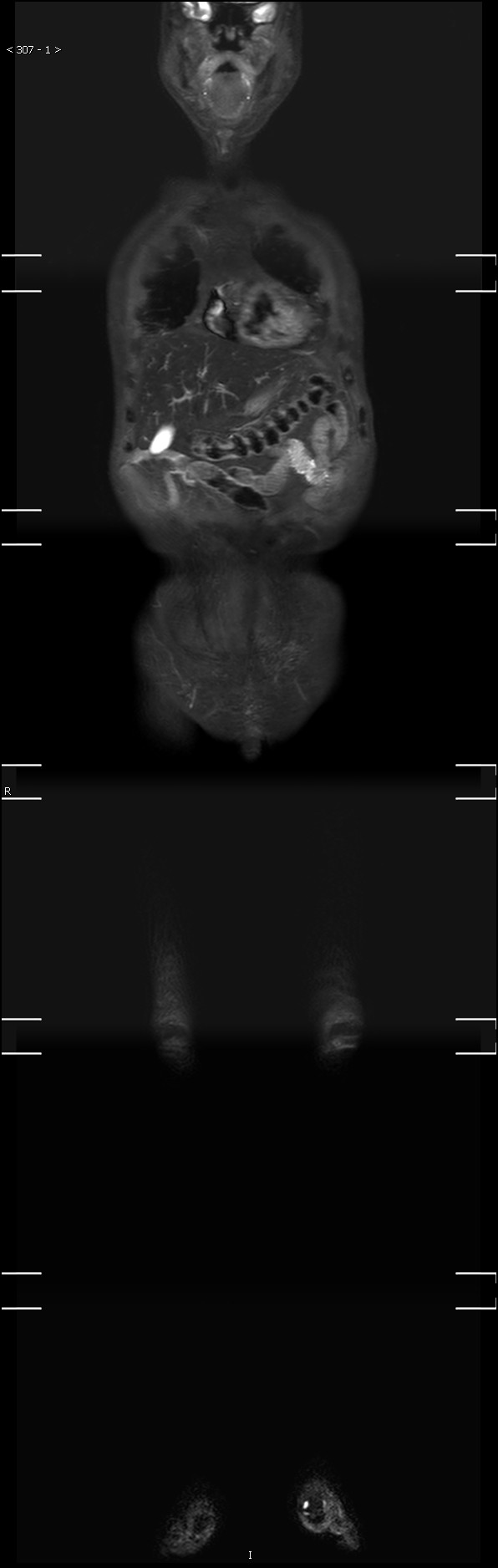

Supplement: S2 Fig — (ZIP) [file pone.0181069.s002.zip › S2/01.jpg]

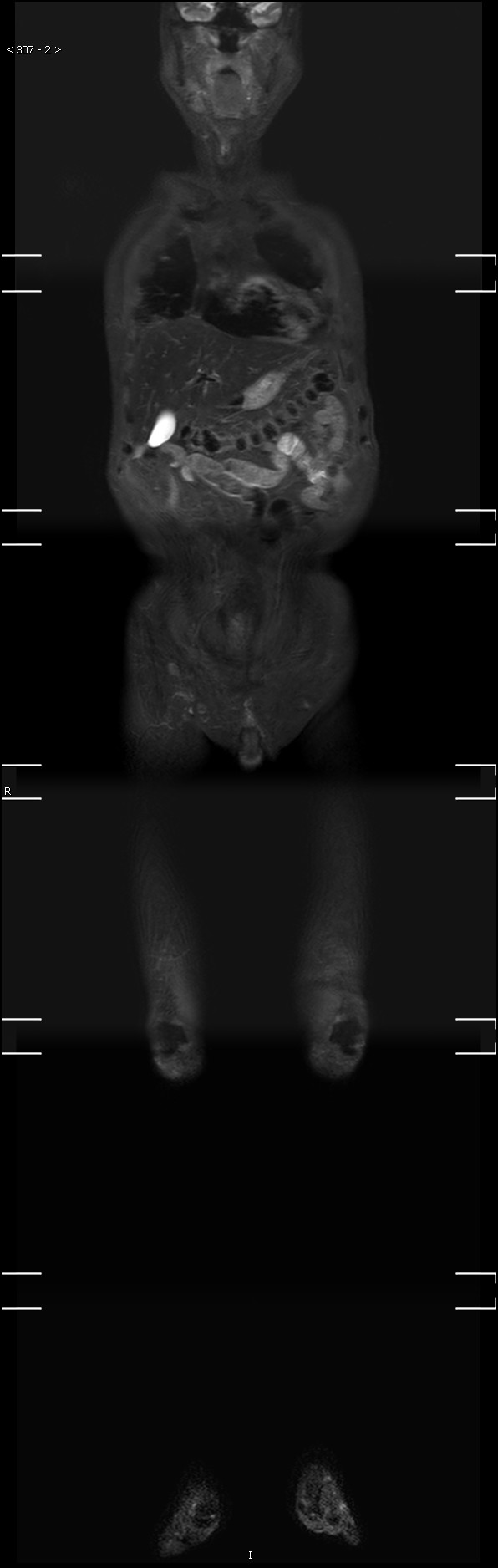

Supplement: S2 Fig — (ZIP) [file pone.0181069.s002.zip › S2/02.jpg]

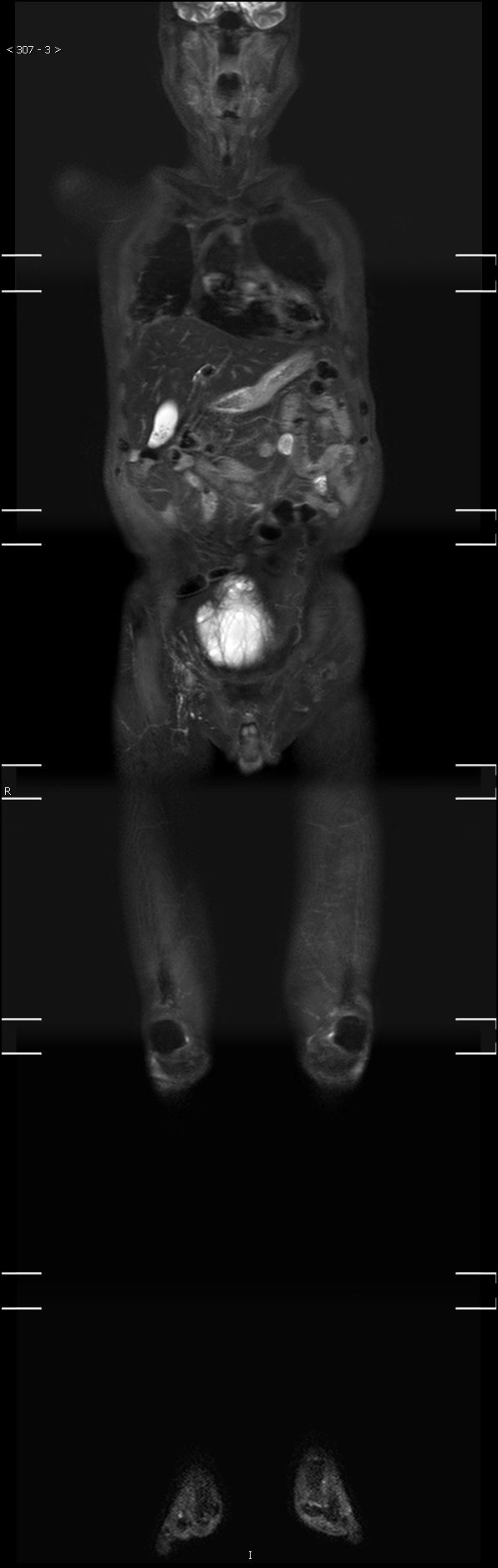

Supplement: S2 Fig — (ZIP) [file pone.0181069.s002.zip › S2/03.jpg]

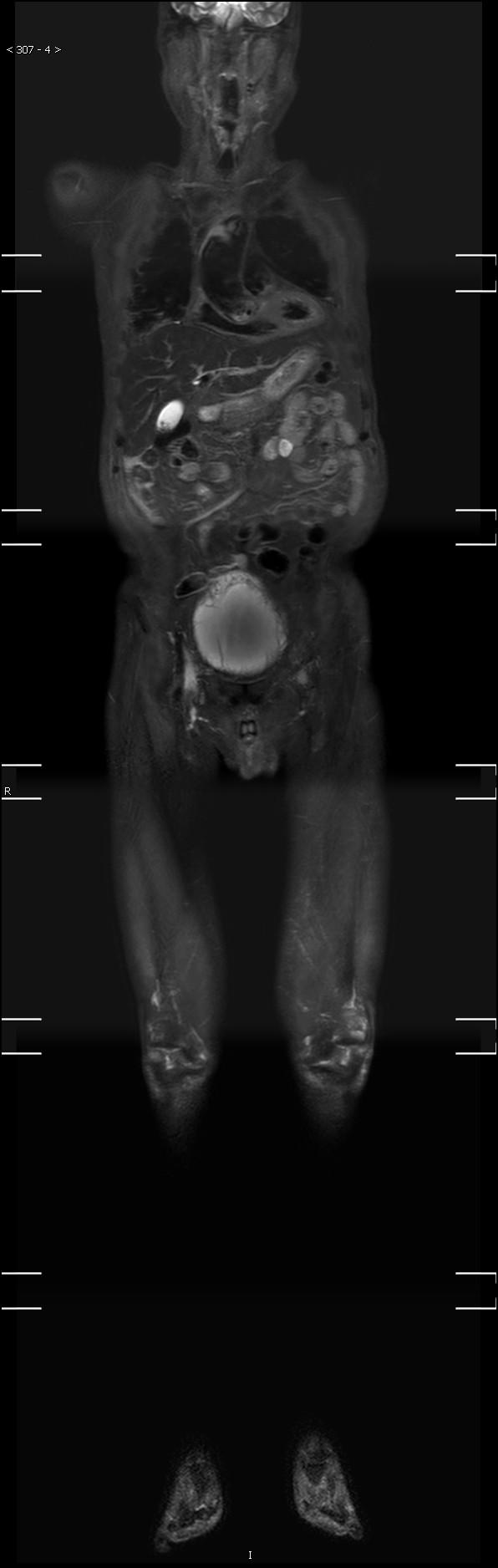

Supplement: S2 Fig — (ZIP) [file pone.0181069.s002.zip › S2/04.jpg]

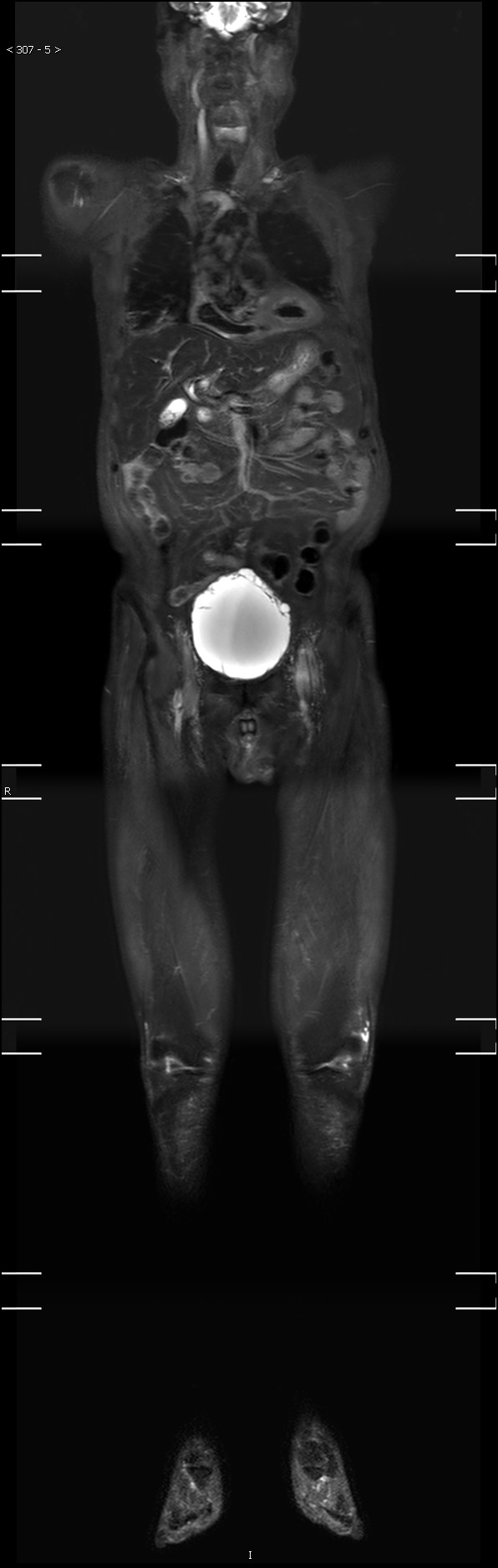

Supplement: S2 Fig — (ZIP) [file pone.0181069.s002.zip › S2/05.jpg]

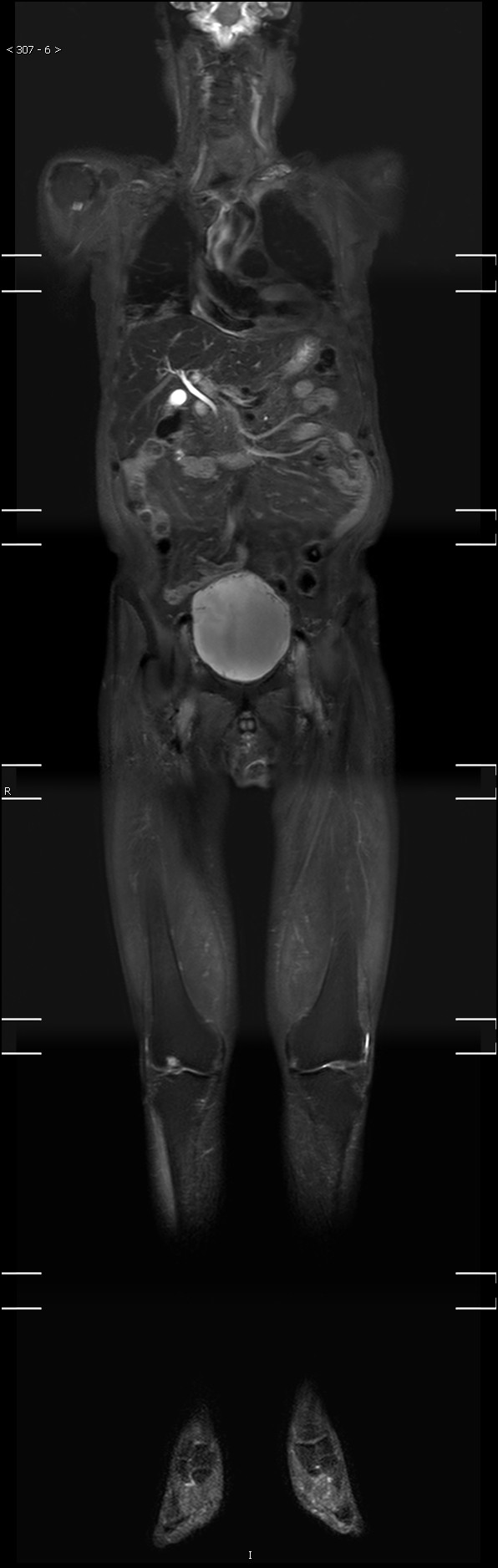

Supplement: S2 Fig — (ZIP) [file pone.0181069.s002.zip › S2/06.jpg]

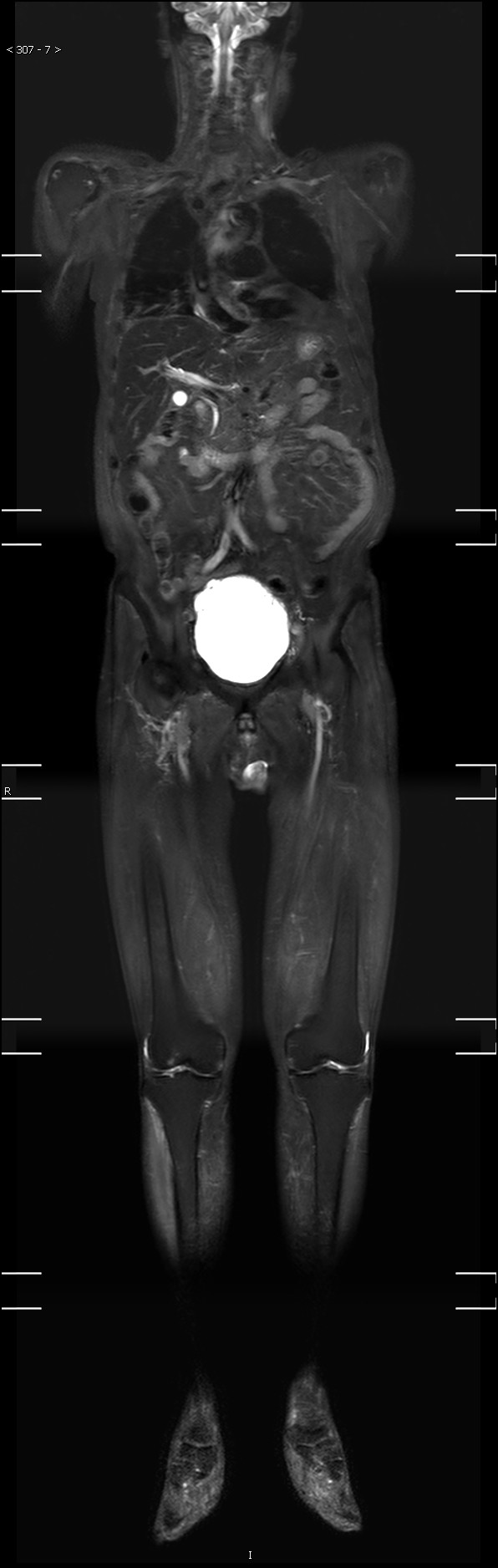

Supplement: S2 Fig — (ZIP) [file pone.0181069.s002.zip › S2/07.jpg]

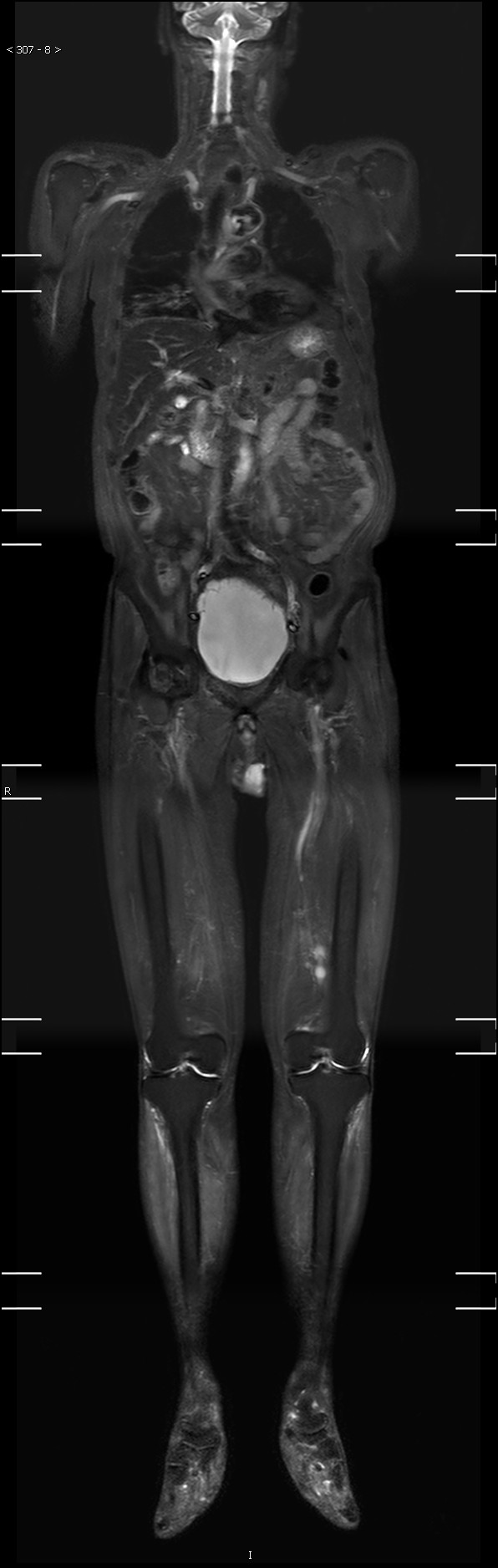

Supplement: S2 Fig — (ZIP) [file pone.0181069.s002.zip › S2/08.jpg]

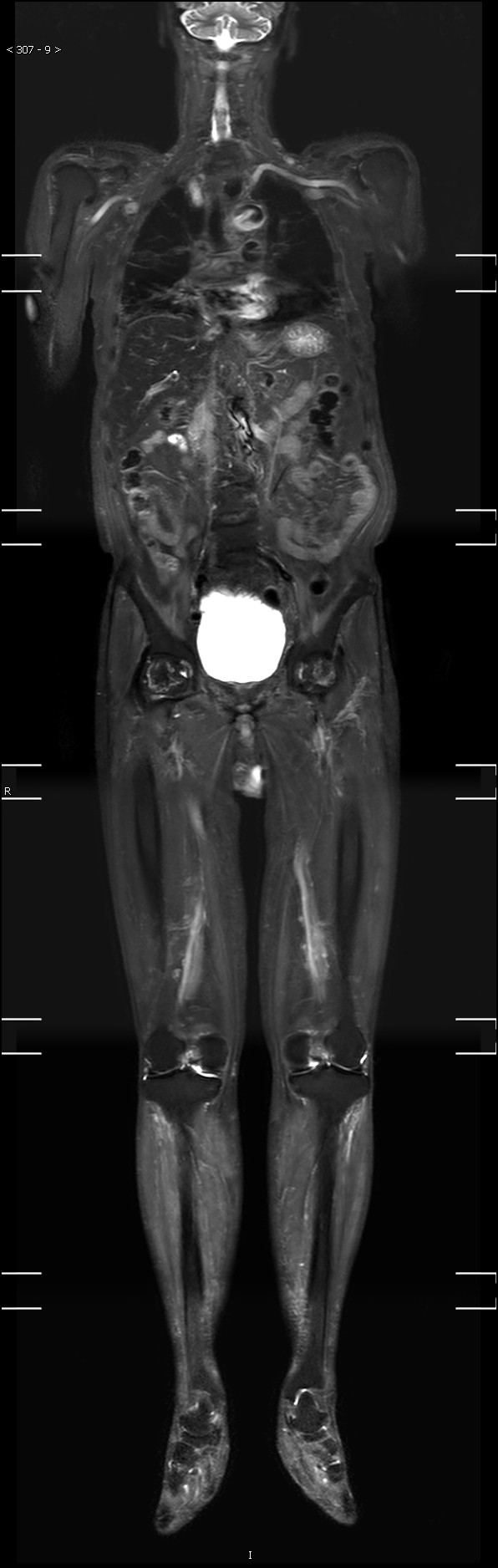

Supplement: S2 Fig — (ZIP) [file pone.0181069.s002.zip › S2/09.jpg]

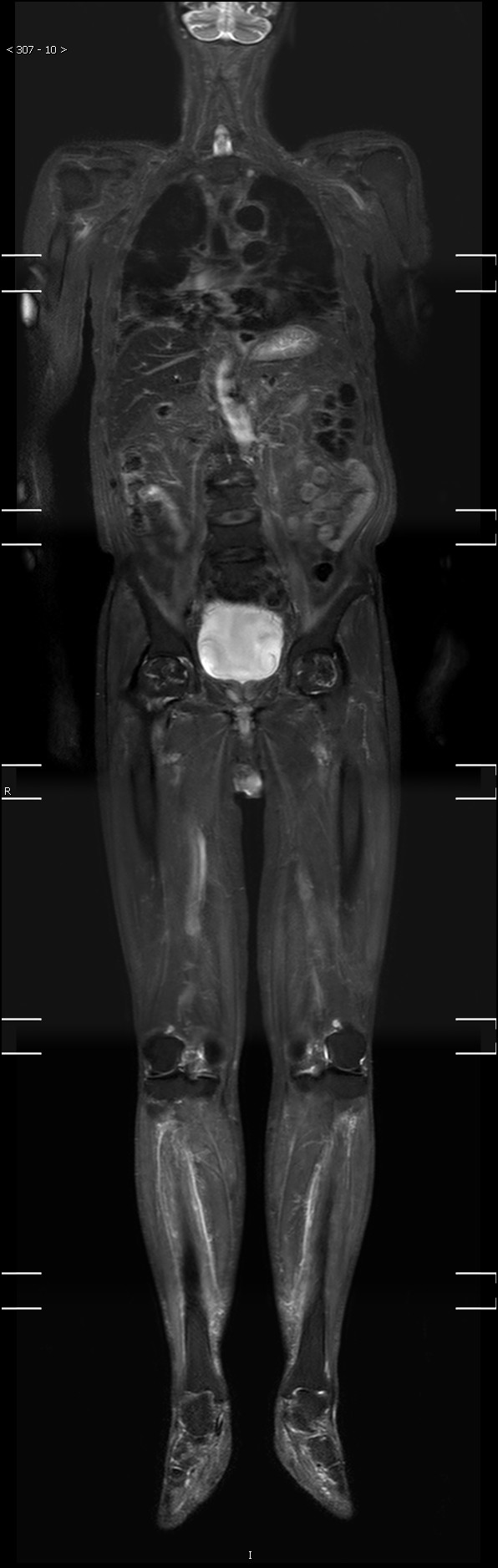

Supplement: S2 Fig — (ZIP) [file pone.0181069.s002.zip › S2/10.jpg]

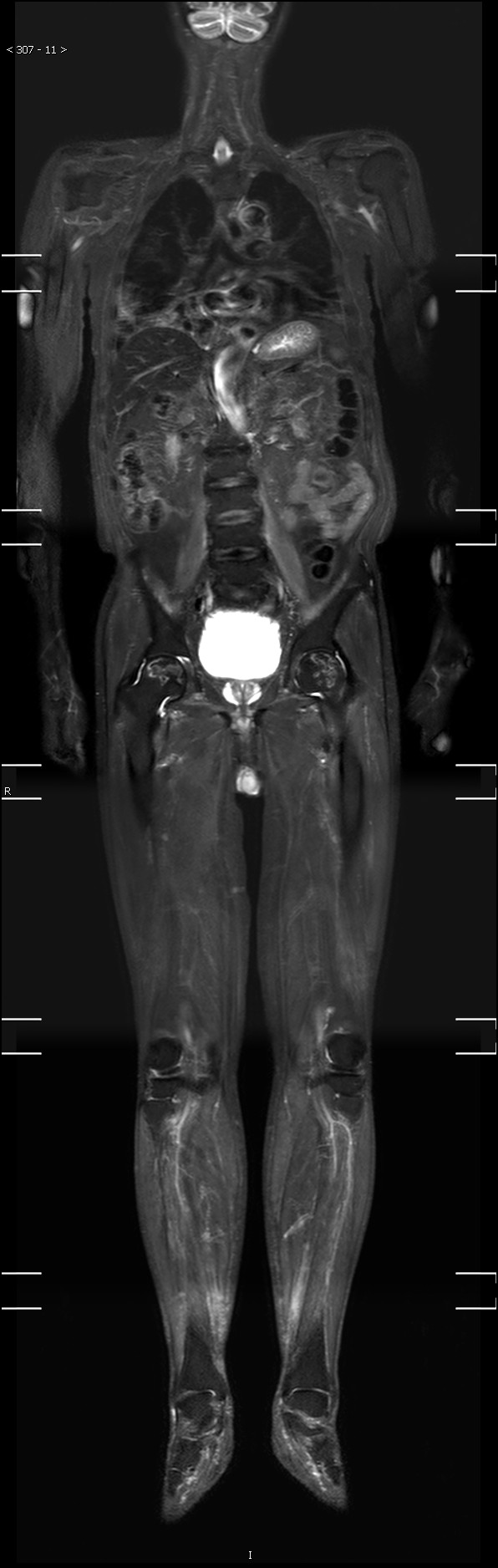

Supplement: S2 Fig — (ZIP) [file pone.0181069.s002.zip › S2/11.jpg]

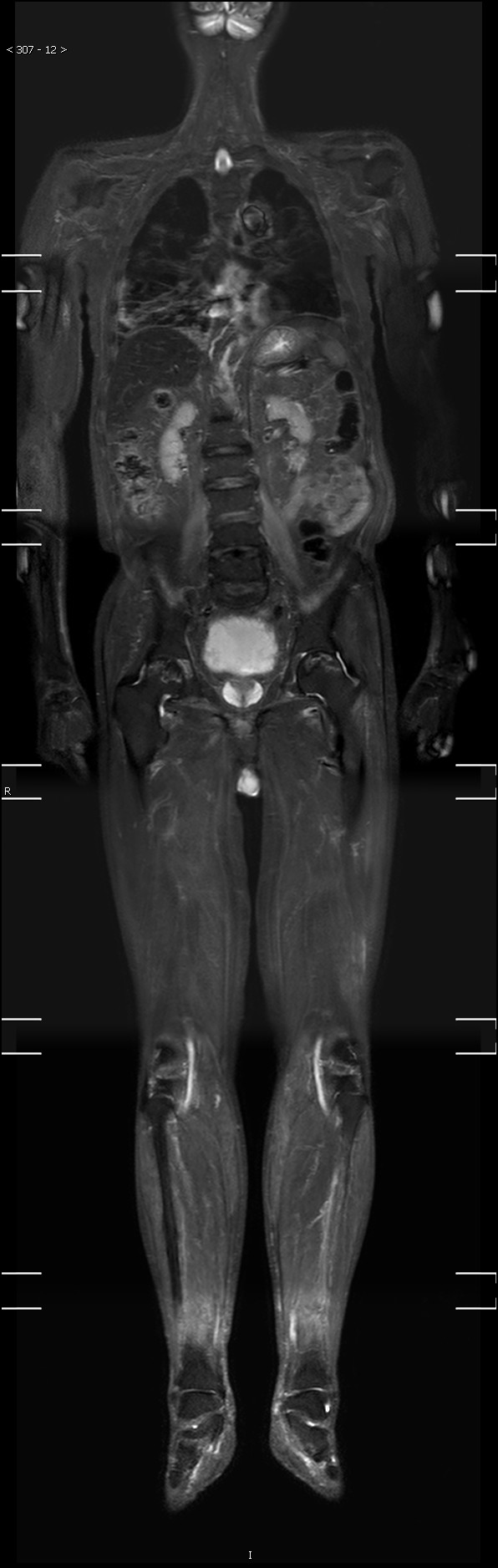

Supplement: S2 Fig — (ZIP) [file pone.0181069.s002.zip › S2/12.jpg]

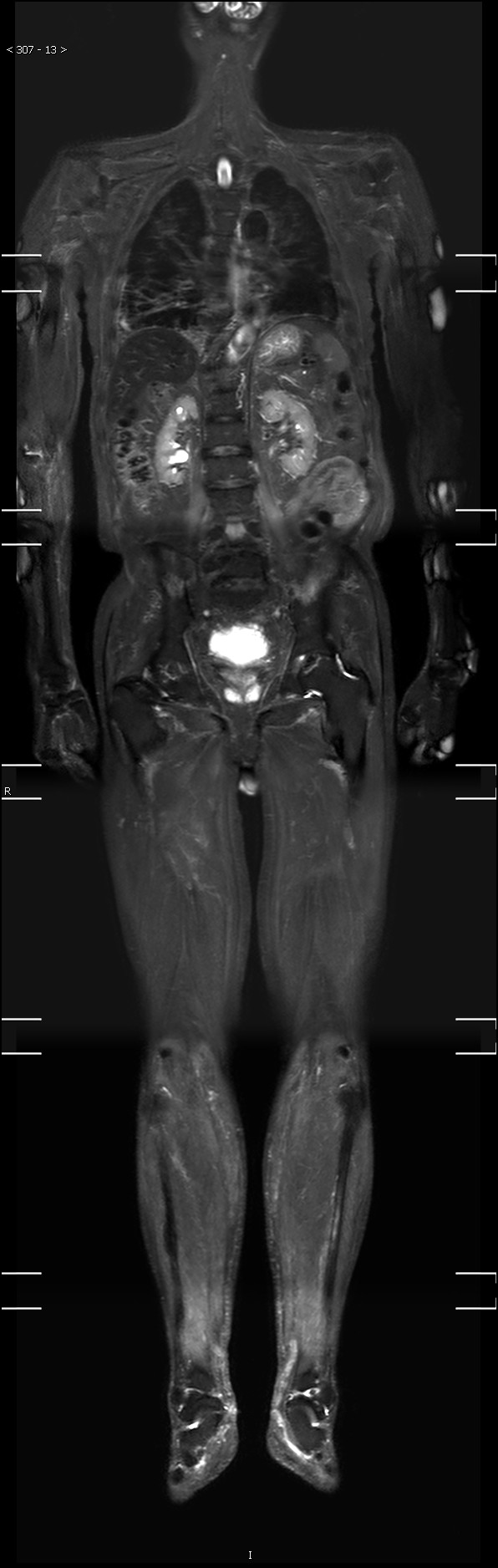

Supplement: S2 Fig — (ZIP) [file pone.0181069.s002.zip › S2/13.jpg]

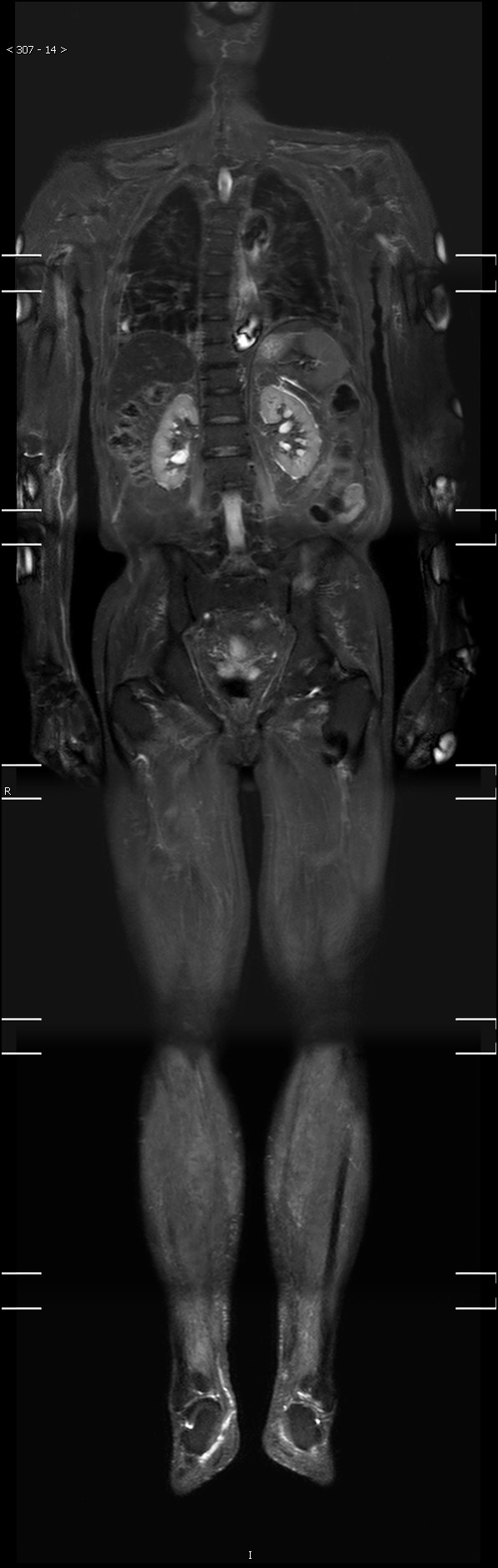

Supplement: S2 Fig — (ZIP) [file pone.0181069.s002.zip › S2/14.jpg]

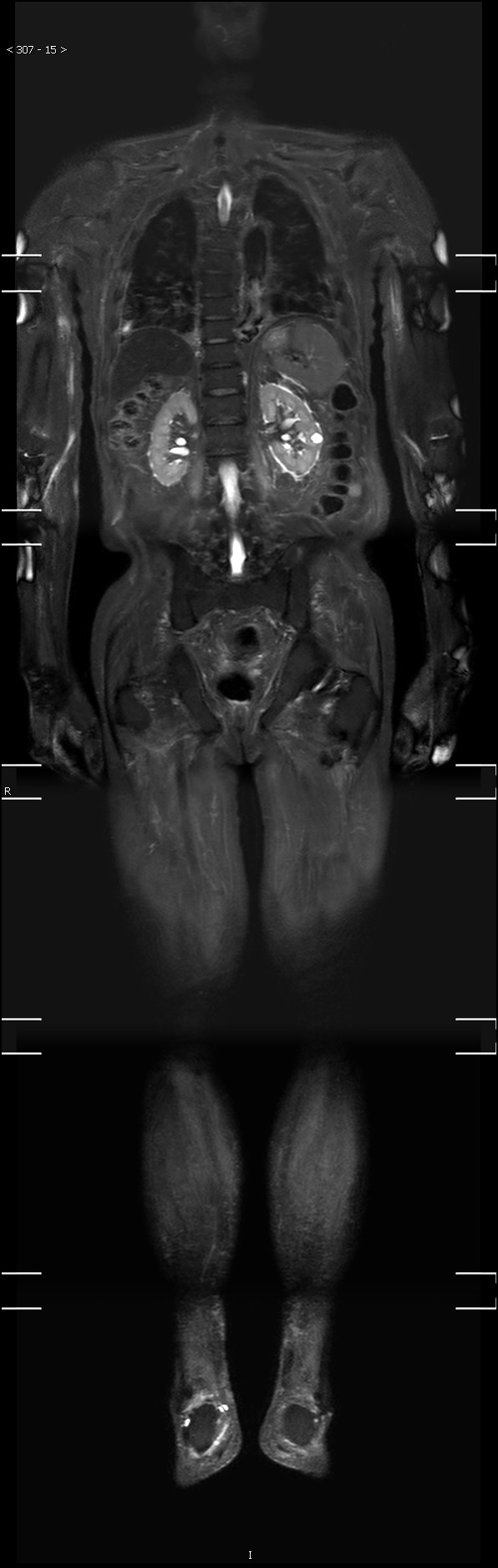

Supplement: S2 Fig — (ZIP) [file pone.0181069.s002.zip › S2/15.jpg]

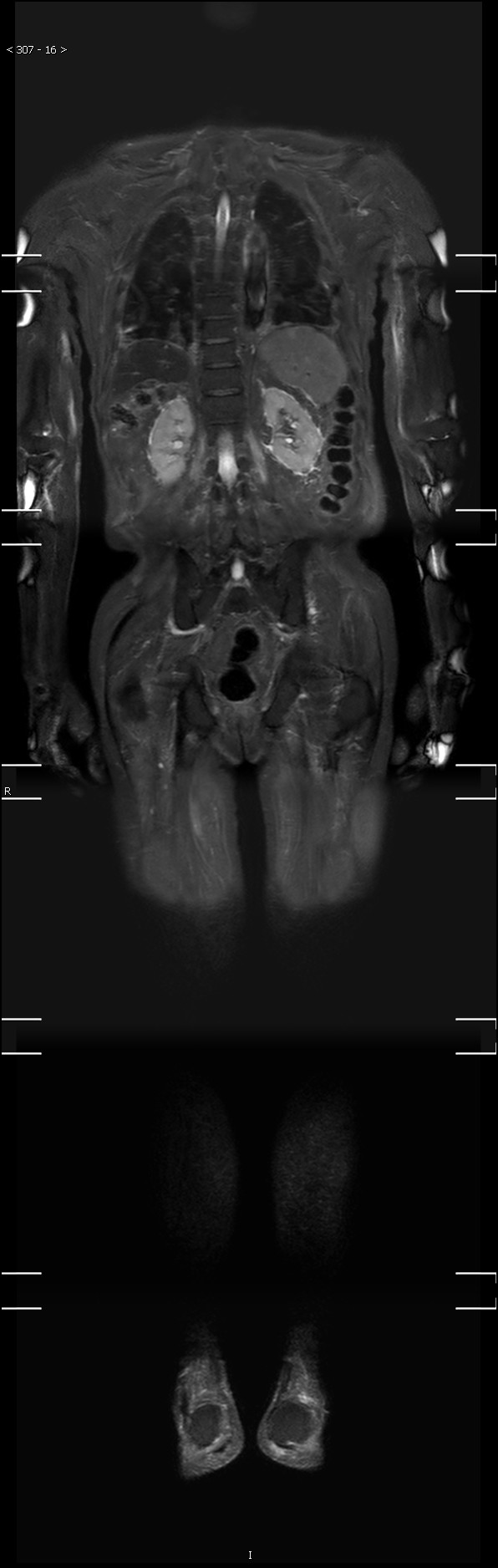

Supplement: S2 Fig — (ZIP) [file pone.0181069.s002.zip › S2/16.jpg]

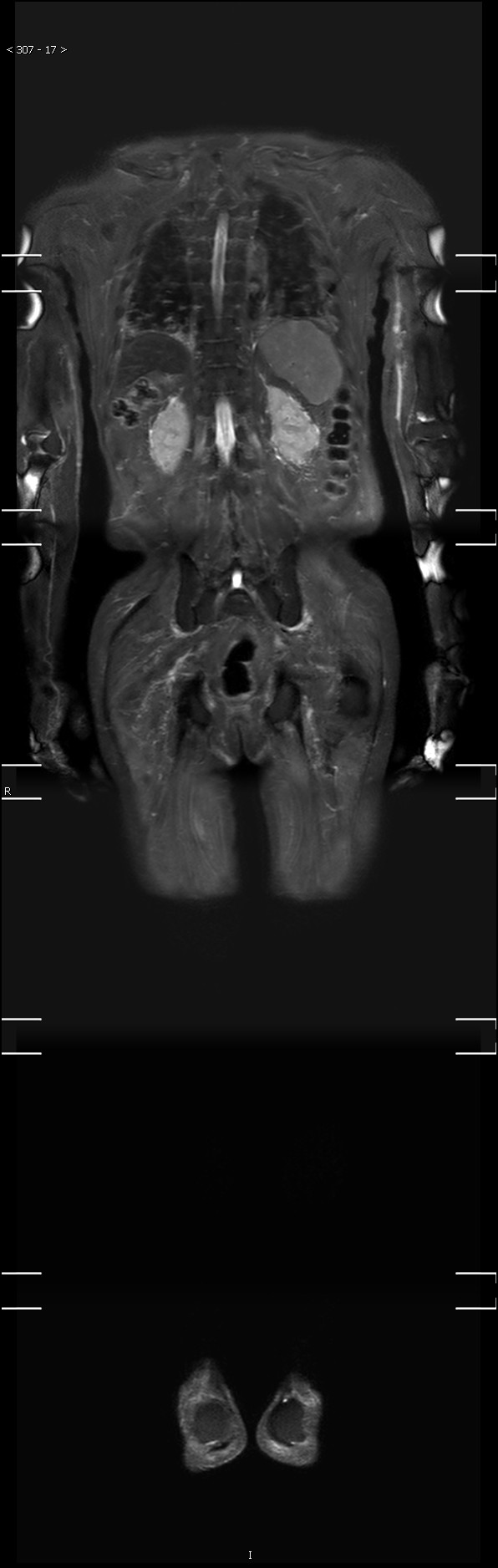

Supplement: S2 Fig — (ZIP) [file pone.0181069.s002.zip › S2/17.jpg]

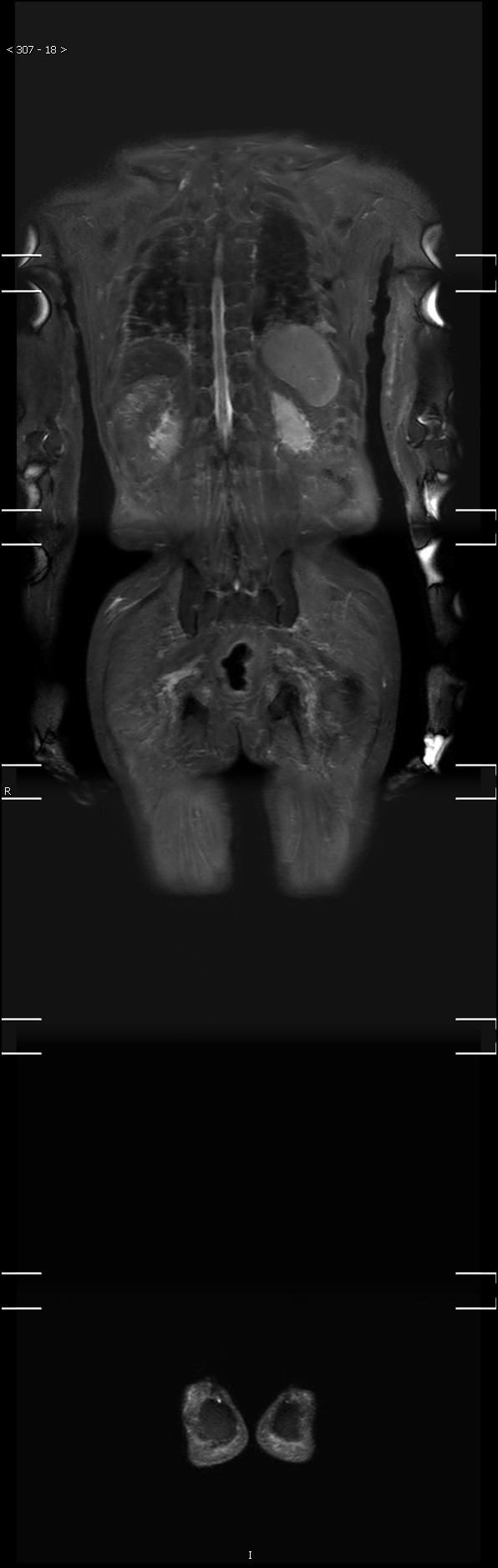

Supplement: S2 Fig — (ZIP) [file pone.0181069.s002.zip › S2/18.jpg]

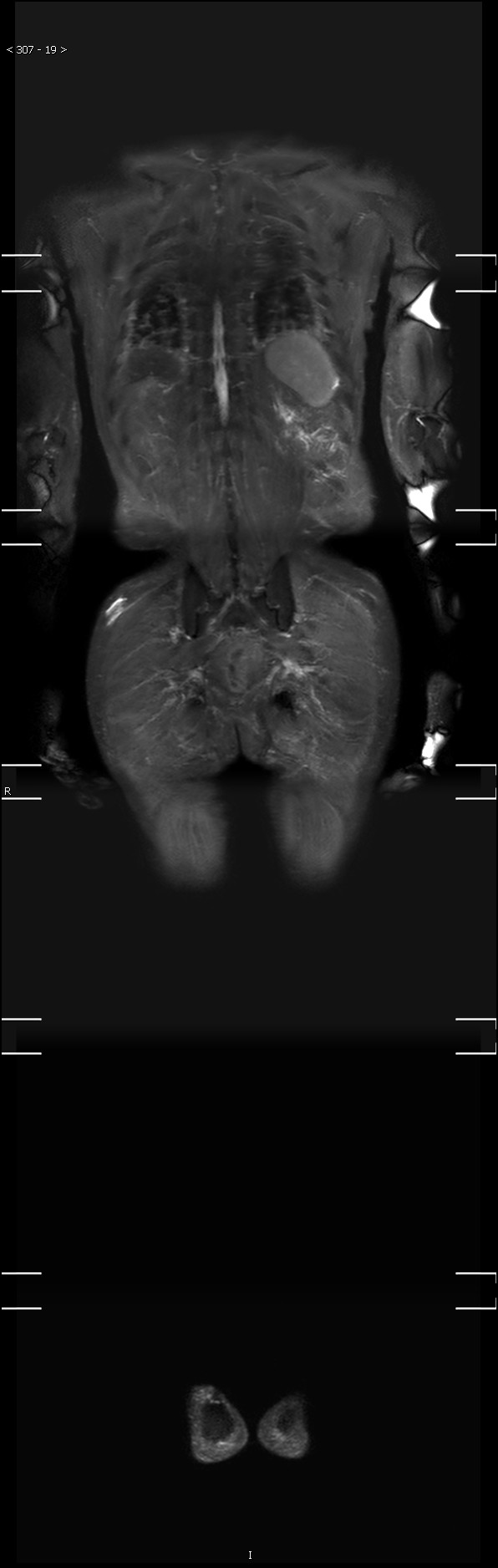

Supplement: S2 Fig — (ZIP) [file pone.0181069.s002.zip › S2/19.jpg]

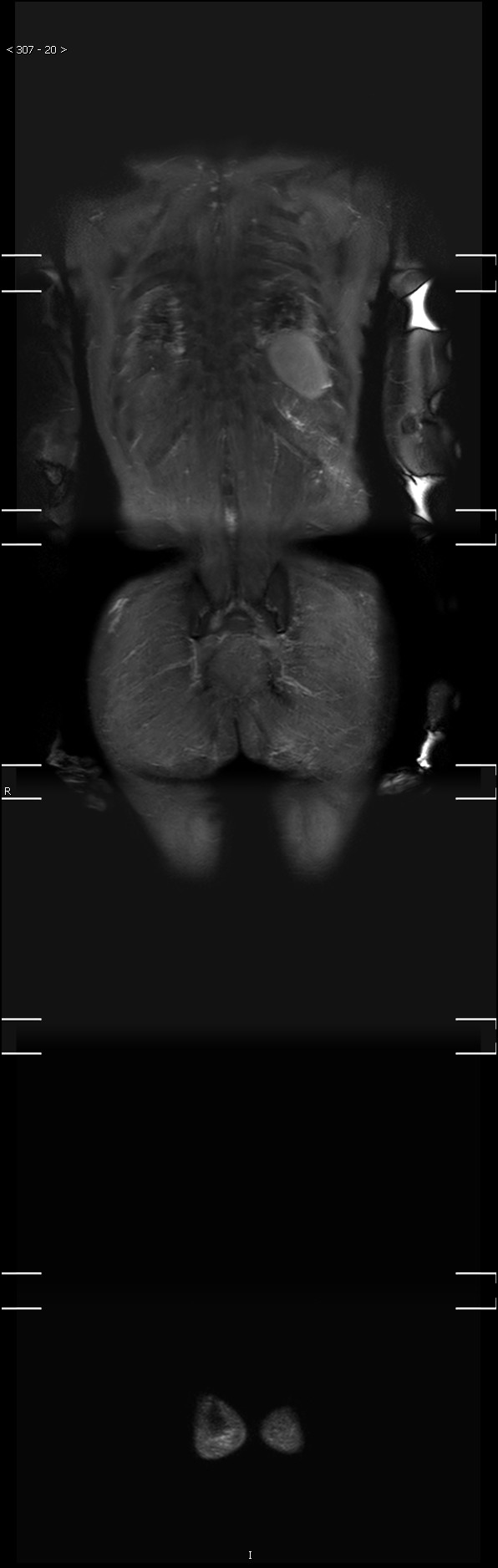

Supplement: S2 Fig — (ZIP) [file pone.0181069.s002.zip › S2/20.jpg]

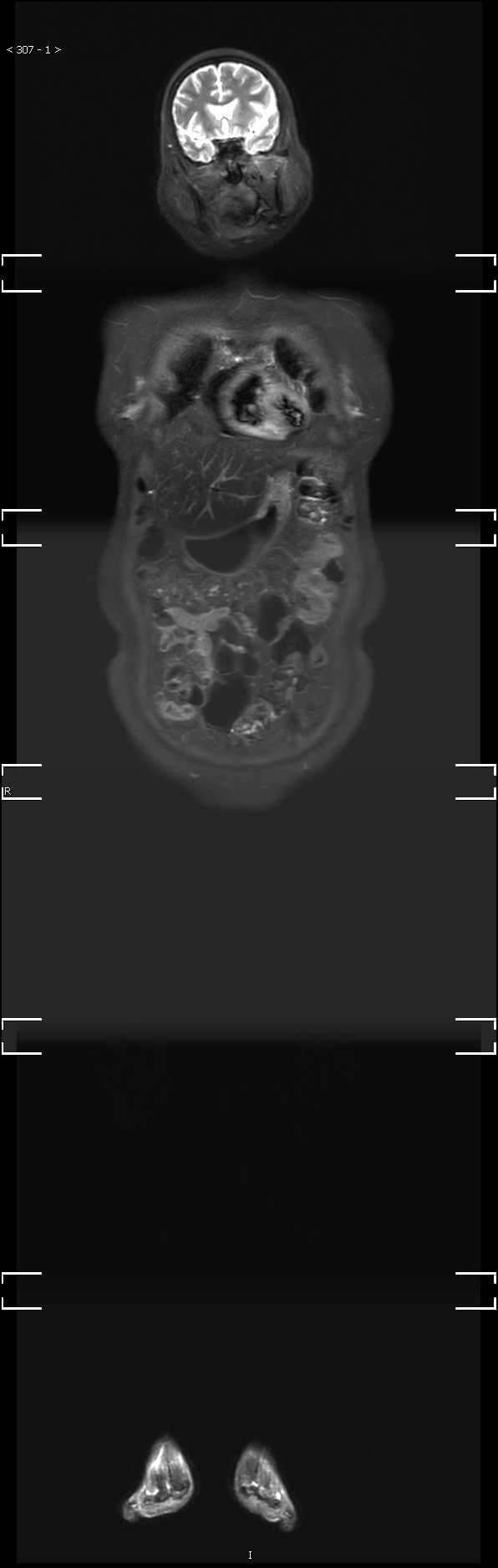

Supplement: S3 Fig — (ZIP) [file pone.0181069.s003.zip › S3/01.jpg]

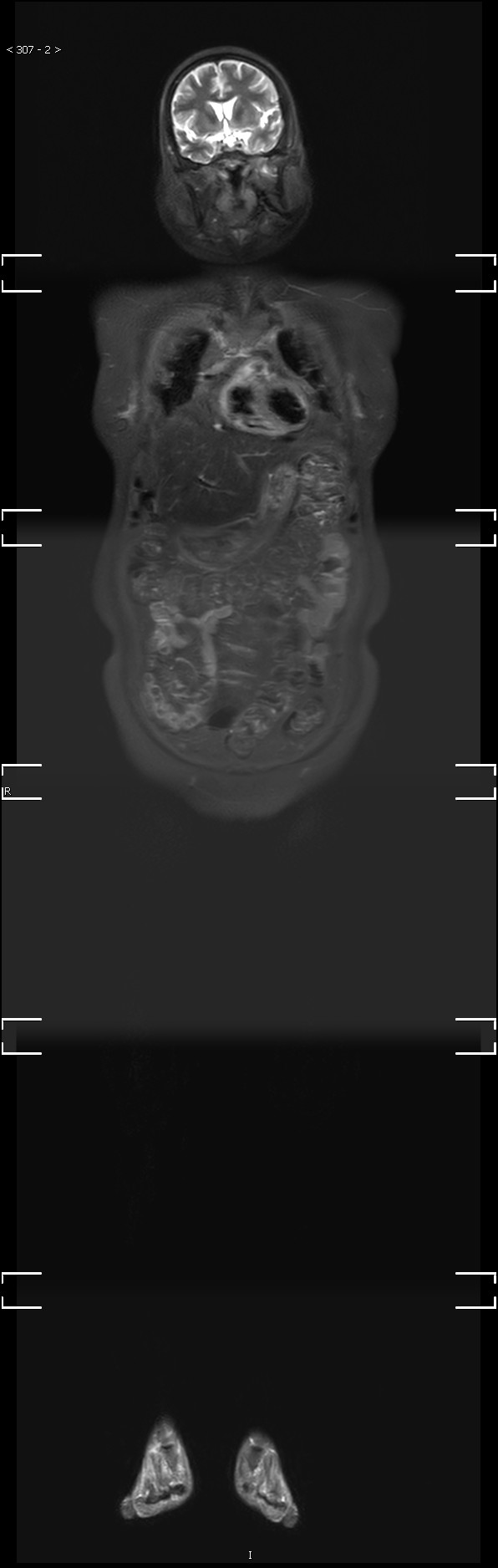

Supplement: S3 Fig — (ZIP) [file pone.0181069.s003.zip › S3/02.jpg]

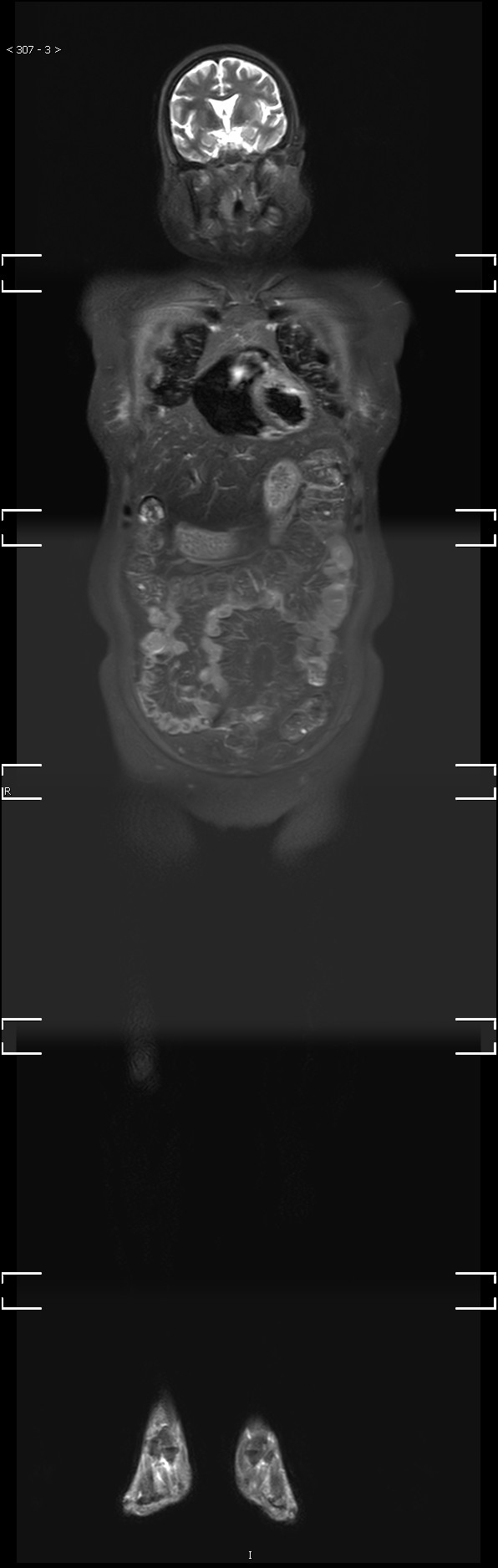

Supplement: S3 Fig — (ZIP) [file pone.0181069.s003.zip › S3/03.jpg]

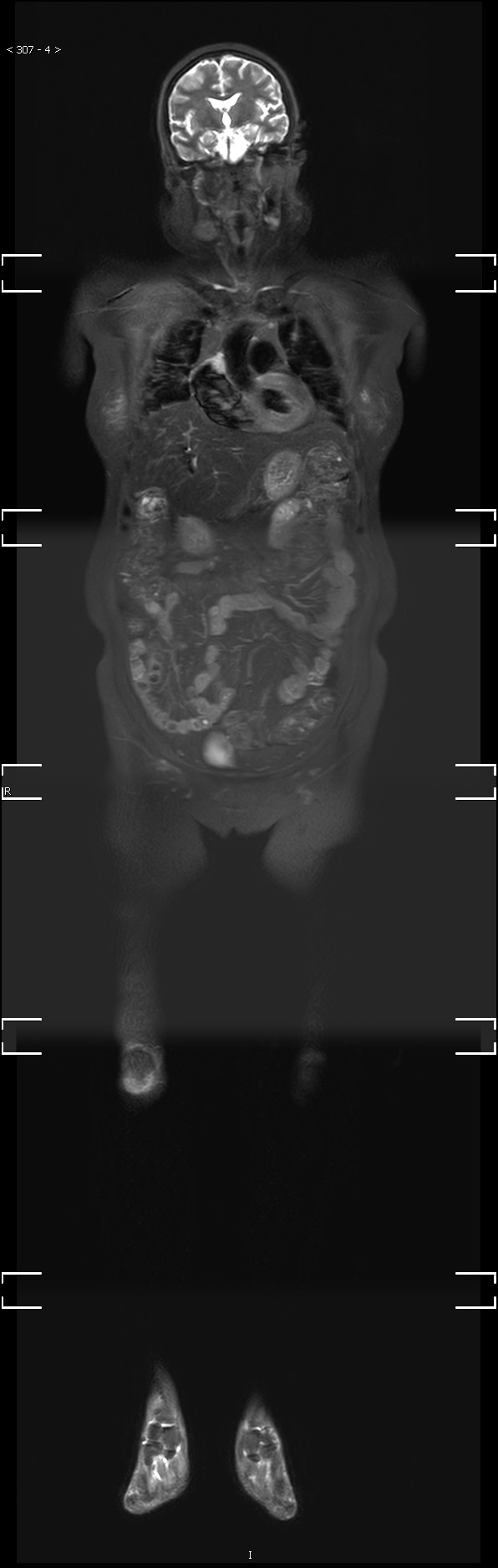

Supplement: S3 Fig — (ZIP) [file pone.0181069.s003.zip › S3/04.jpg]

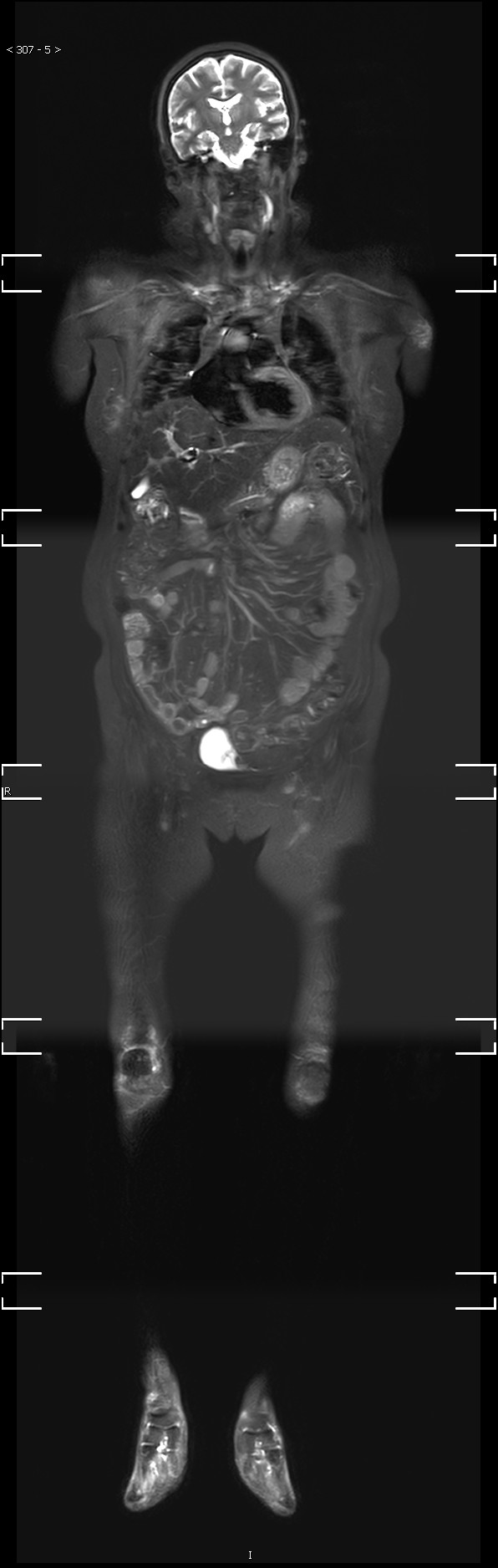

Supplement: S3 Fig — (ZIP) [file pone.0181069.s003.zip › S3/05.jpg]

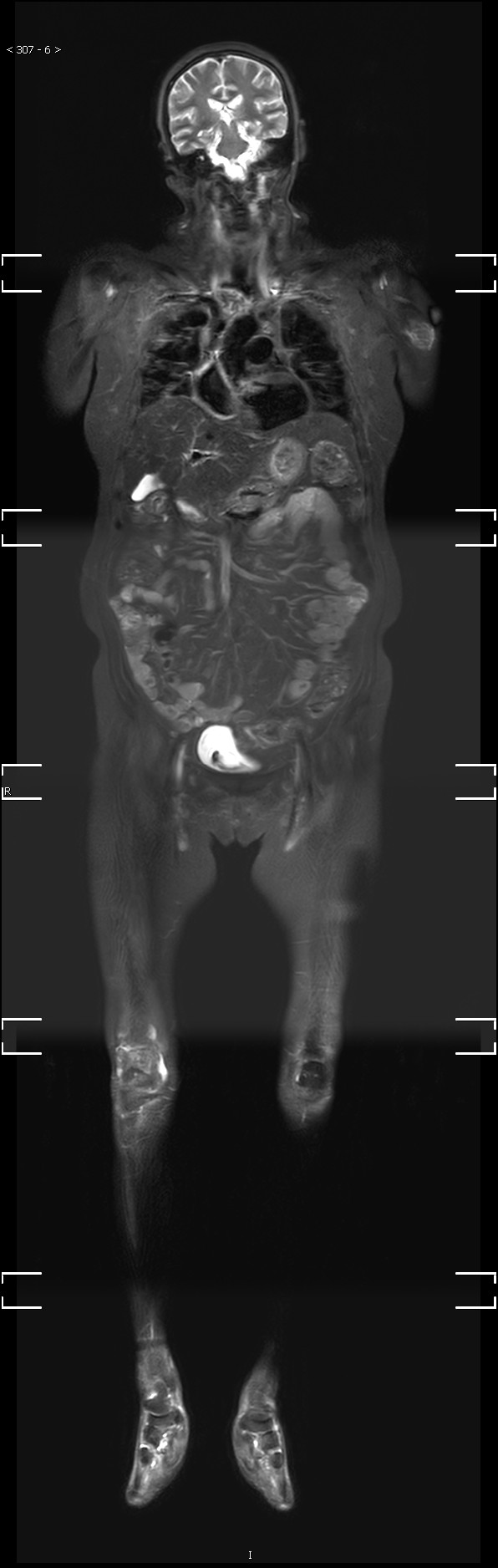

Supplement: S3 Fig — (ZIP) [file pone.0181069.s003.zip › S3/06.jpg]

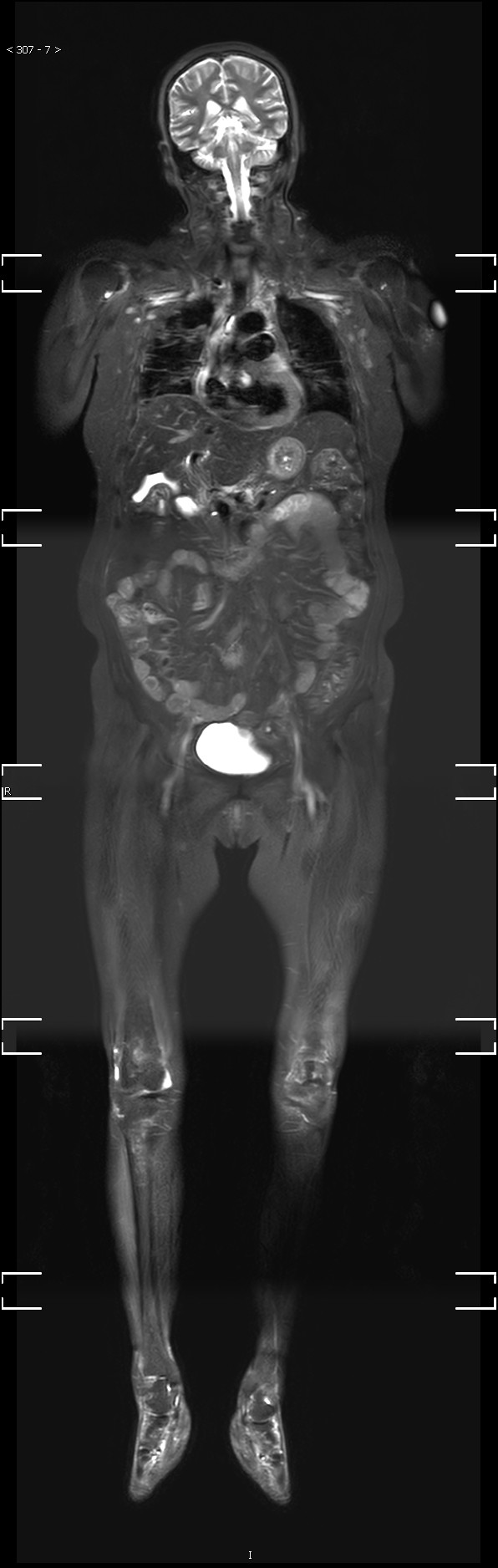

Supplement: S3 Fig — (ZIP) [file pone.0181069.s003.zip › S3/07.jpg]

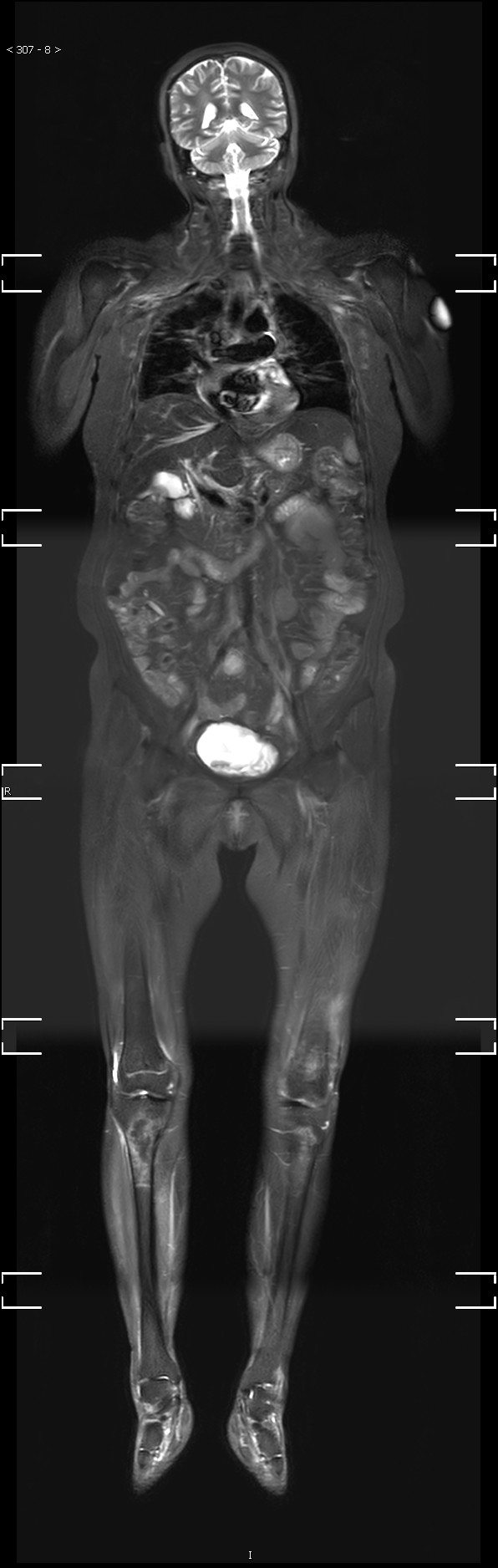

Supplement: S3 Fig — (ZIP) [file pone.0181069.s003.zip › S3/08.jpg]

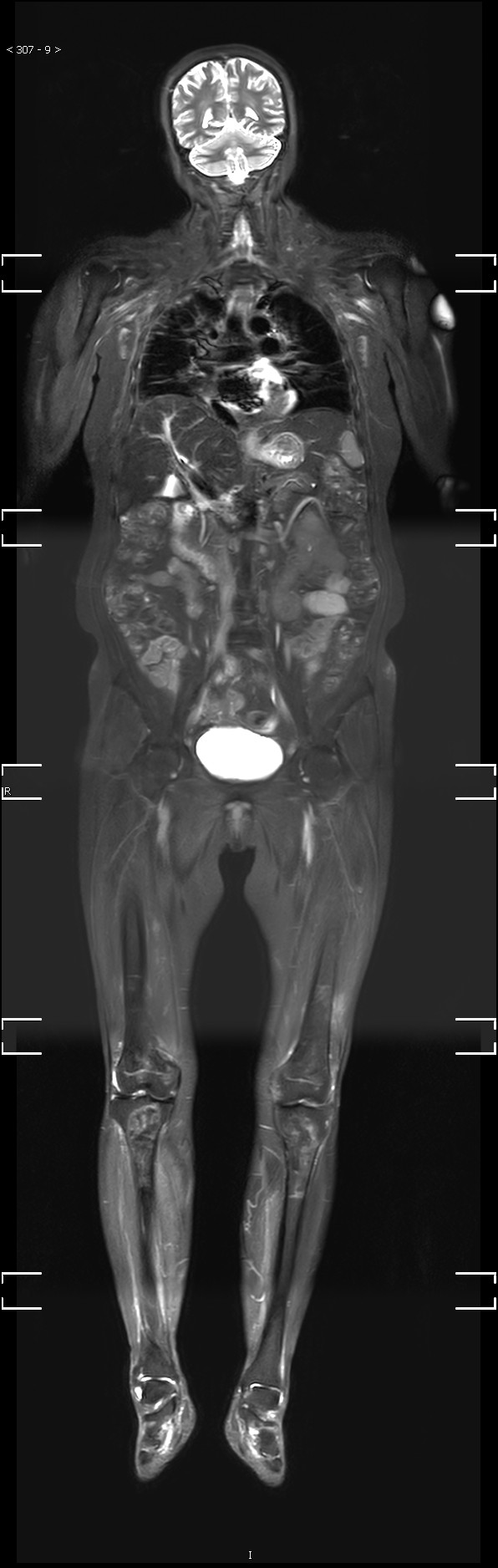

Supplement: S3 Fig — (ZIP) [file pone.0181069.s003.zip › S3/09.jpg]

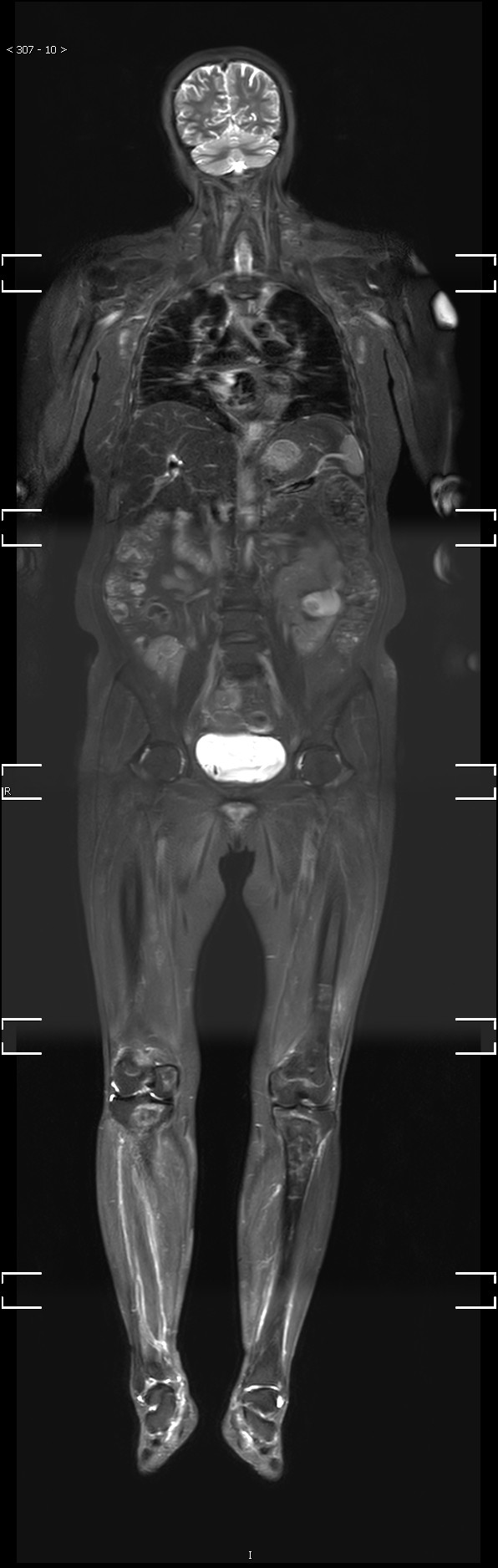

Supplement: S3 Fig — (ZIP) [file pone.0181069.s003.zip › S3/10.jpg]

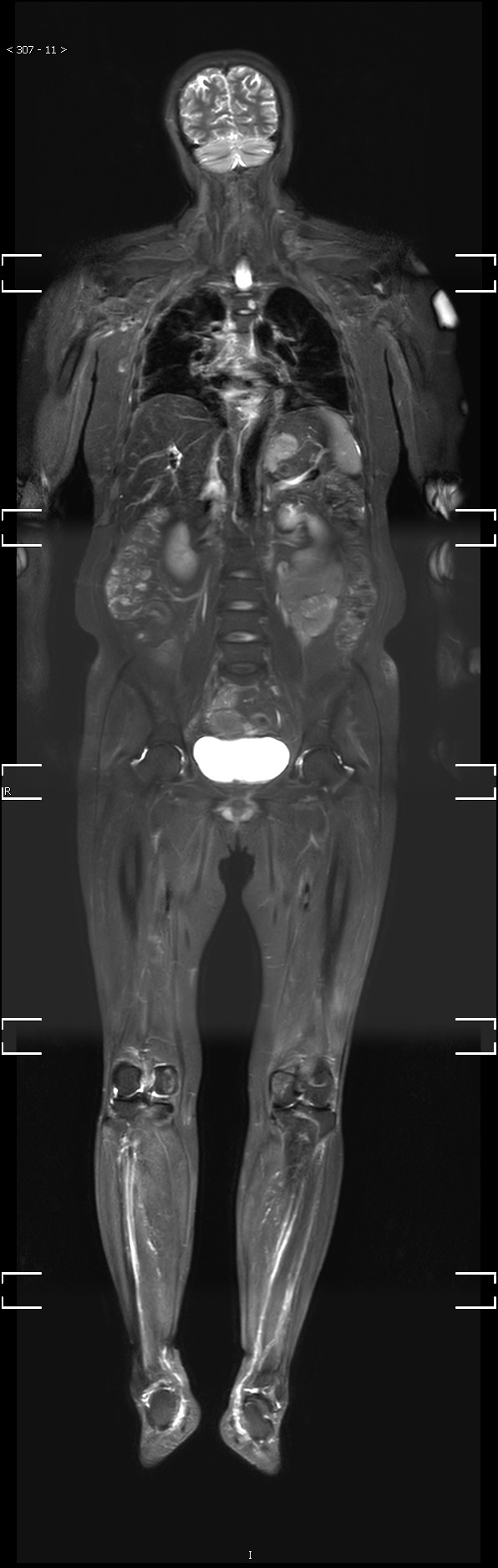

Supplement: S3 Fig — (ZIP) [file pone.0181069.s003.zip › S3/11.jpg]

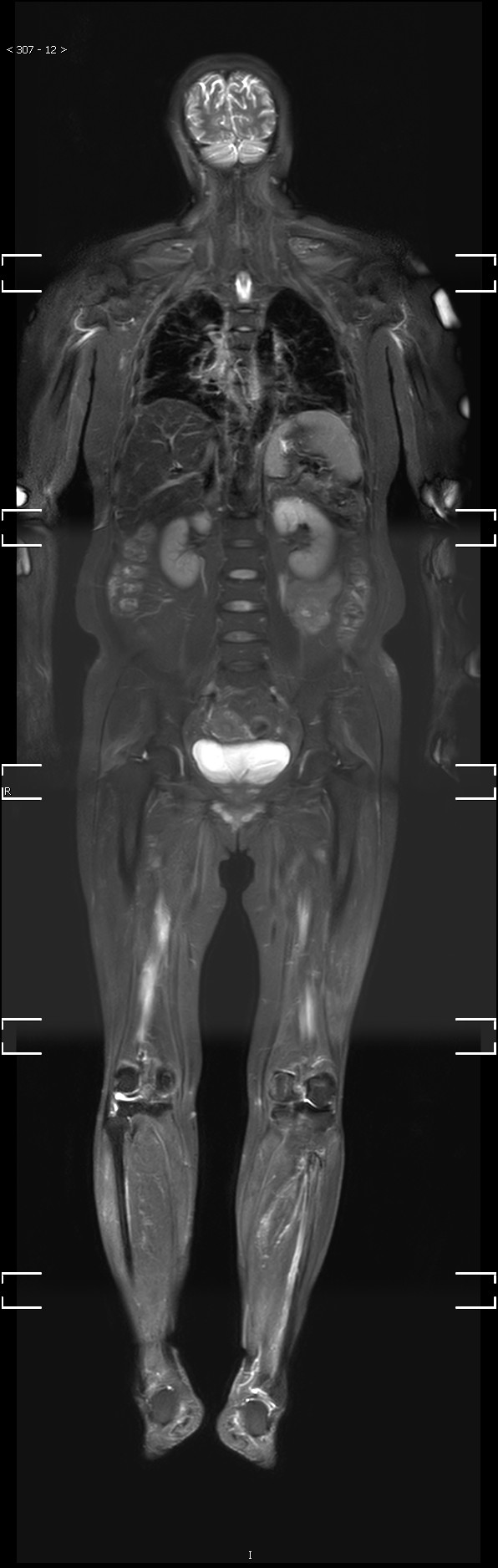

Supplement: S3 Fig — (ZIP) [file pone.0181069.s003.zip › S3/12.jpg]

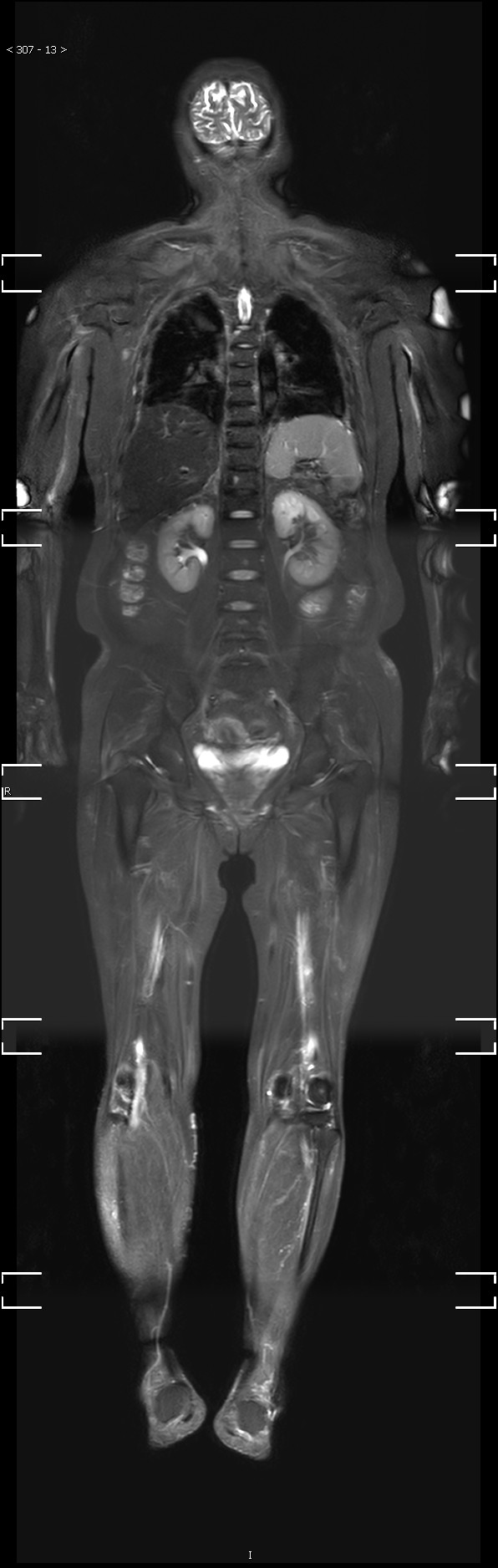

Supplement: S3 Fig — (ZIP) [file pone.0181069.s003.zip › S3/13.jpg]

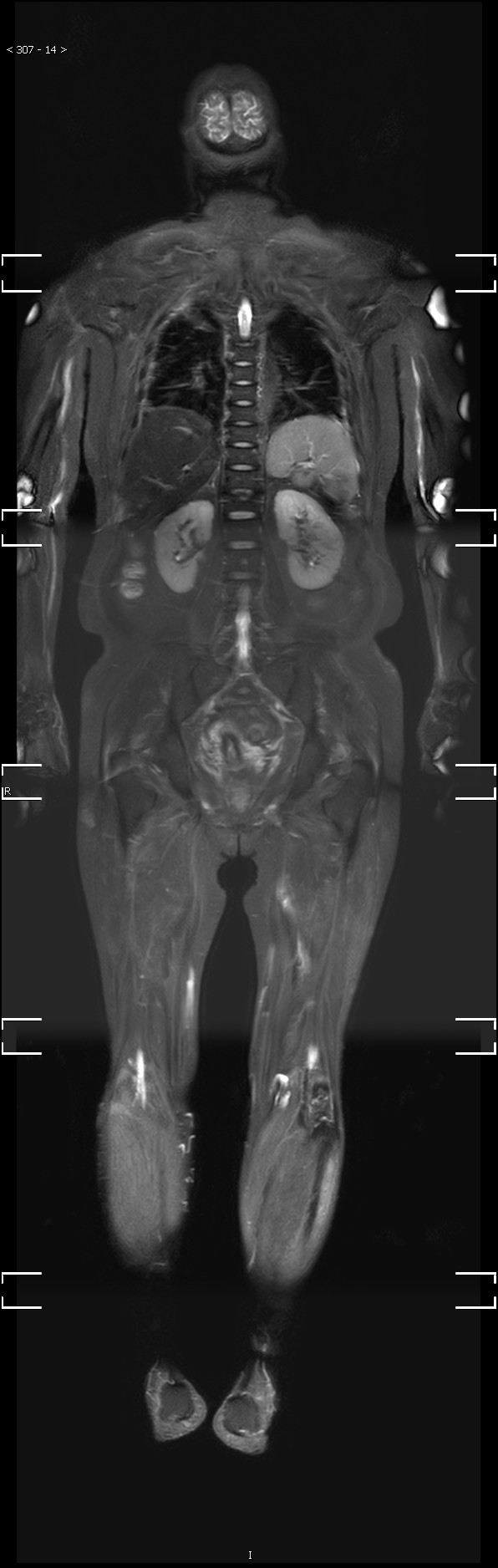

Supplement: S3 Fig — (ZIP) [file pone.0181069.s003.zip › S3/14.jpg]

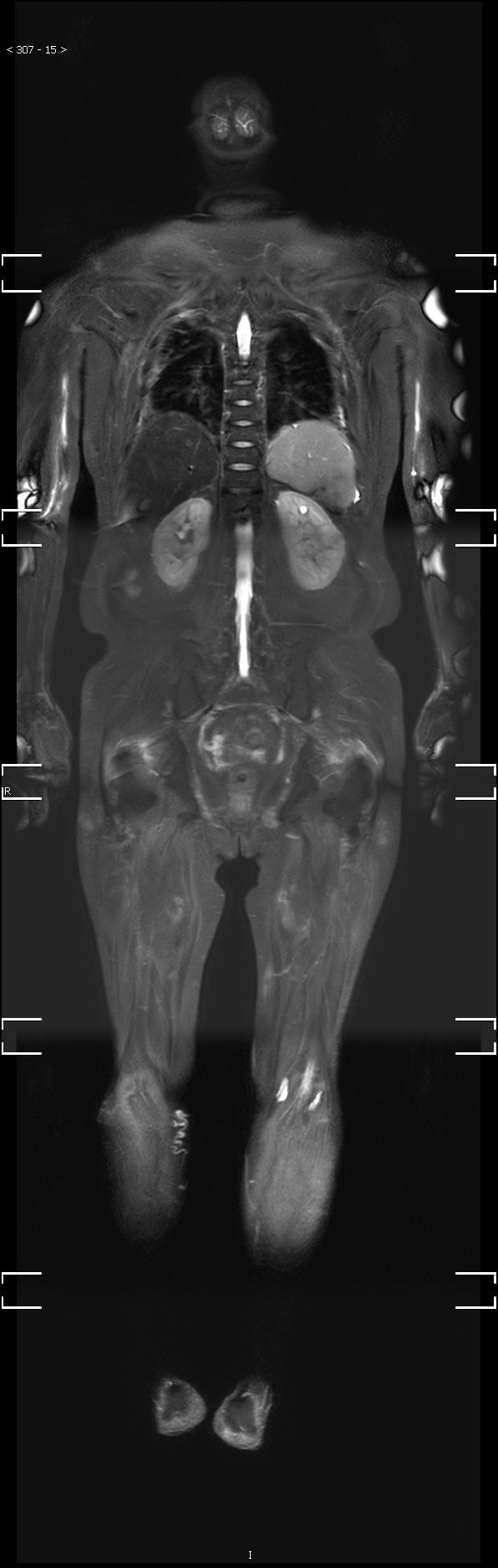

Supplement: S3 Fig — (ZIP) [file pone.0181069.s003.zip › S3/15.jpg]

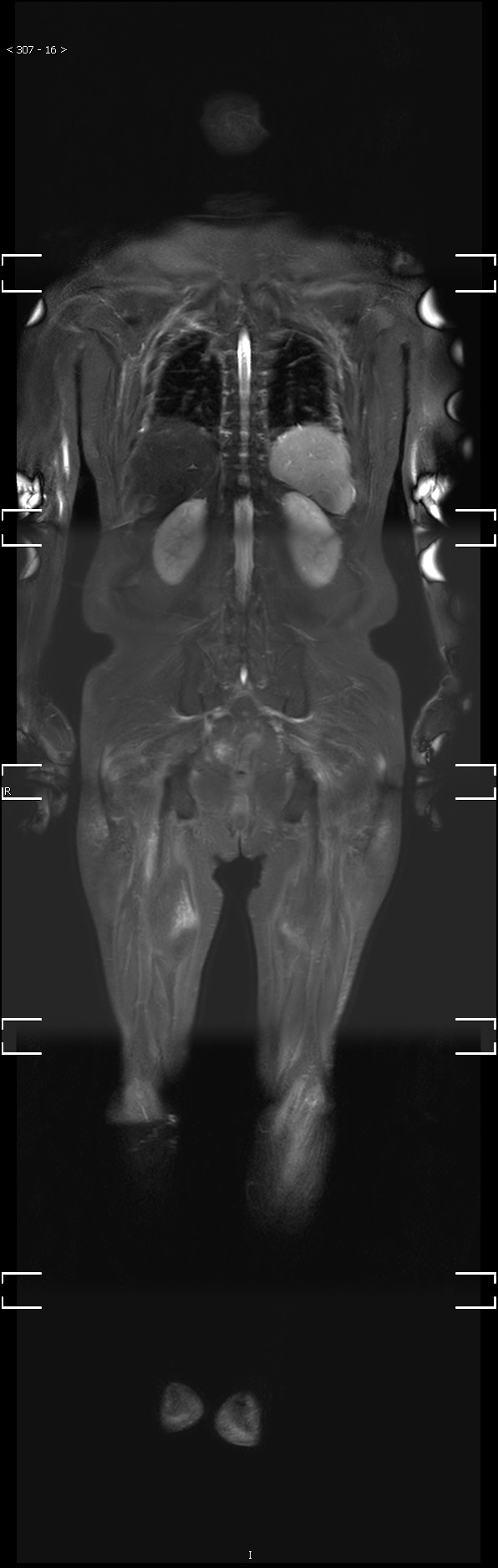

Supplement: S3 Fig — (ZIP) [file pone.0181069.s003.zip › S3/16.jpg]

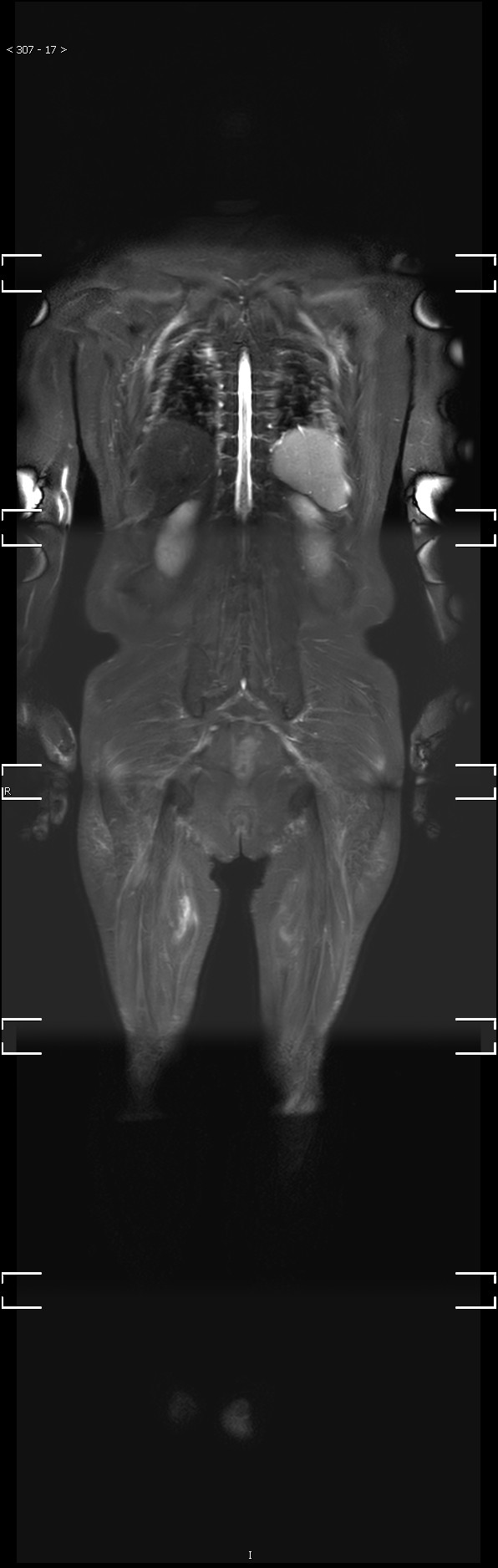

Supplement: S3 Fig — (ZIP) [file pone.0181069.s003.zip › S3/17.jpg]

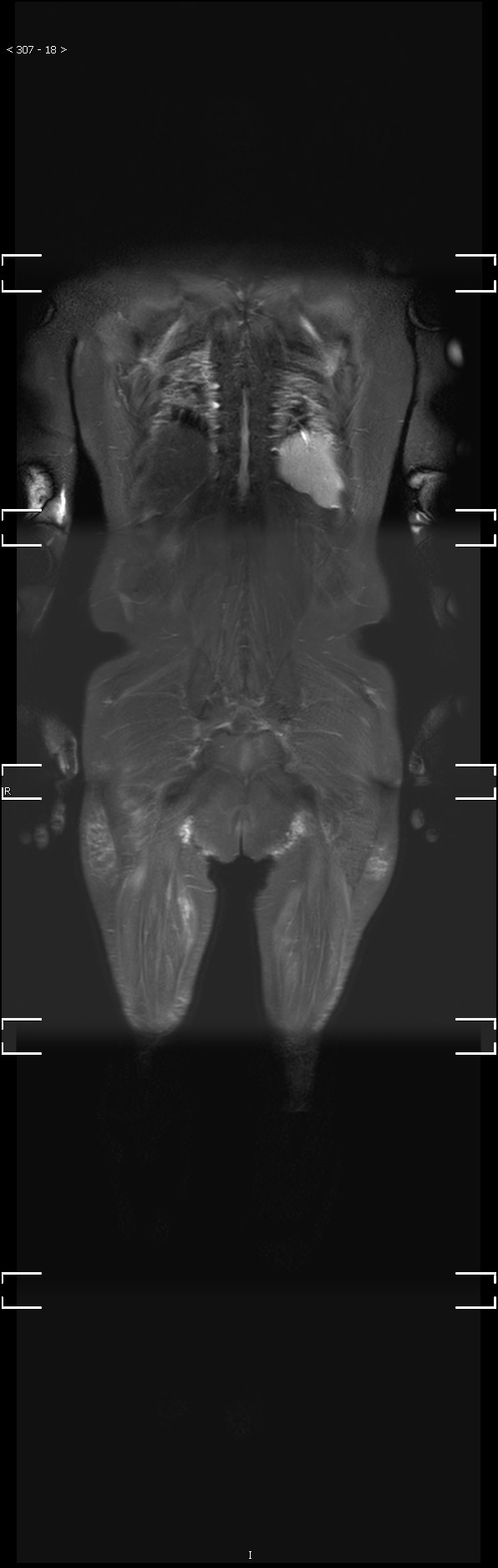

Supplement: S3 Fig — (ZIP) [file pone.0181069.s003.zip › S3/18.jpg]

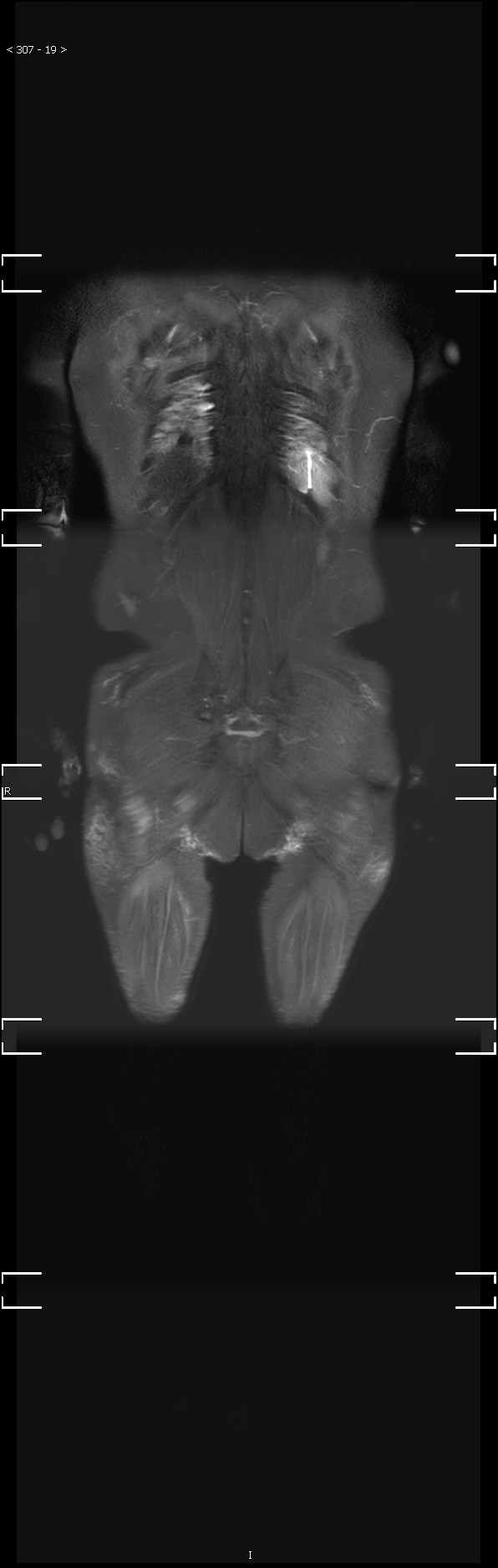

Supplement: S3 Fig — (ZIP) [file pone.0181069.s003.zip › S3/19.jpg]

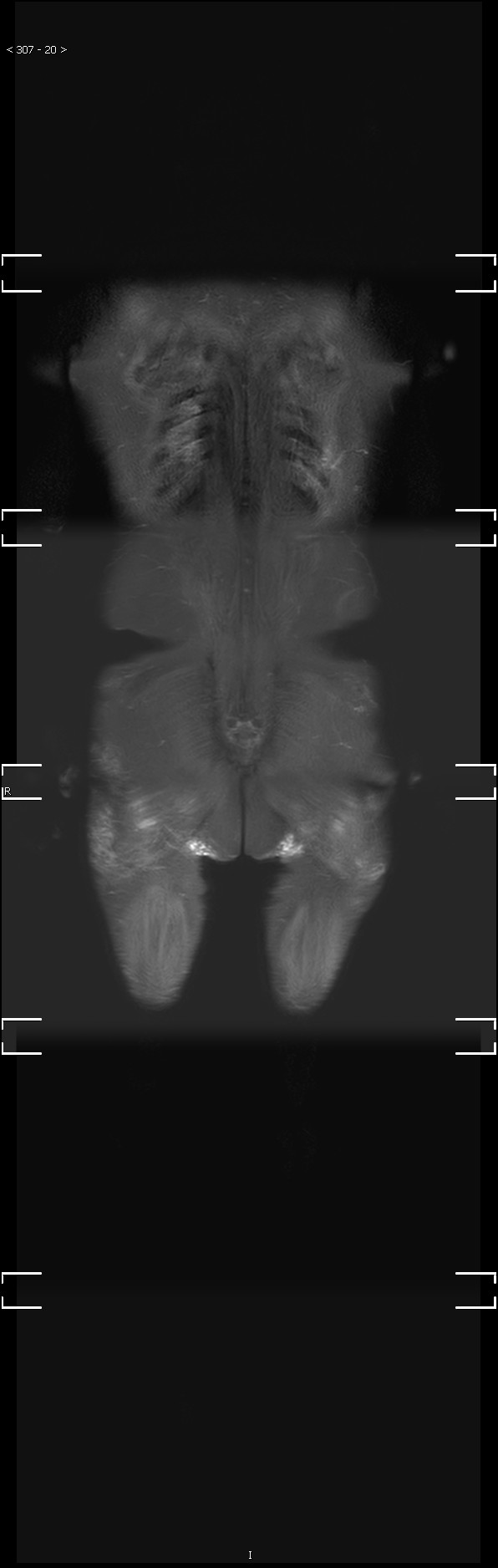

Supplement: S3 Fig — (ZIP) [file pone.0181069.s003.zip › S3/20.jpg]

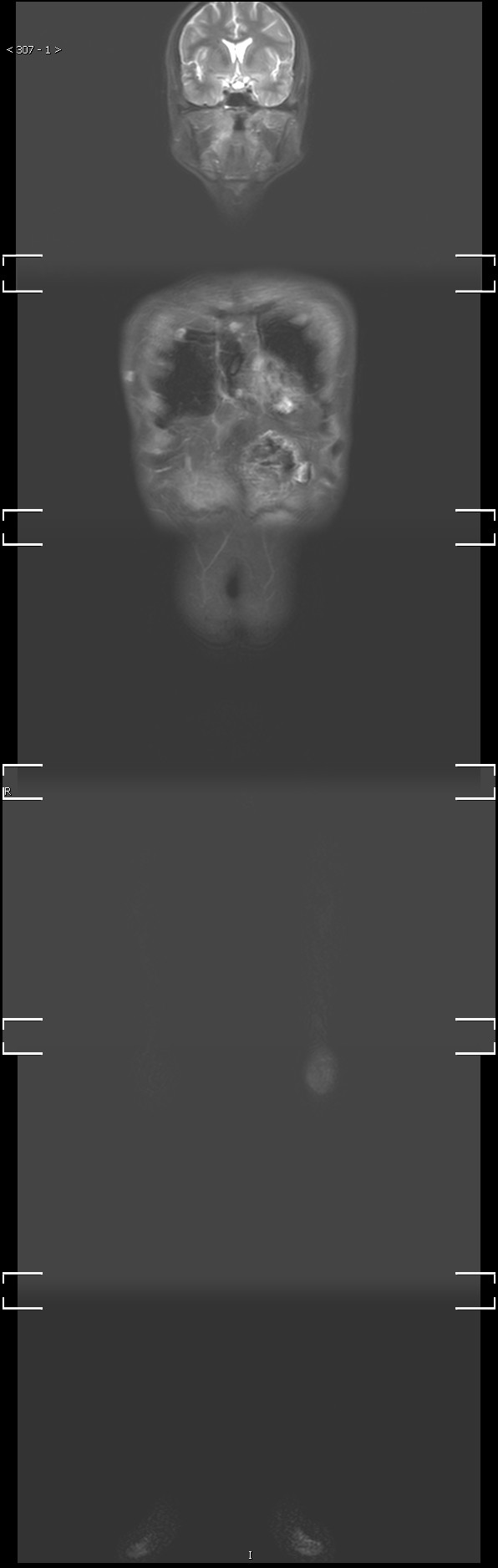

Supplement: S4 Fig — (ZIP) [file pone.0181069.s004.zip › S4/01.jpg]

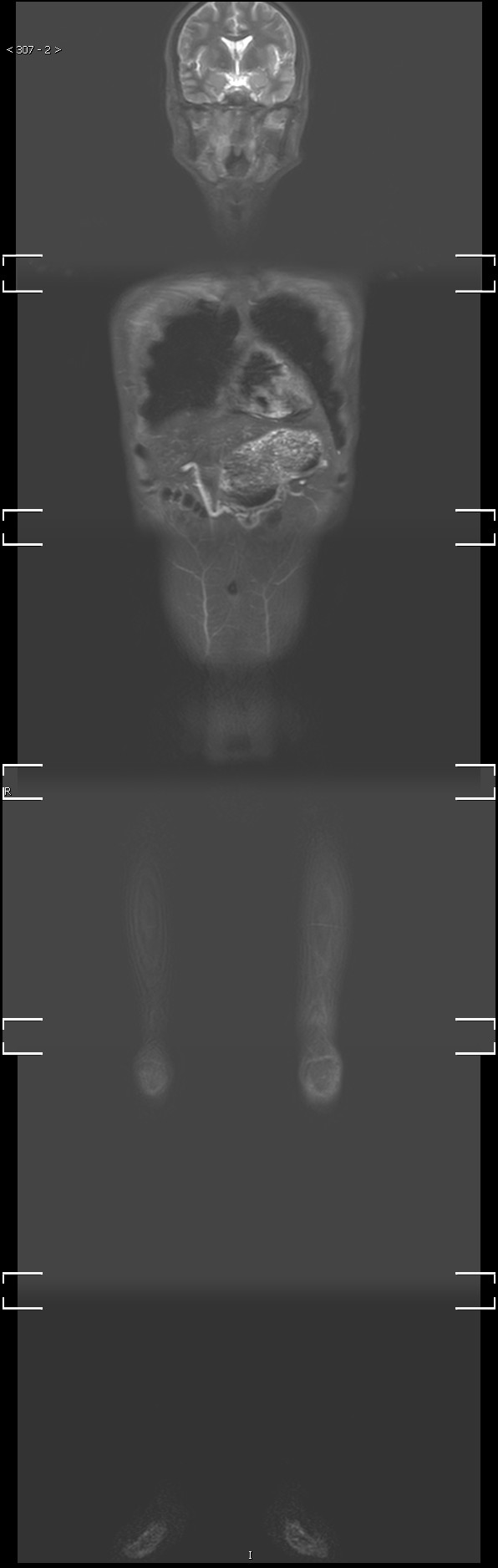

Supplement: S4 Fig — (ZIP) [file pone.0181069.s004.zip › S4/02.jpg]

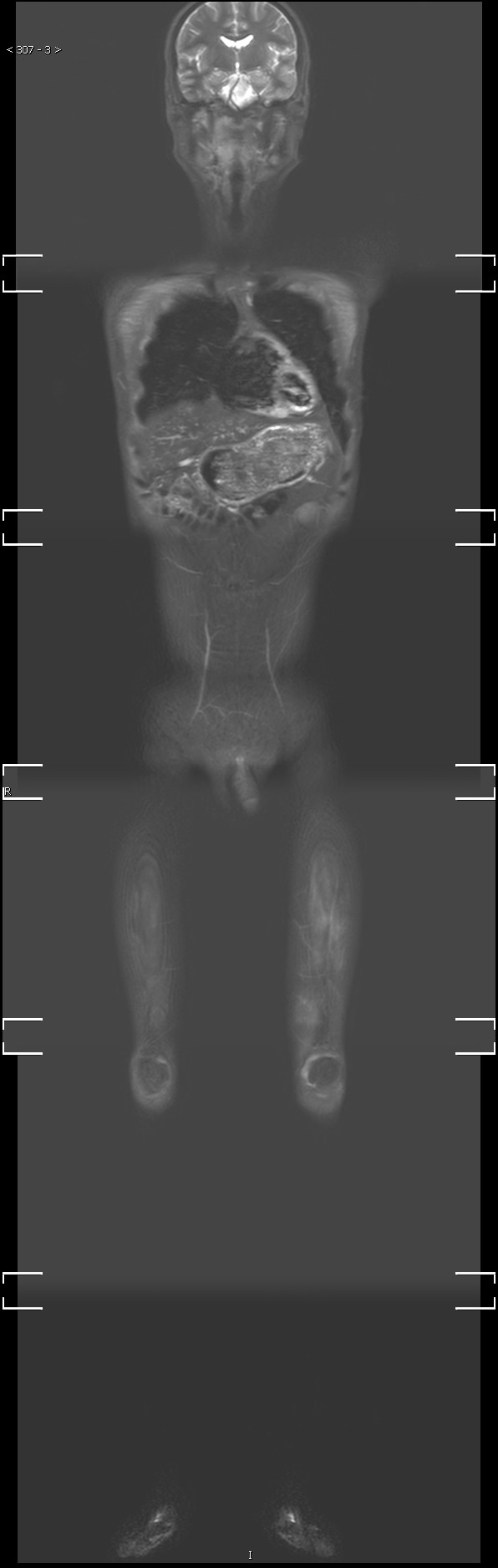

Supplement: S4 Fig — (ZIP) [file pone.0181069.s004.zip › S4/03.jpg]

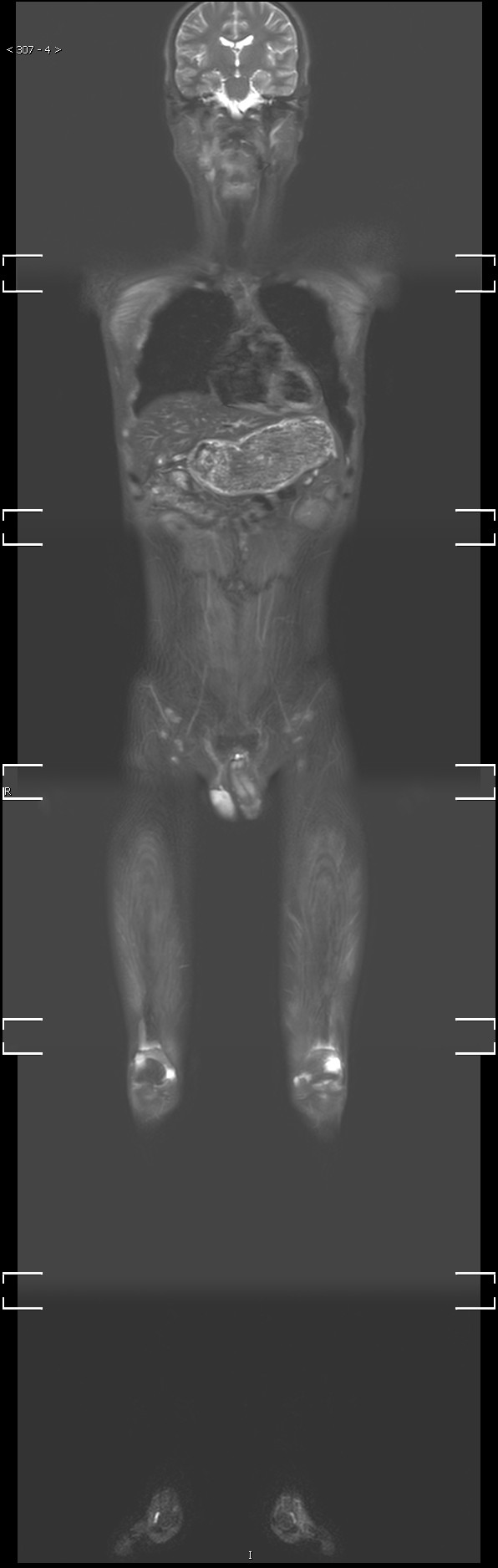

Supplement: S4 Fig — (ZIP) [file pone.0181069.s004.zip › S4/04.jpg]

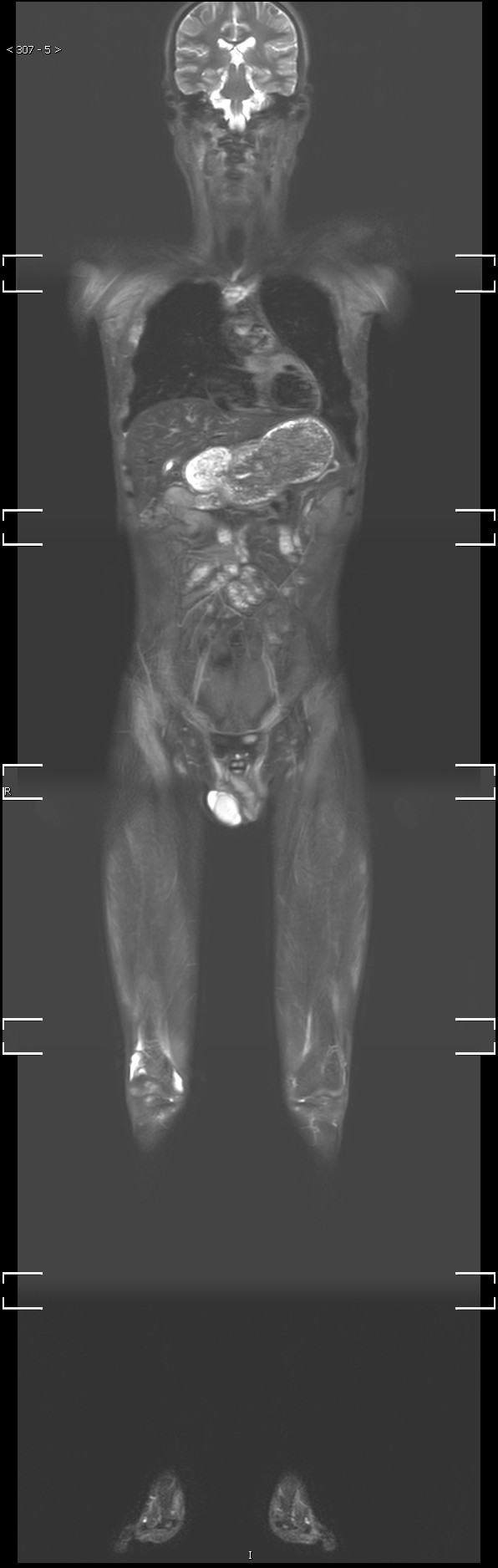

Supplement: S4 Fig — (ZIP) [file pone.0181069.s004.zip › S4/05.jpg]

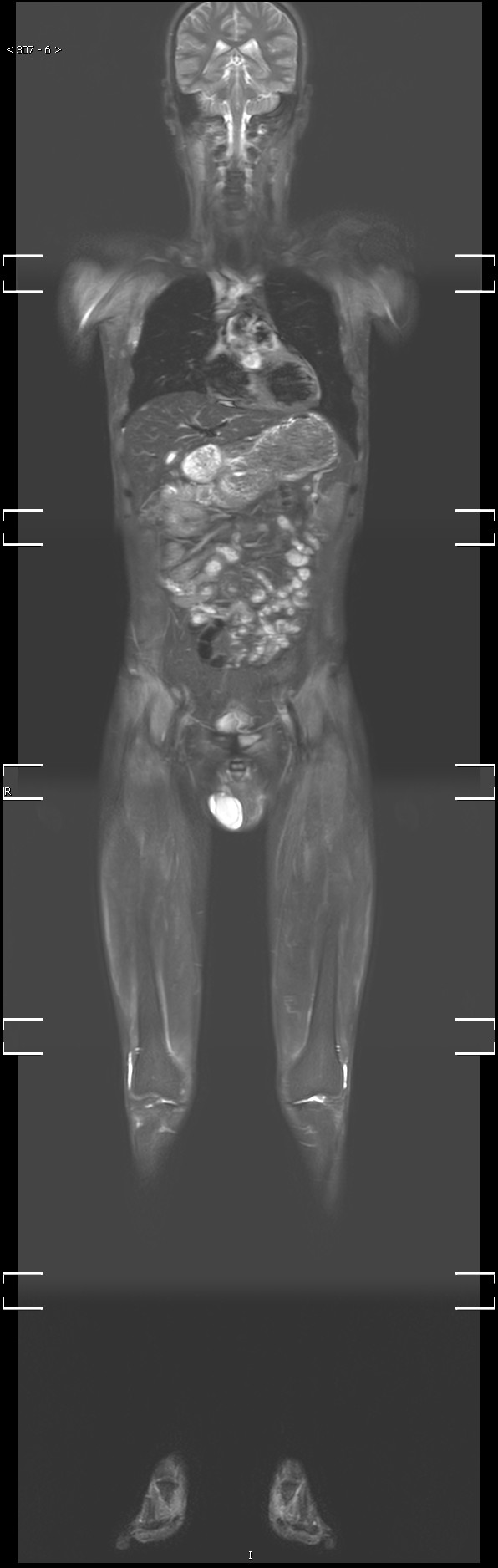

Supplement: S4 Fig — (ZIP) [file pone.0181069.s004.zip › S4/06.jpg]

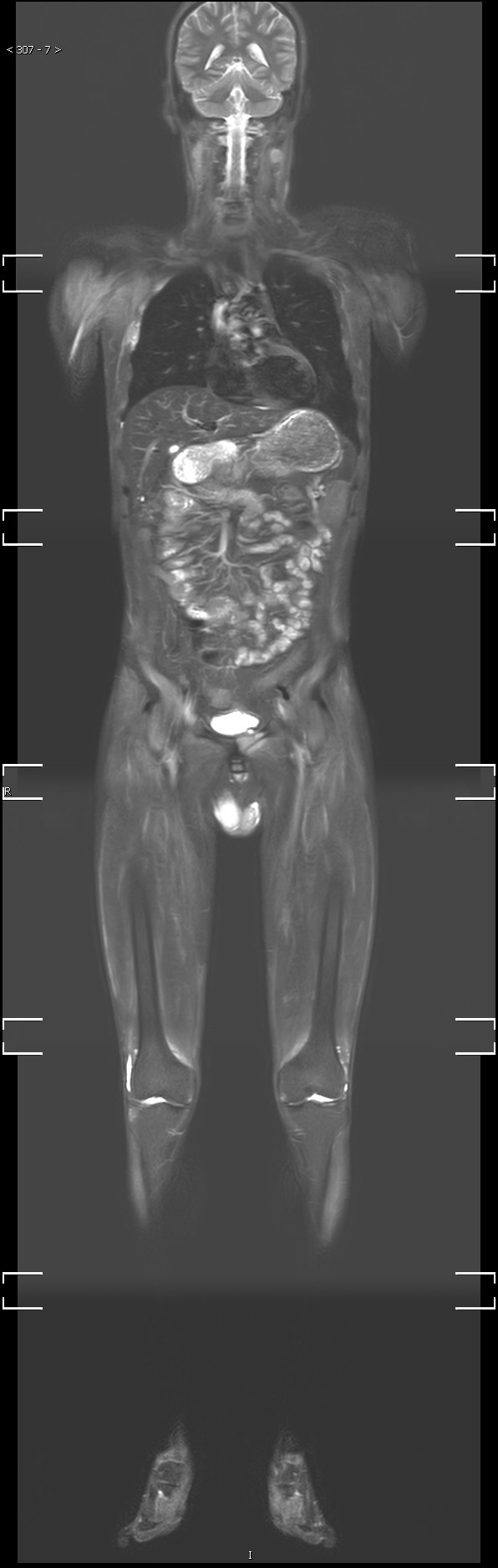

Supplement: S4 Fig — (ZIP) [file pone.0181069.s004.zip › S4/07.jpg]

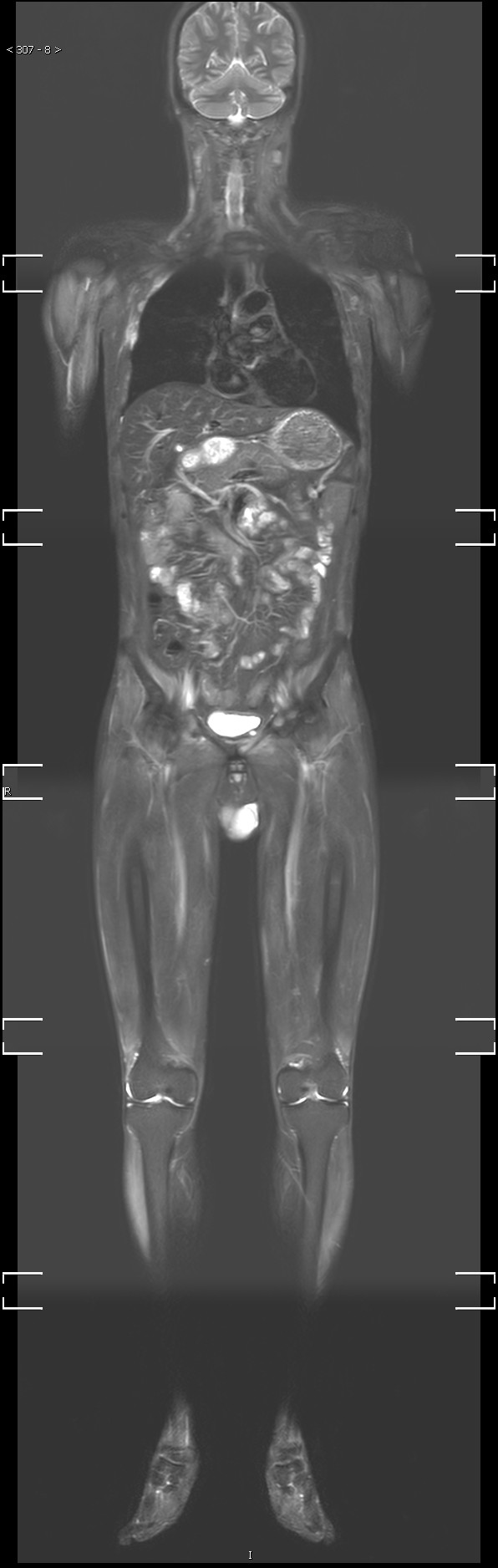

Supplement: S4 Fig — (ZIP) [file pone.0181069.s004.zip › S4/08.jpg]

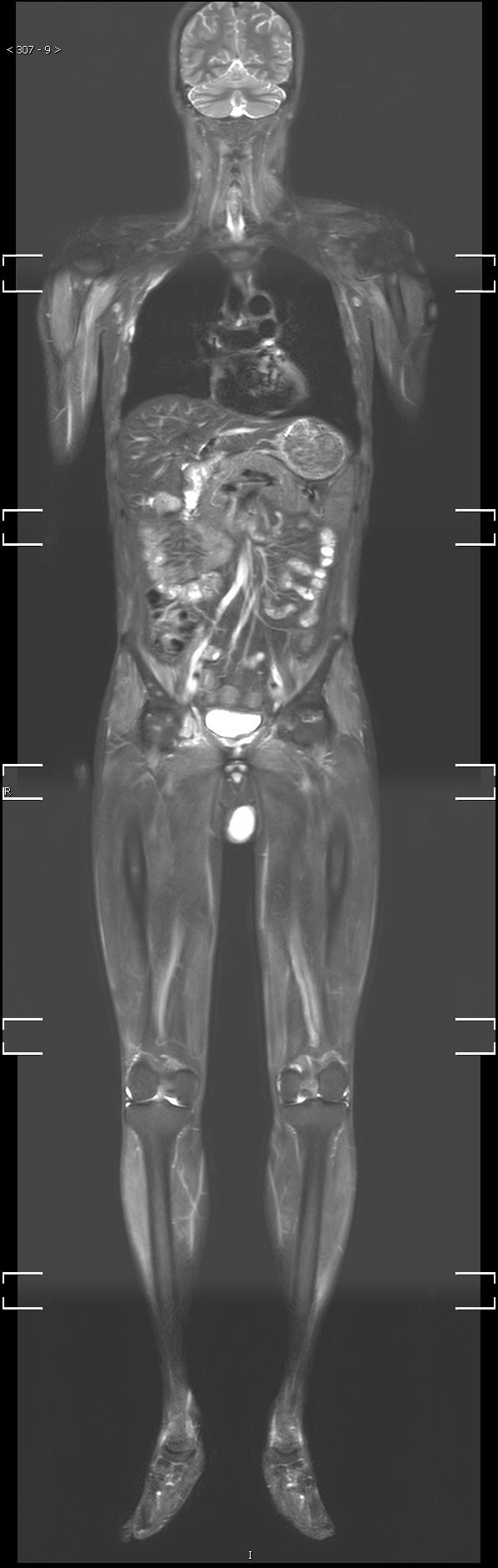

Supplement: S4 Fig — (ZIP) [file pone.0181069.s004.zip › S4/09.jpg]

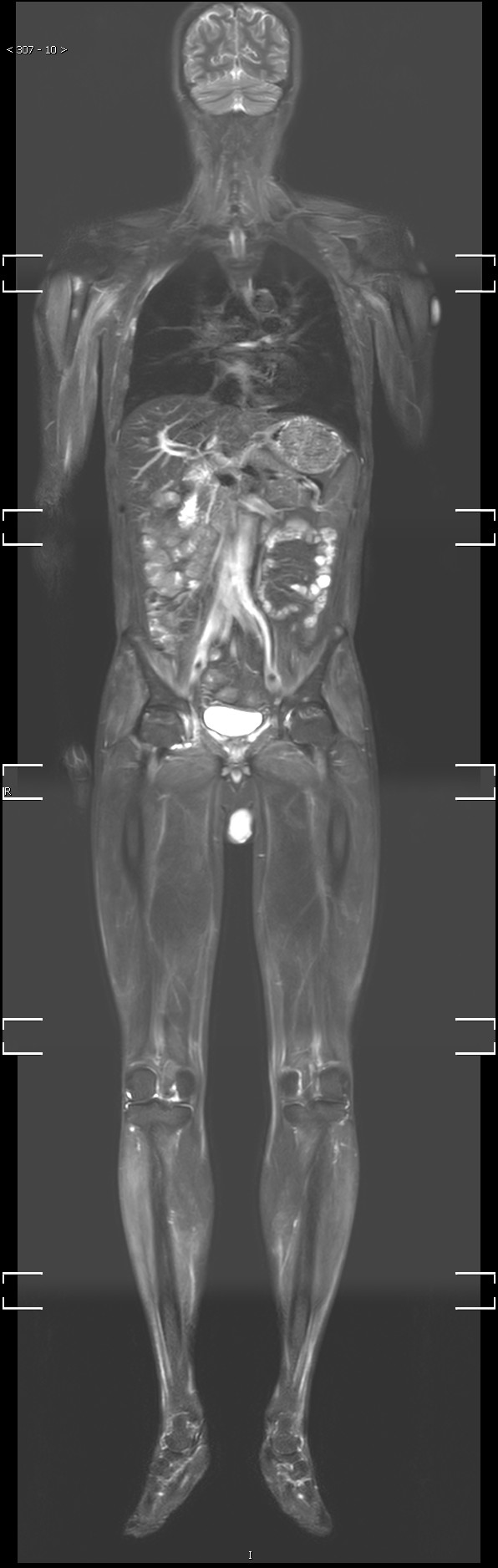

Supplement: S4 Fig — (ZIP) [file pone.0181069.s004.zip › S4/10.jpg]

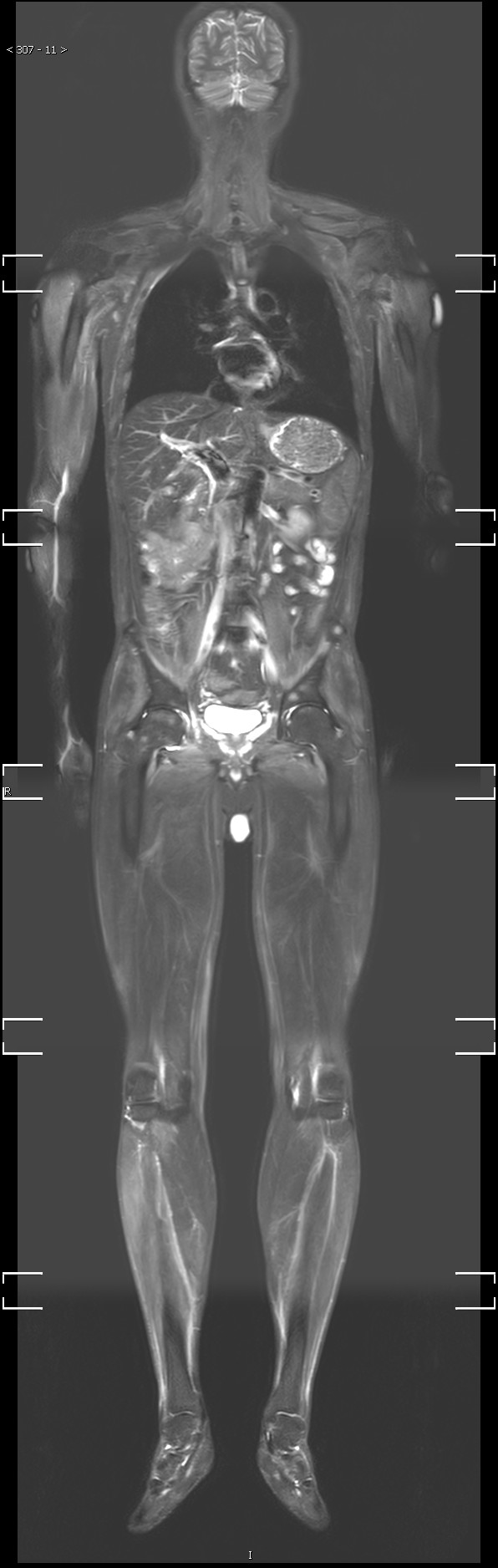

Supplement: S4 Fig — (ZIP) [file pone.0181069.s004.zip › S4/11.jpg]

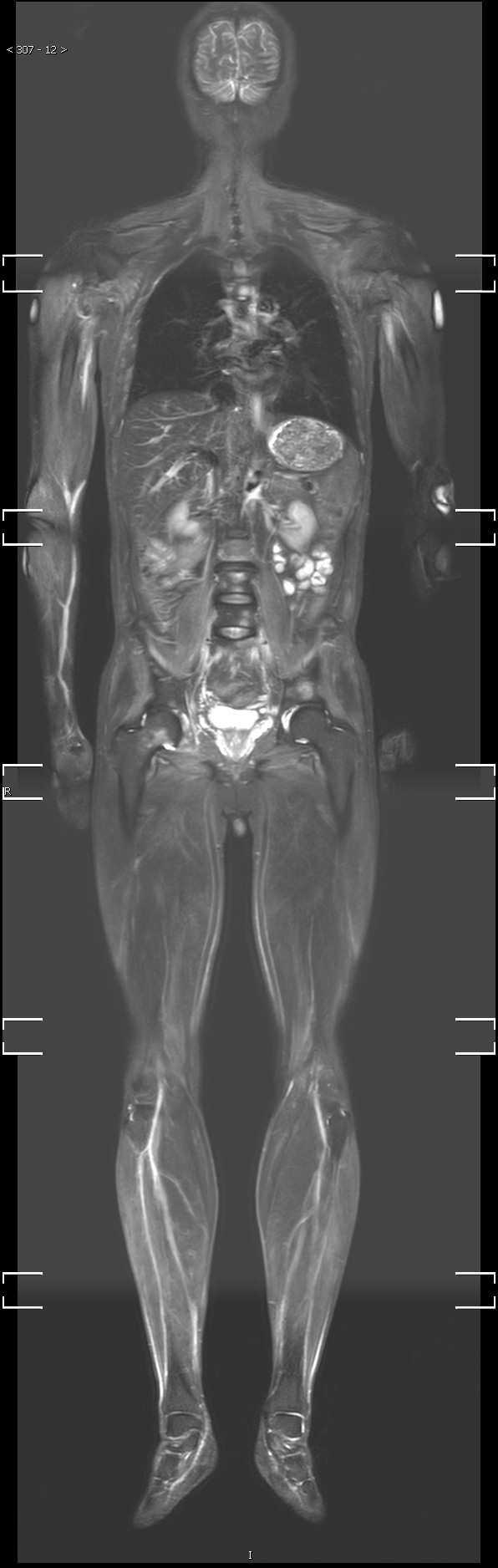

Supplement: S4 Fig — (ZIP) [file pone.0181069.s004.zip › S4/12.jpg]

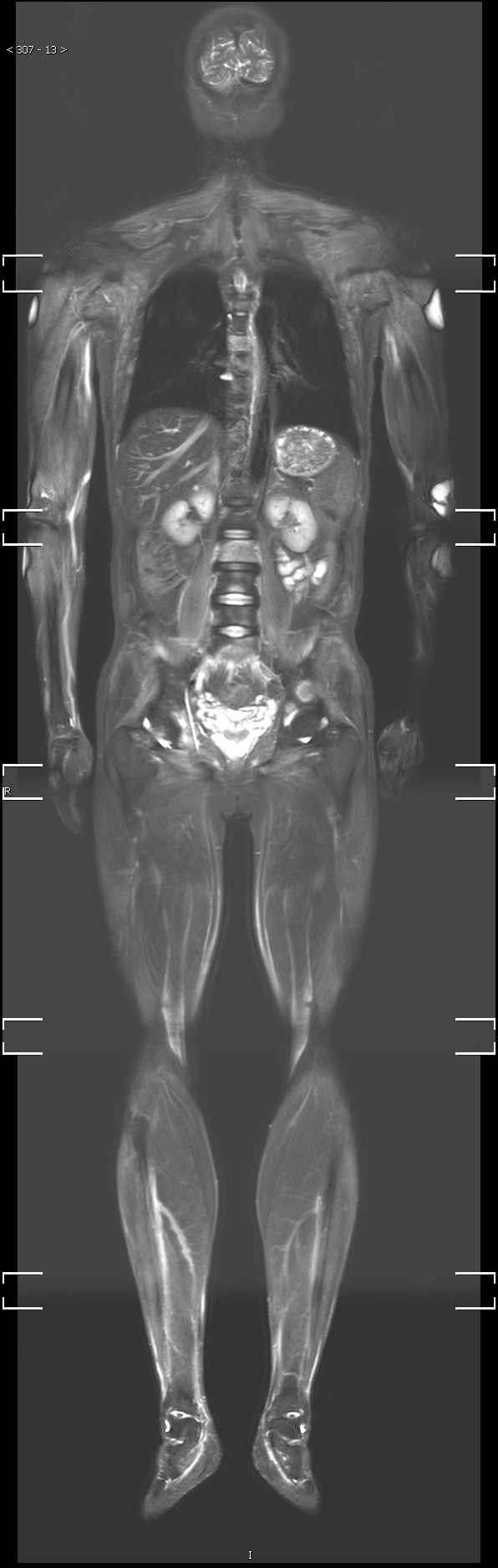

Supplement: S4 Fig — (ZIP) [file pone.0181069.s004.zip › S4/13.jpg]

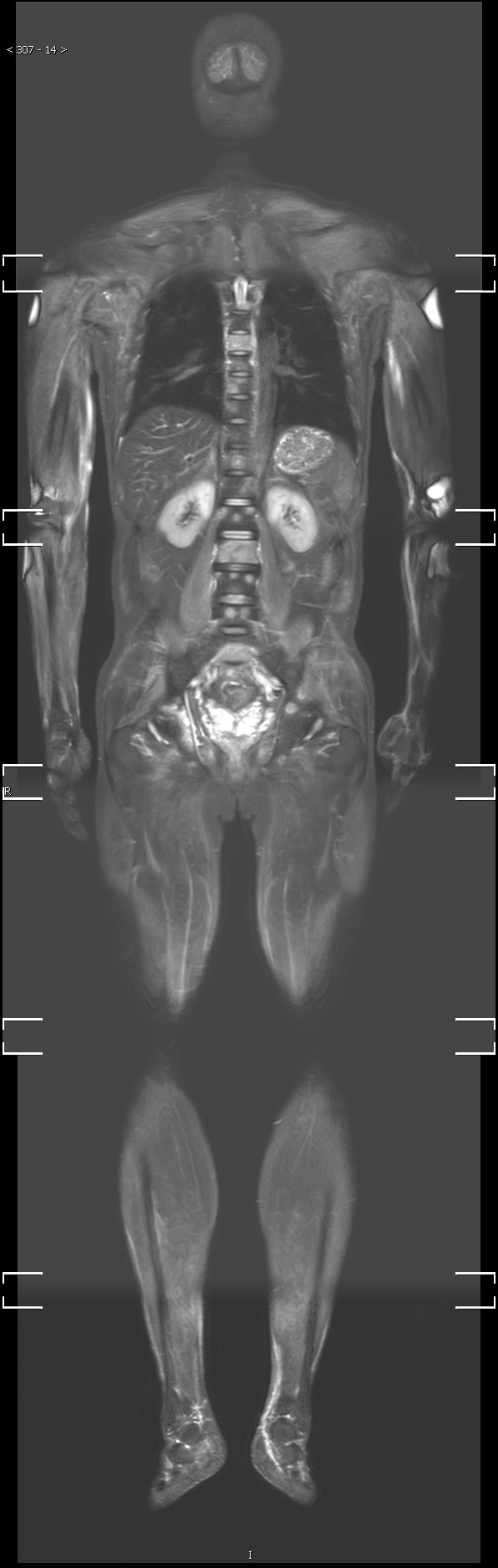

Supplement: S4 Fig — (ZIP) [file pone.0181069.s004.zip › S4/14.jpg]

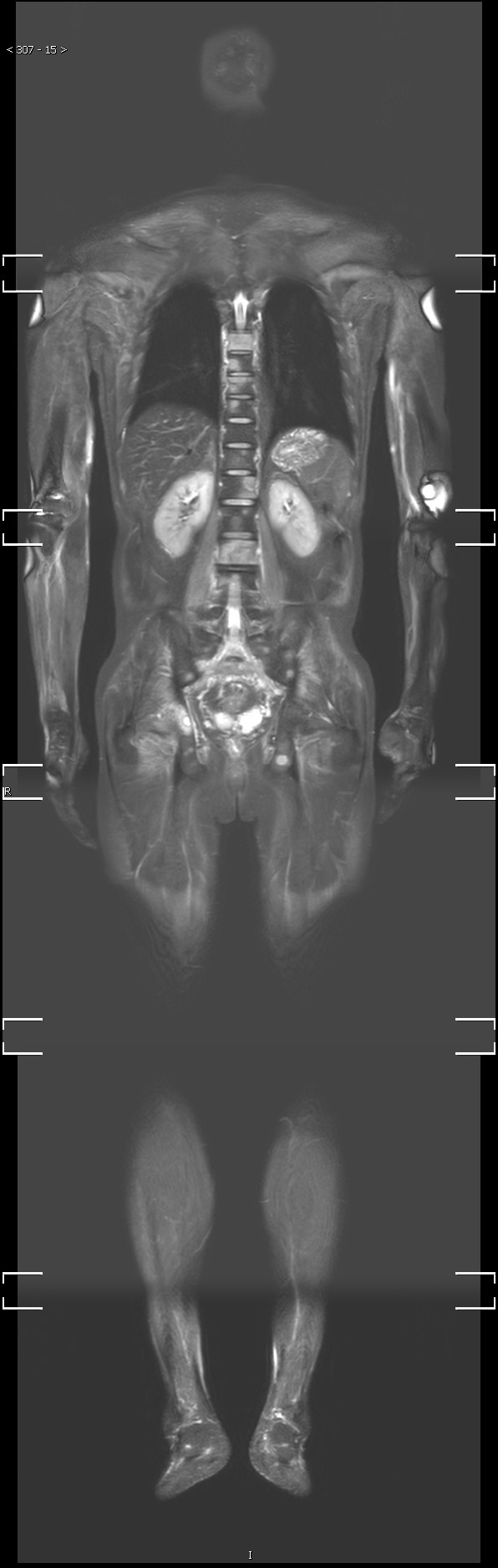

Supplement: S4 Fig — (ZIP) [file pone.0181069.s004.zip › S4/15.jpg]

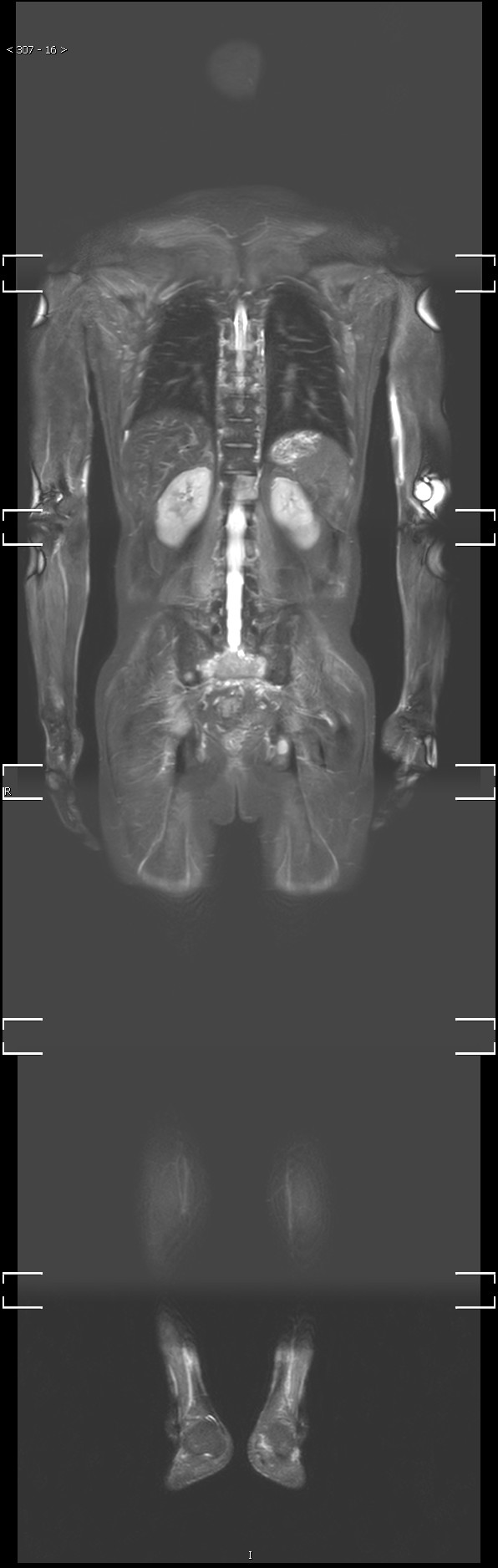

Supplement: S4 Fig — (ZIP) [file pone.0181069.s004.zip › S4/16.jpg]

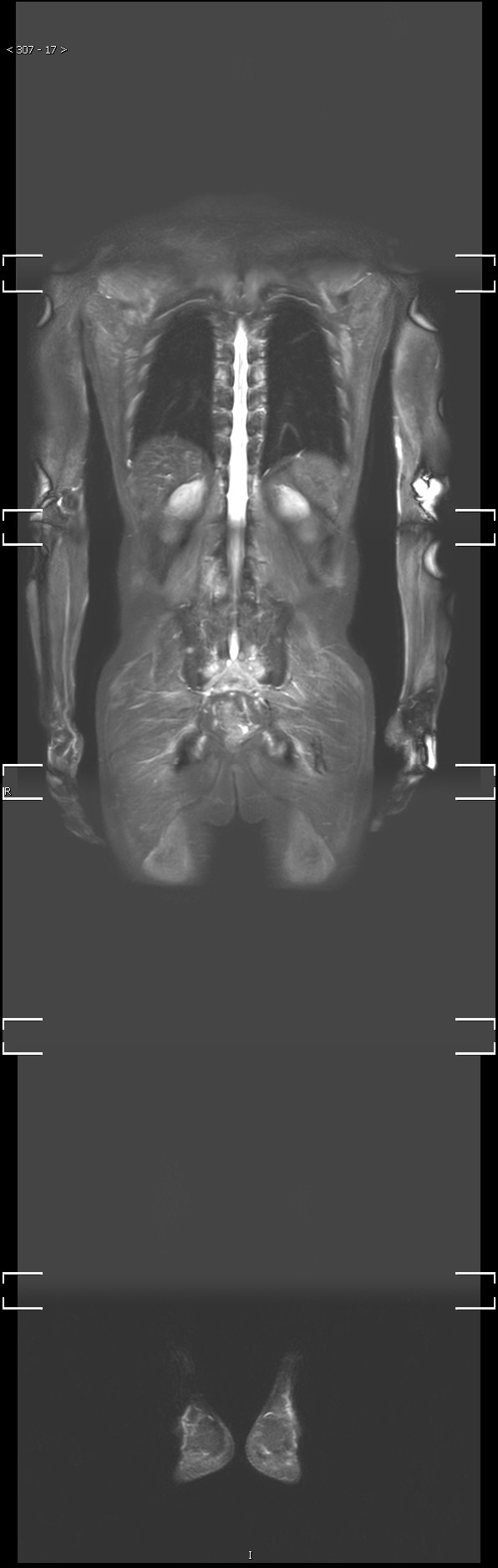

Supplement: S4 Fig — (ZIP) [file pone.0181069.s004.zip › S4/17.jpg]

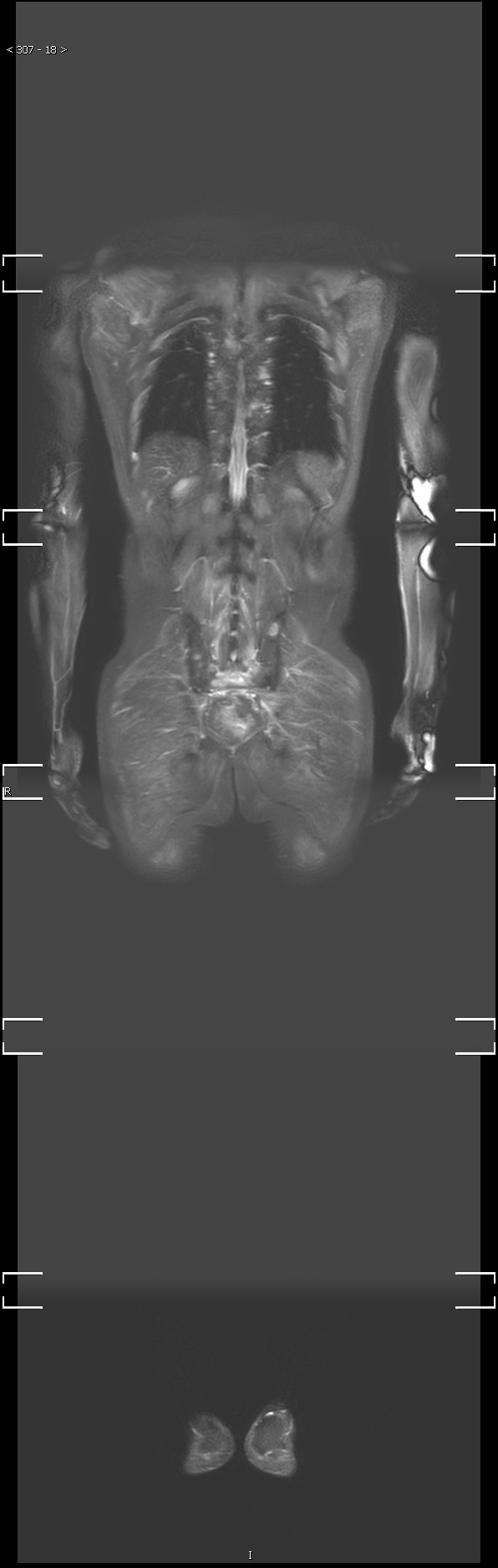

Supplement: S4 Fig — (ZIP) [file pone.0181069.s004.zip › S4/18.jpg]

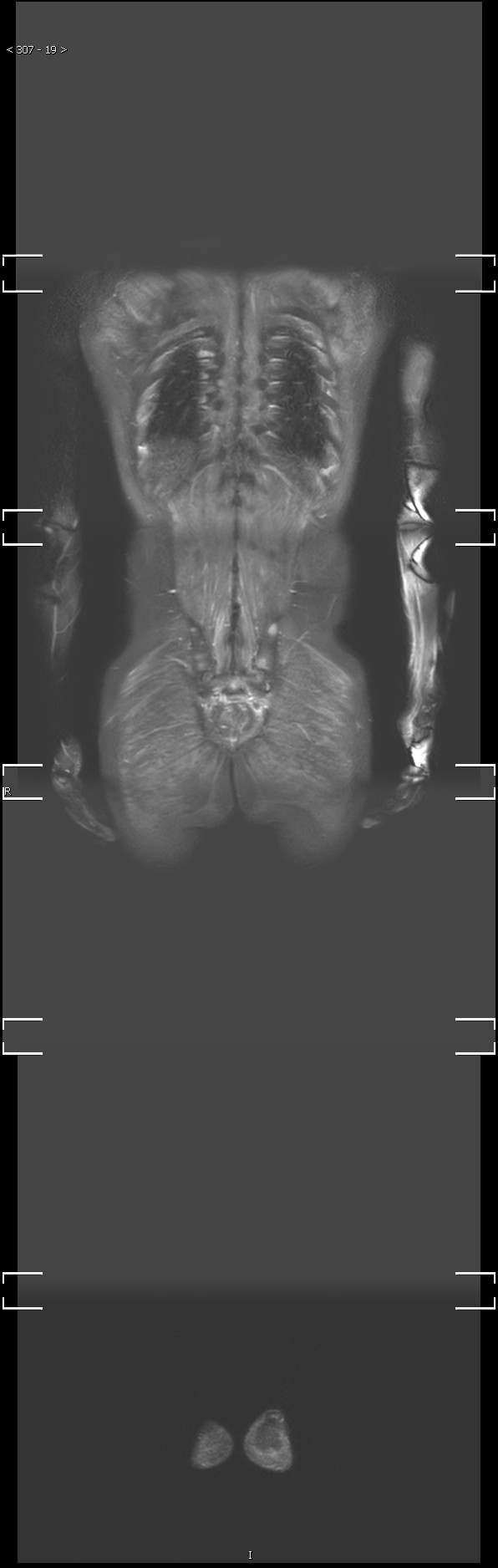

Supplement: S4 Fig — (ZIP) [file pone.0181069.s004.zip › S4/19.jpg]

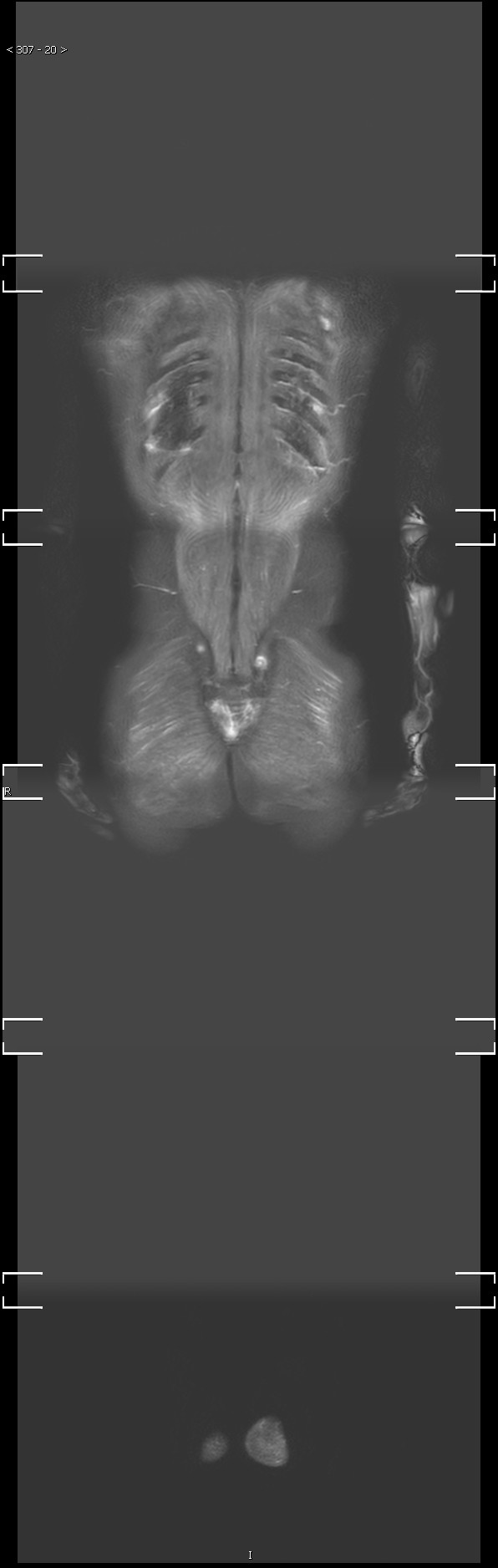

Supplement: S4 Fig — (ZIP) [file pone.0181069.s004.zip › S4/20.jpg]

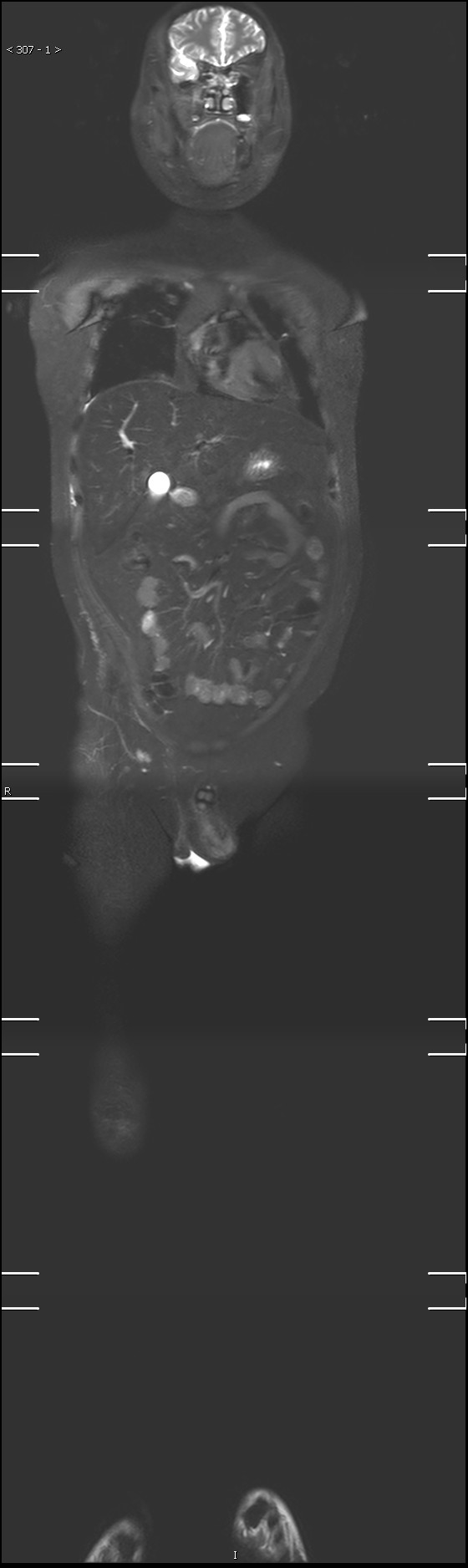

Supplement: S5 Fig — (ZIP) [file pone.0181069.s005.zip › S5/01.jpg]

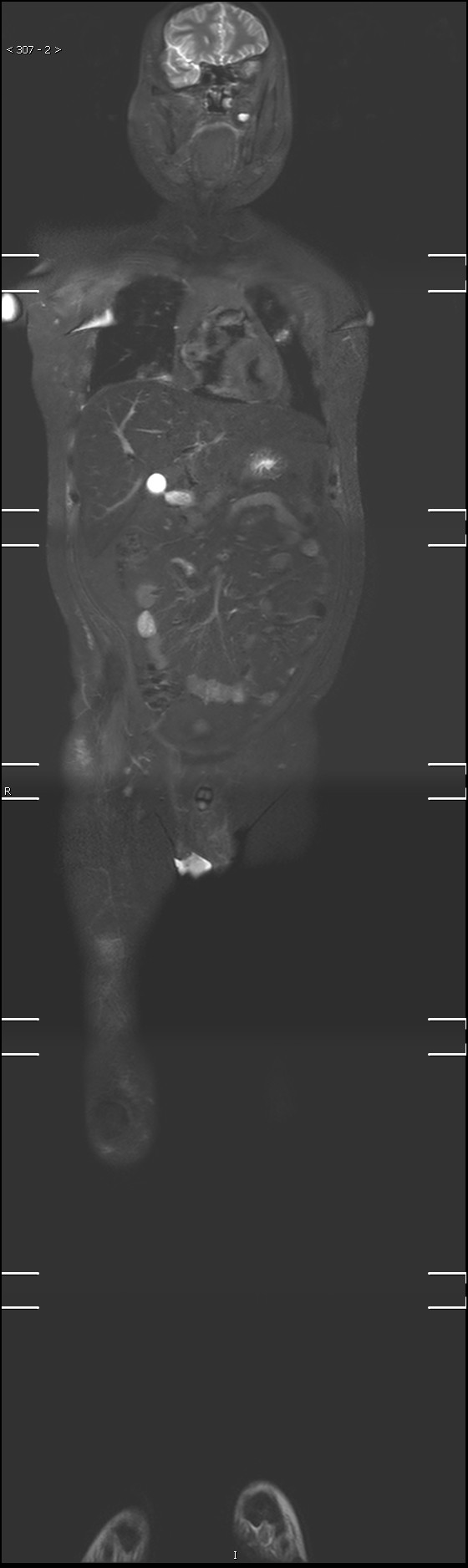

Supplement: S5 Fig — (ZIP) [file pone.0181069.s005.zip › S5/02.jpg]

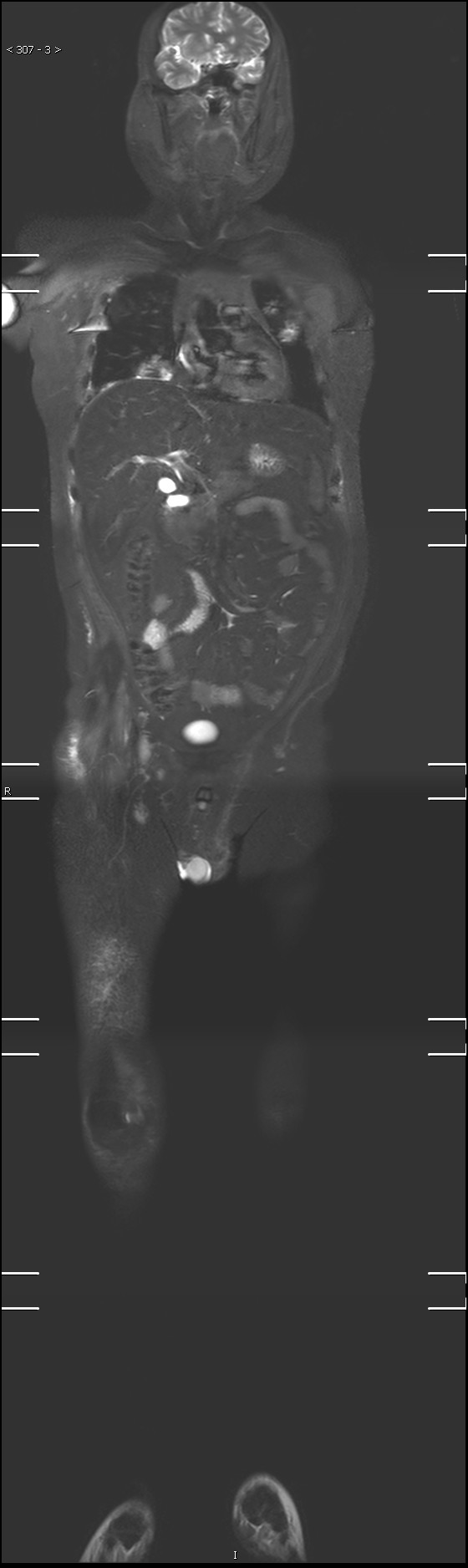

Supplement: S5 Fig — (ZIP) [file pone.0181069.s005.zip › S5/03.jpg]

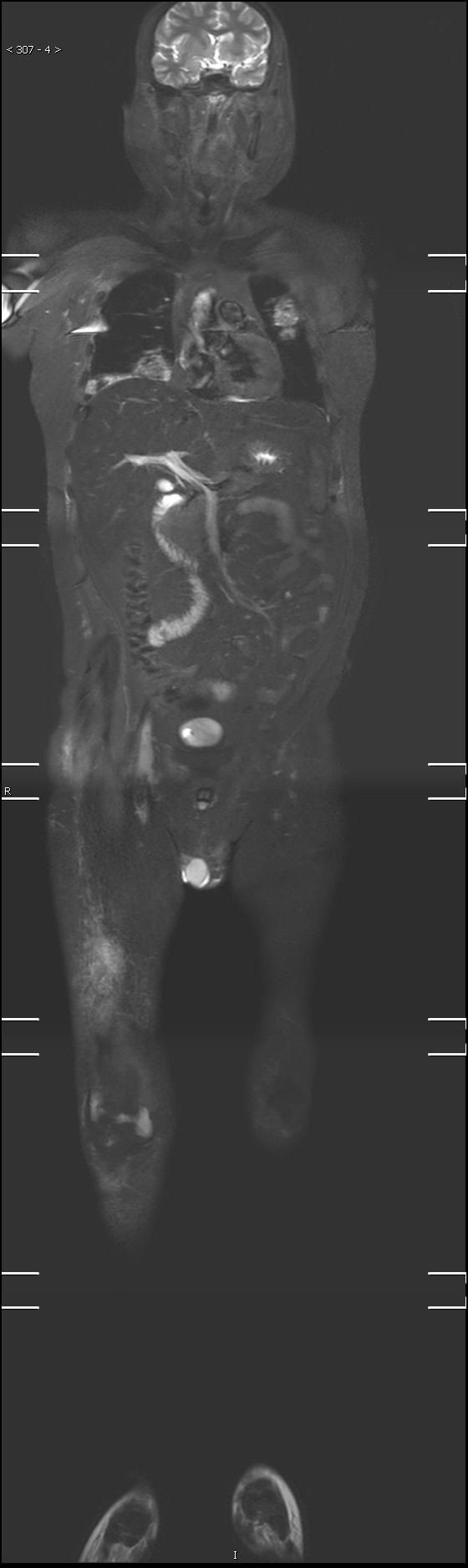

Supplement: S5 Fig — (ZIP) [file pone.0181069.s005.zip › S5/04.jpg]

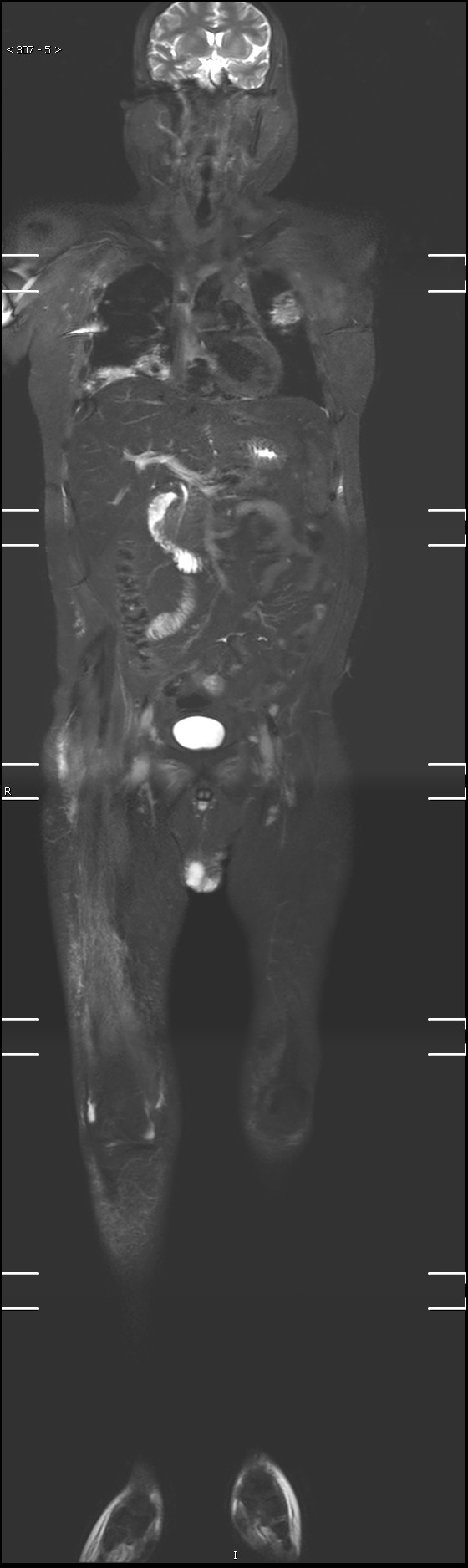

Supplement: S5 Fig — (ZIP) [file pone.0181069.s005.zip › S5/05.jpg]

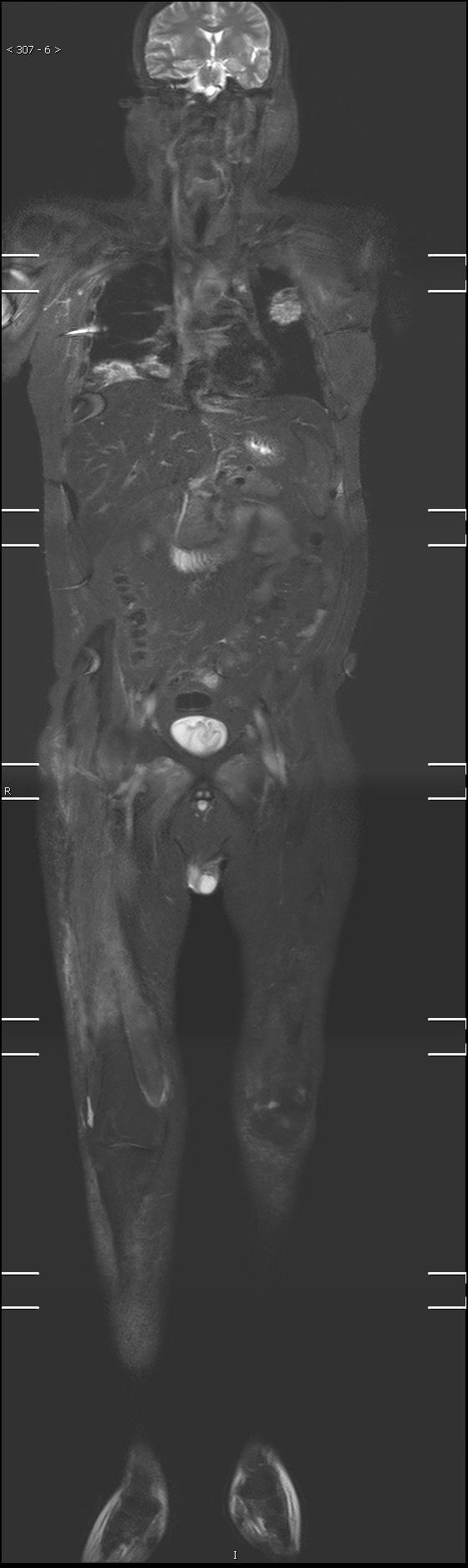

Supplement: S5 Fig — (ZIP) [file pone.0181069.s005.zip › S5/06.jpg]

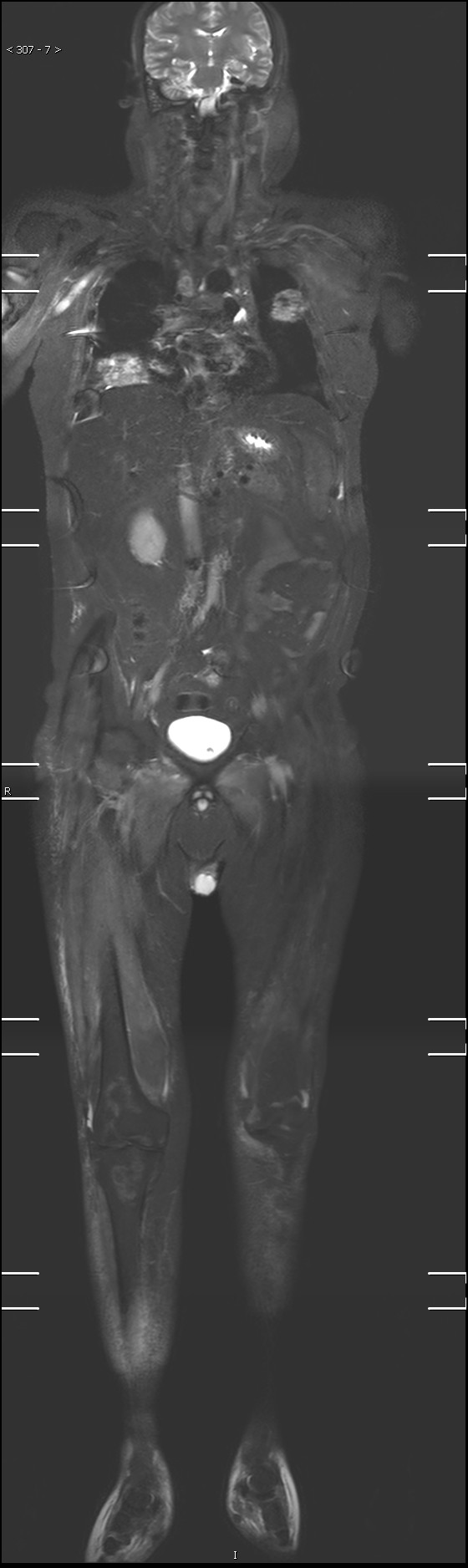

Supplement: S5 Fig — (ZIP) [file pone.0181069.s005.zip › S5/07.jpg]

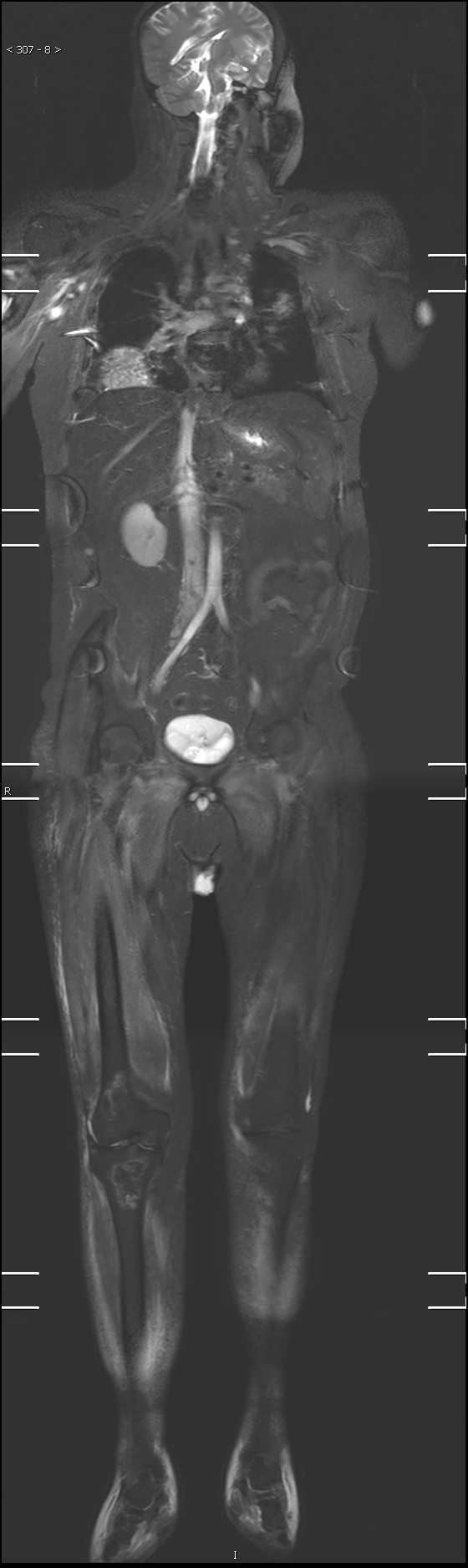

Supplement: S5 Fig — (ZIP) [file pone.0181069.s005.zip › S5/08.jpg]

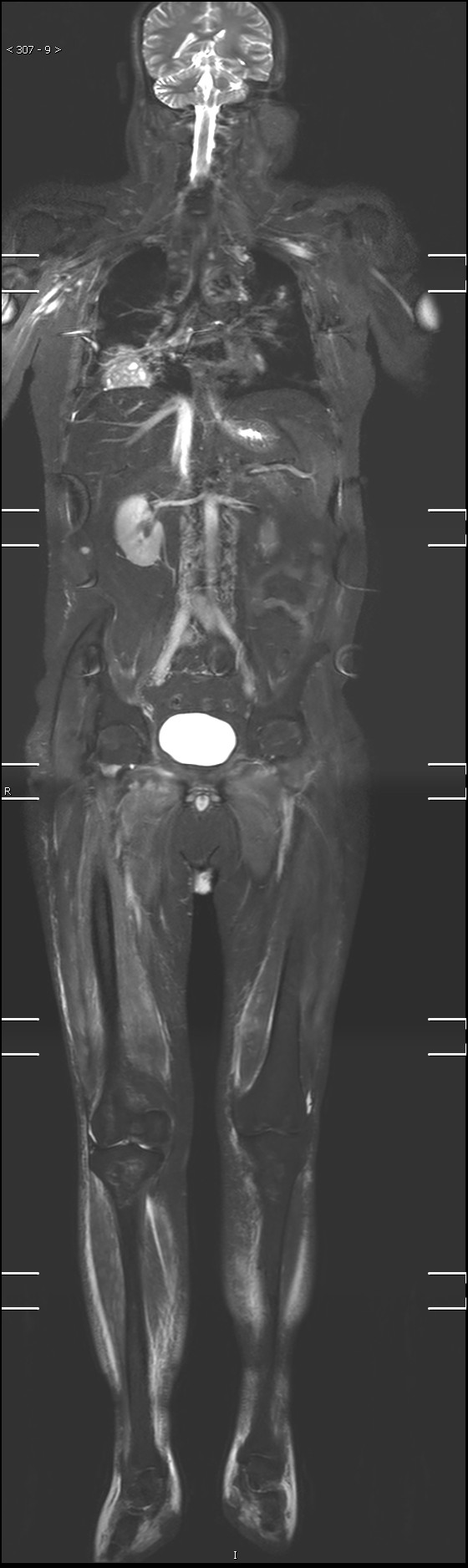

Supplement: S5 Fig — (ZIP) [file pone.0181069.s005.zip › S5/09.jpg]

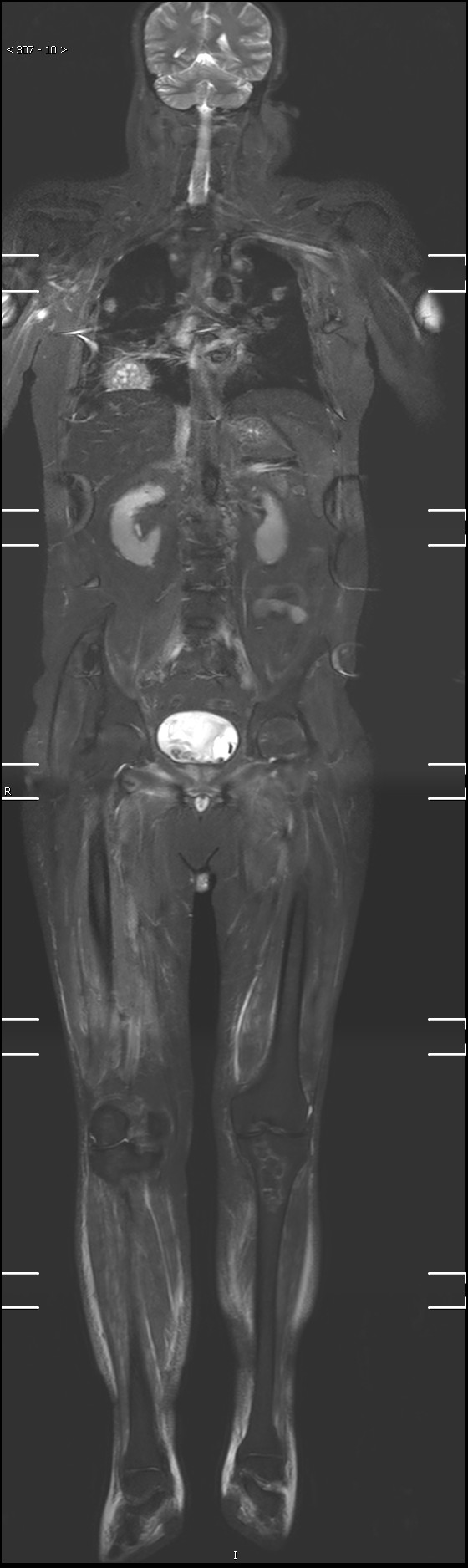

Supplement: S5 Fig — (ZIP) [file pone.0181069.s005.zip › S5/10.jpg]

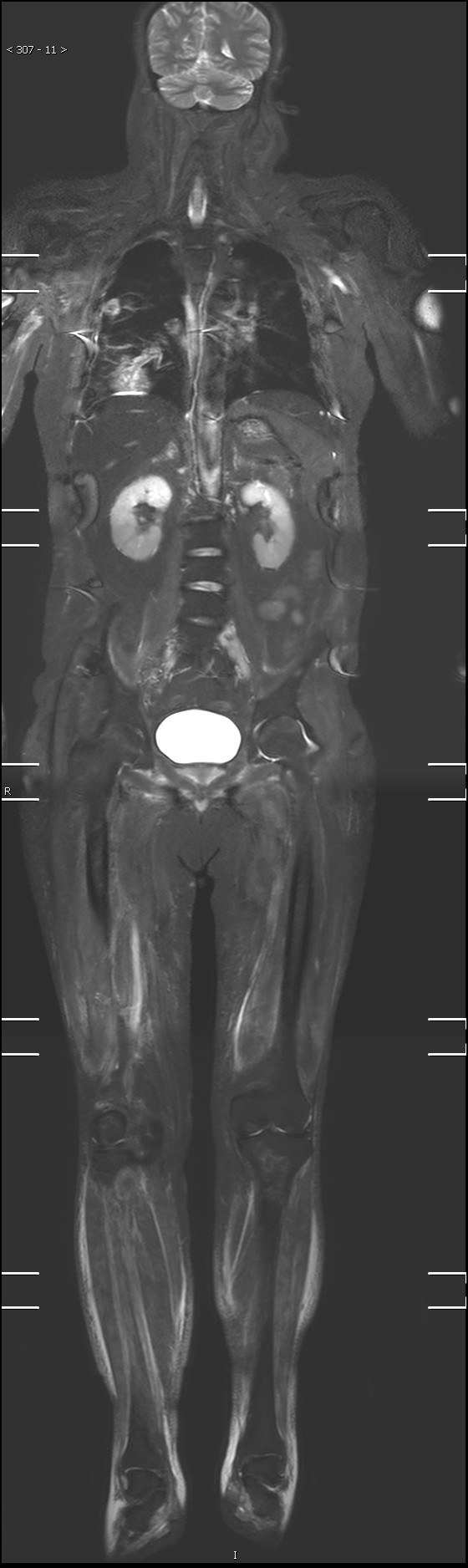

Supplement: S5 Fig — (ZIP) [file pone.0181069.s005.zip › S5/11.jpg]

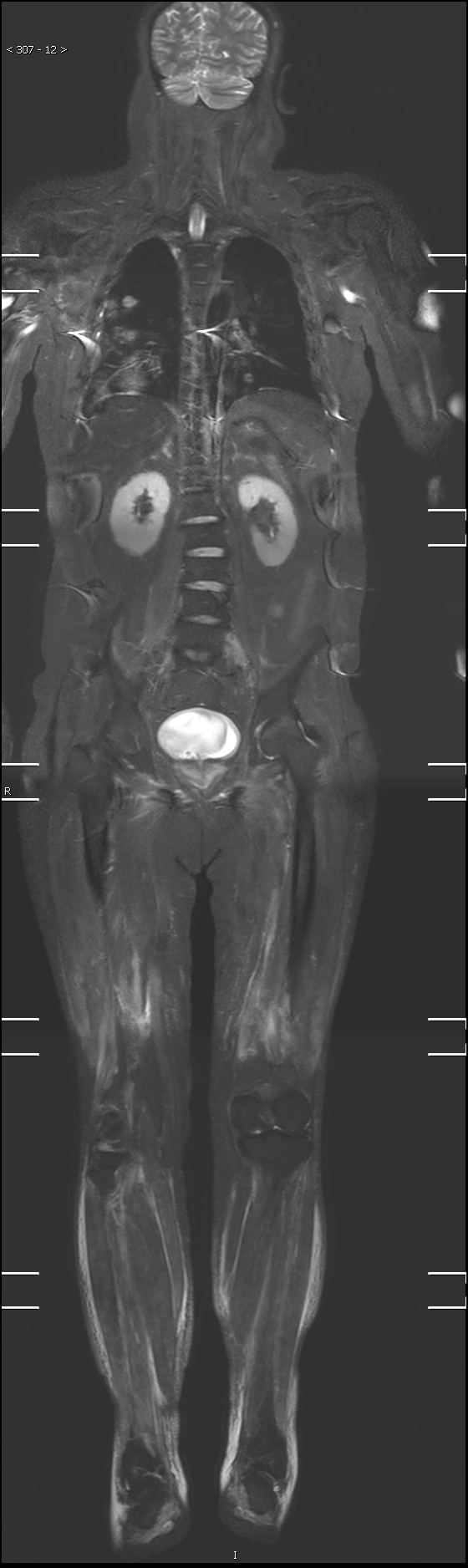

Supplement: S5 Fig — (ZIP) [file pone.0181069.s005.zip › S5/12.jpg]

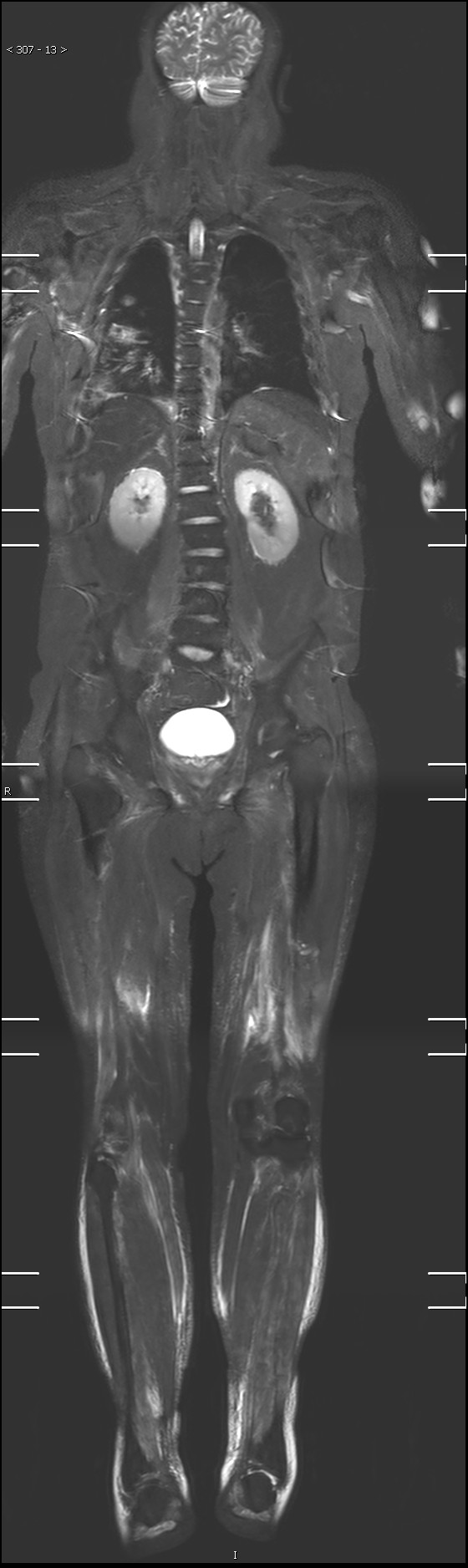

Supplement: S5 Fig — (ZIP) [file pone.0181069.s005.zip › S5/13.jpg]

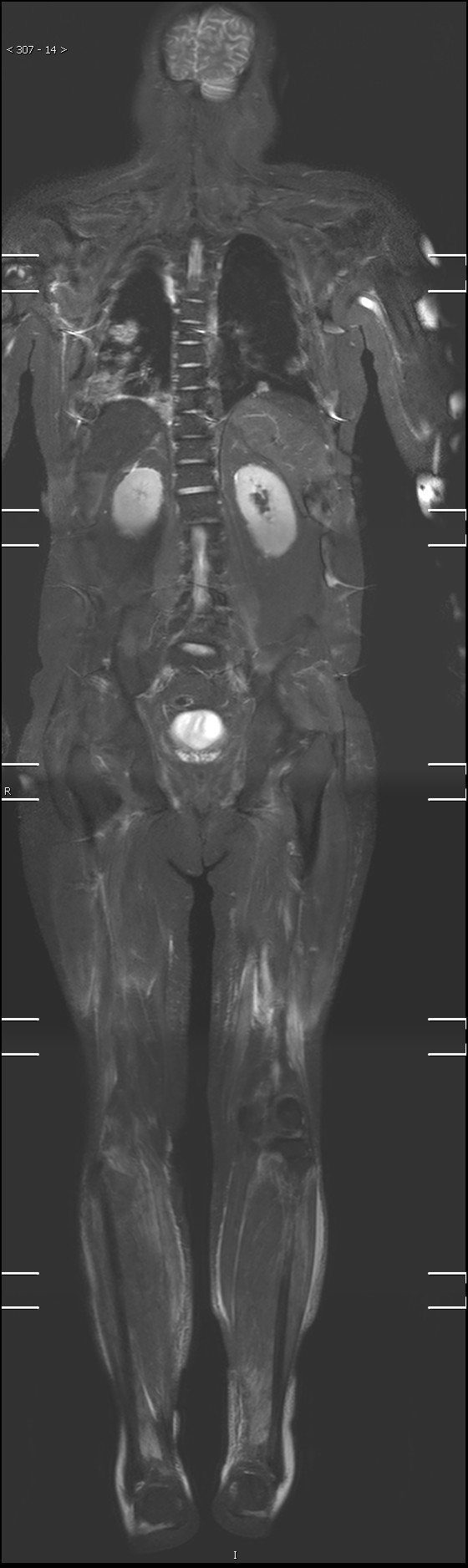

Supplement: S5 Fig — (ZIP) [file pone.0181069.s005.zip › S5/14.jpg]

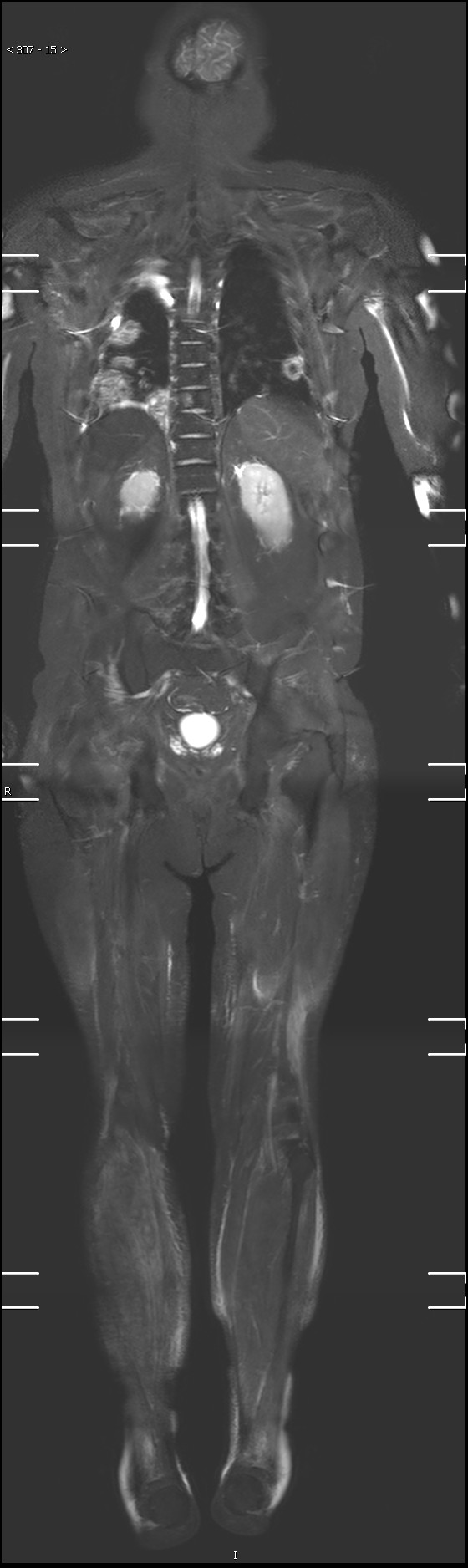

Supplement: S5 Fig — (ZIP) [file pone.0181069.s005.zip › S5/15.jpg]

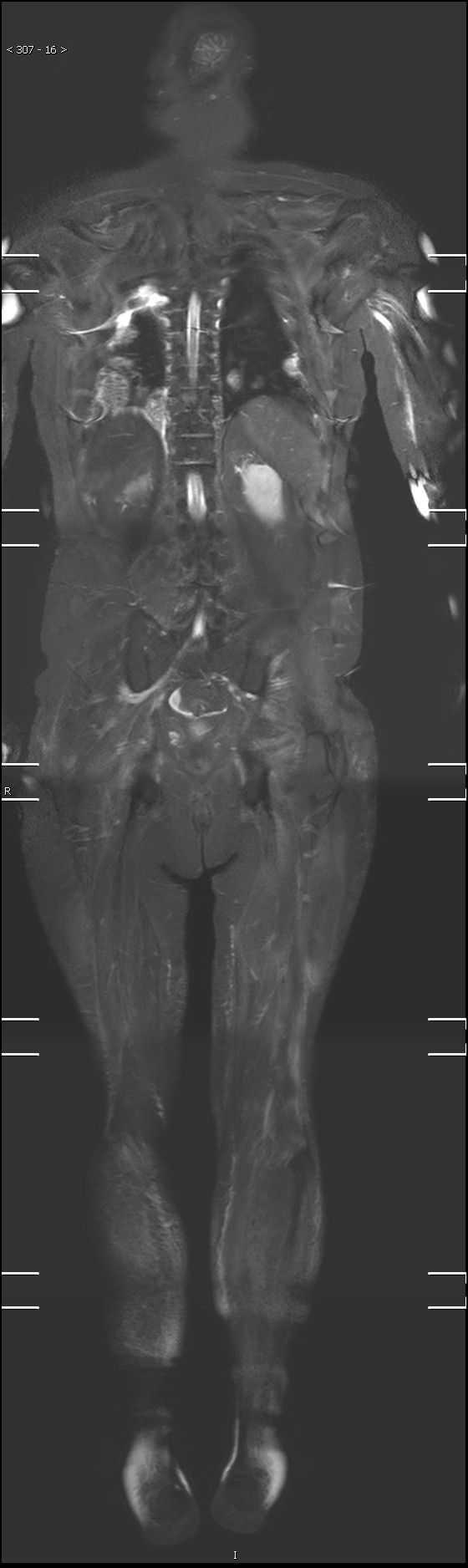

Supplement: S5 Fig — (ZIP) [file pone.0181069.s005.zip › S5/16.jpg]

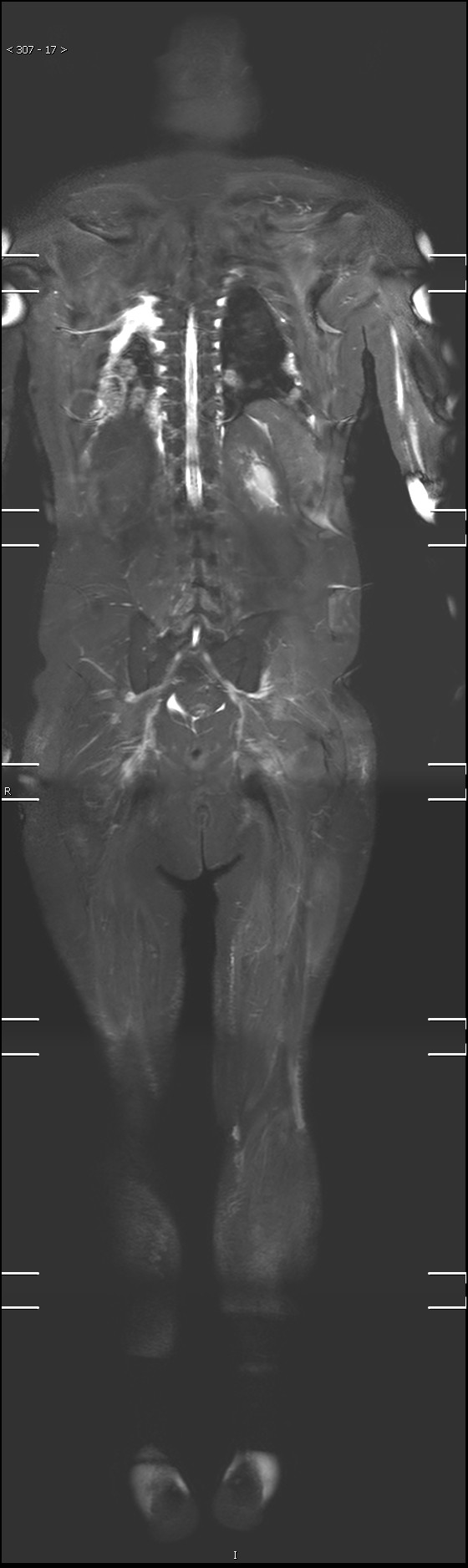

Supplement: S5 Fig — (ZIP) [file pone.0181069.s005.zip › S5/17.jpg]

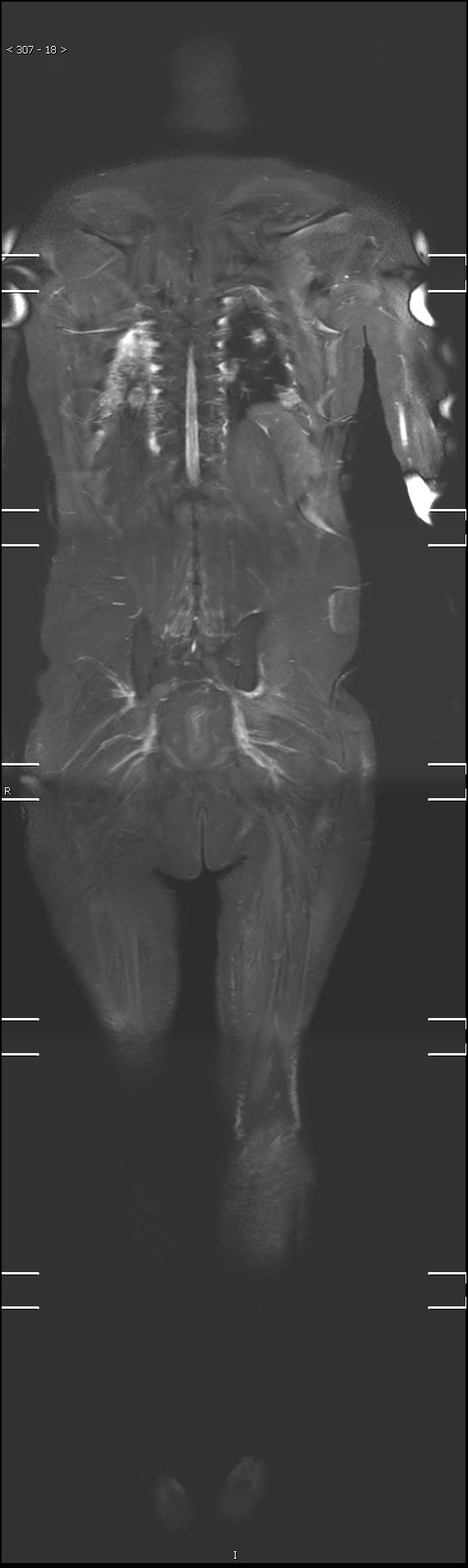

Supplement: S5 Fig — (ZIP) [file pone.0181069.s005.zip › S5/18.jpg]

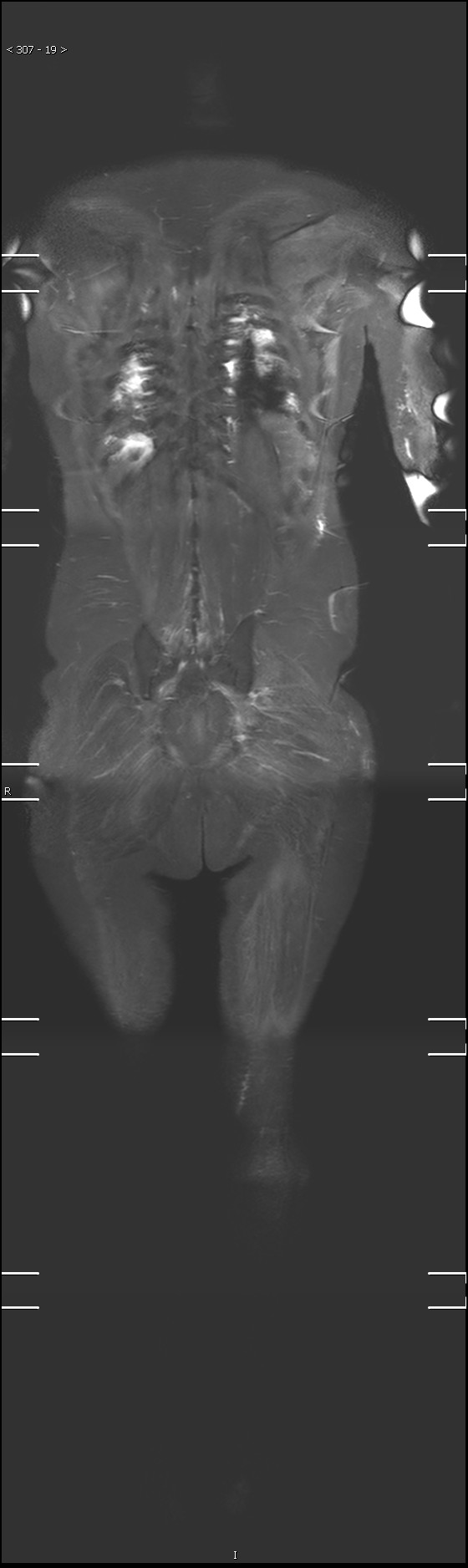

Supplement: S5 Fig — (ZIP) [file pone.0181069.s005.zip › S5/19.jpg]

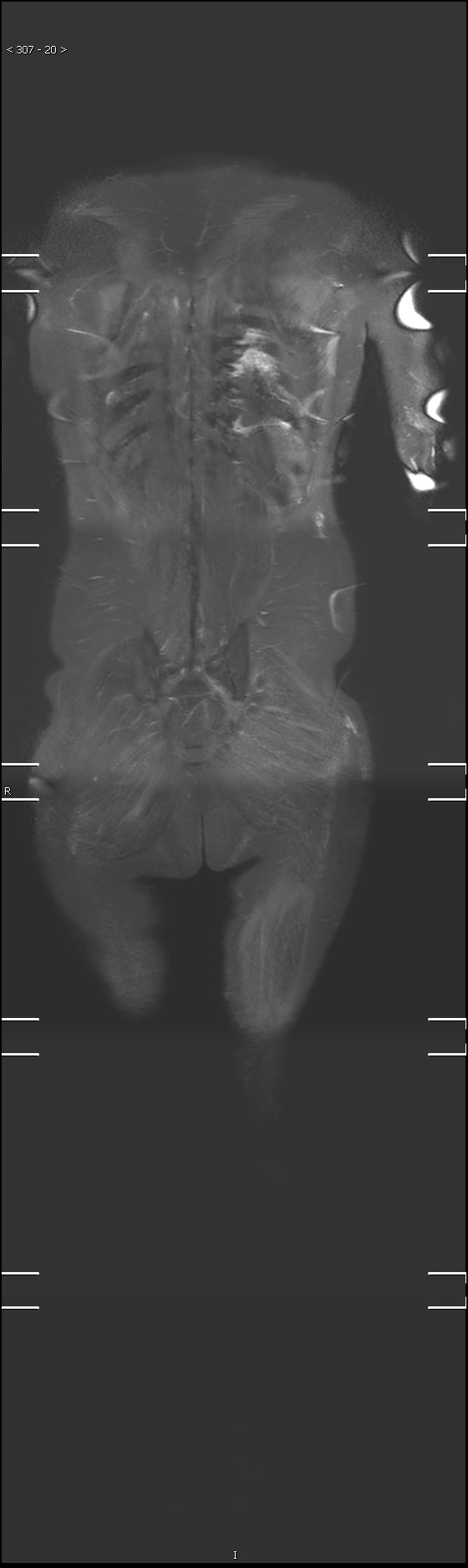

Supplement: S5 Fig — (ZIP) [file pone.0181069.s005.zip › S5/20.jpg]
