# Supplementary figures and images for: An efficacy analysis of whole-body magnetic resonance imaging in the diagnosis and follow-up of polymyositis and dermatomyositis (part 2 of 2)
Source: PLoS One. 2017 Jul 17;12(7):e0181069. doi: 10.1371/journal.pone.0181069 (PMC5513424; doi:10.1371/journal.pone.0181069)

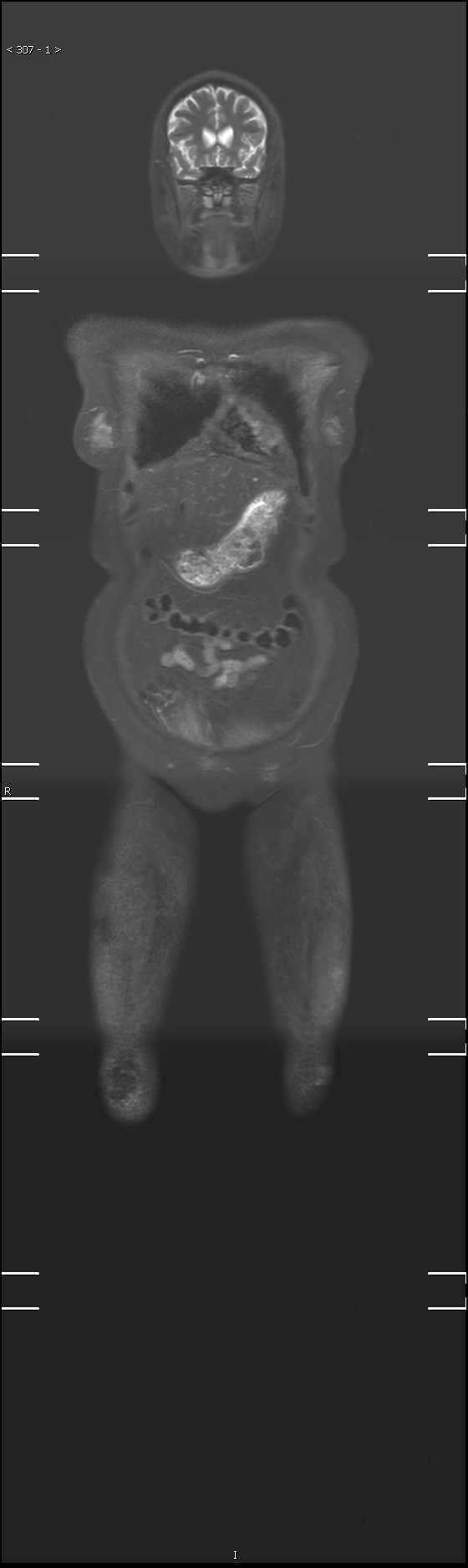

Supplement: S6 Fig — (ZIP) [file pone.0181069.s006.zip › S6/01.jpg]

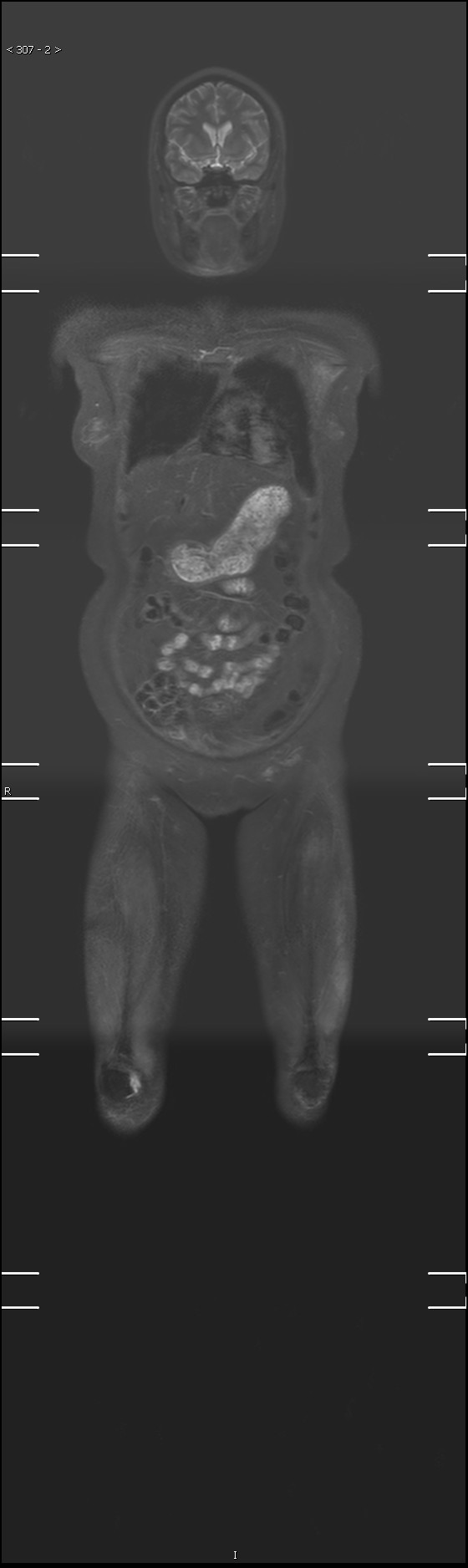

Supplement: S6 Fig — (ZIP) [file pone.0181069.s006.zip › S6/02.jpg]

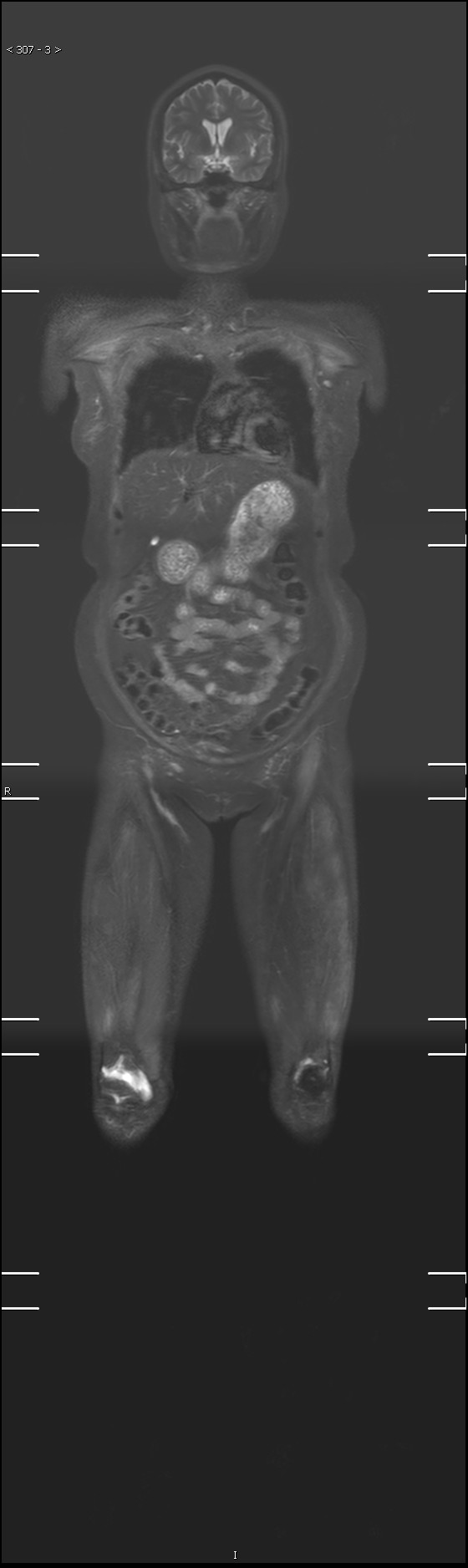

Supplement: S6 Fig — (ZIP) [file pone.0181069.s006.zip › S6/03.jpg]

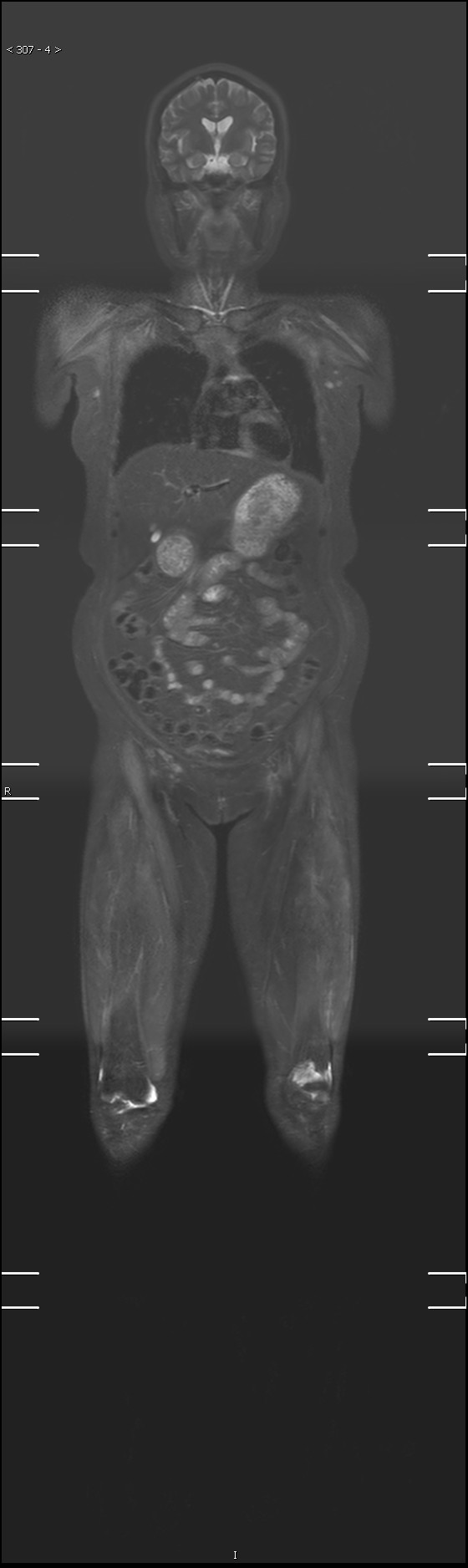

Supplement: S6 Fig — (ZIP) [file pone.0181069.s006.zip › S6/04.jpg]

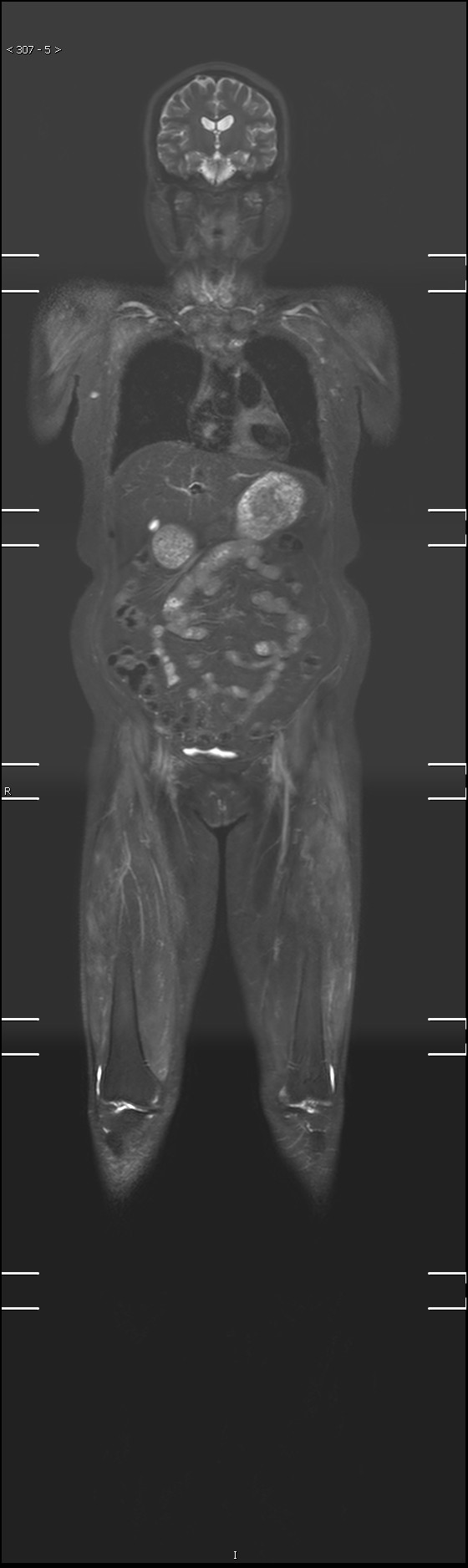

Supplement: S6 Fig — (ZIP) [file pone.0181069.s006.zip › S6/05.jpg]

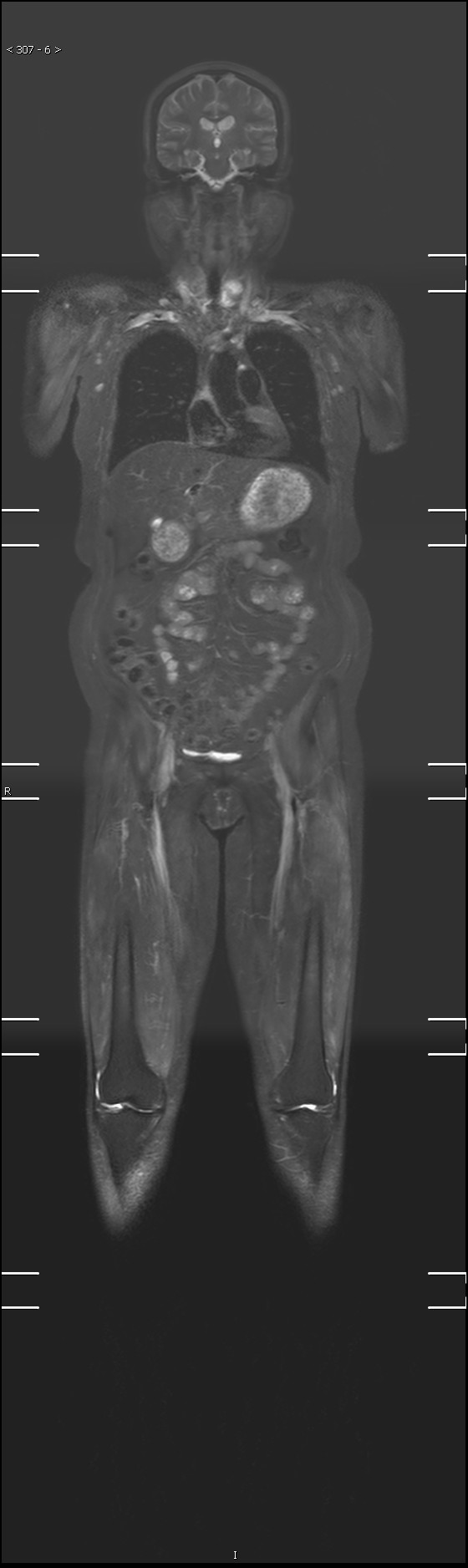

Supplement: S6 Fig — (ZIP) [file pone.0181069.s006.zip › S6/06.jpg]

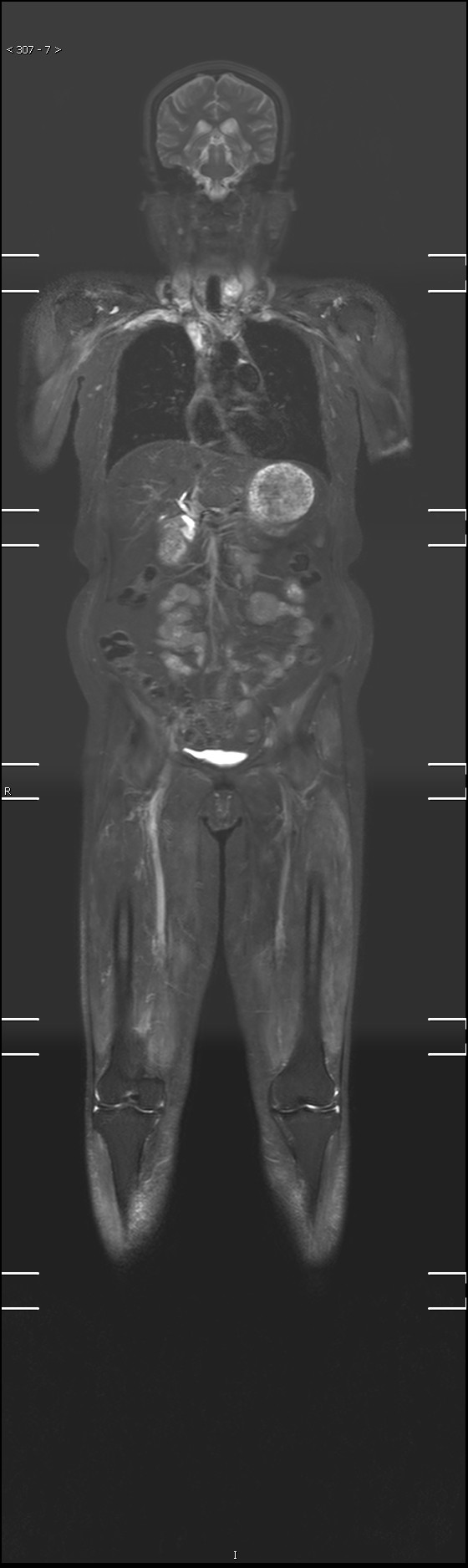

Supplement: S6 Fig — (ZIP) [file pone.0181069.s006.zip › S6/07.jpg]

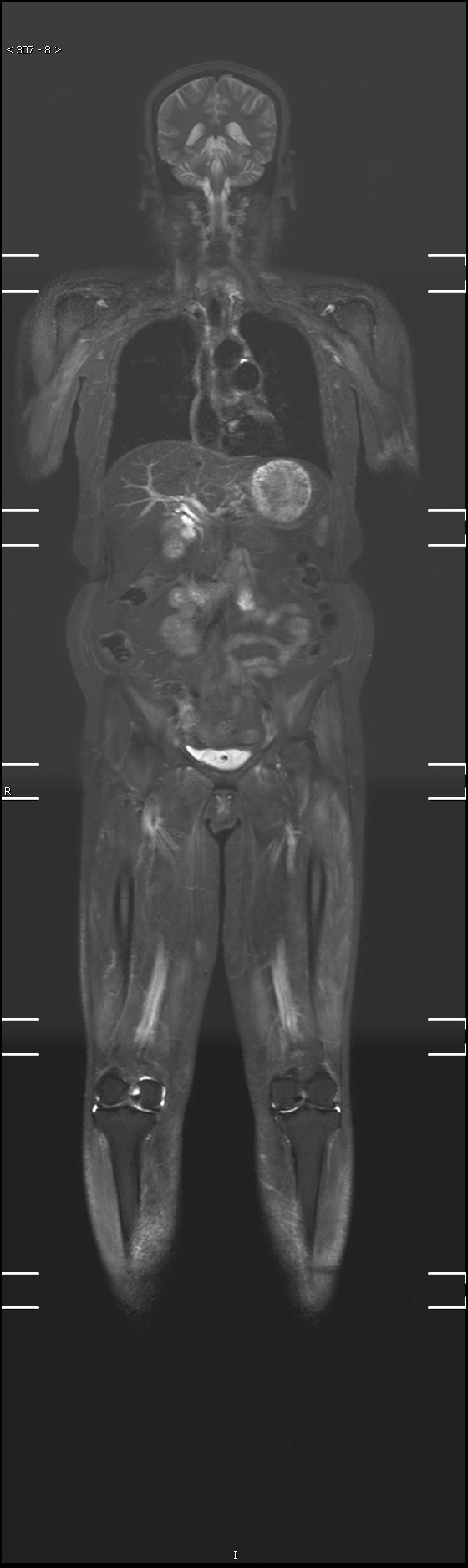

Supplement: S6 Fig — (ZIP) [file pone.0181069.s006.zip › S6/08.jpg]

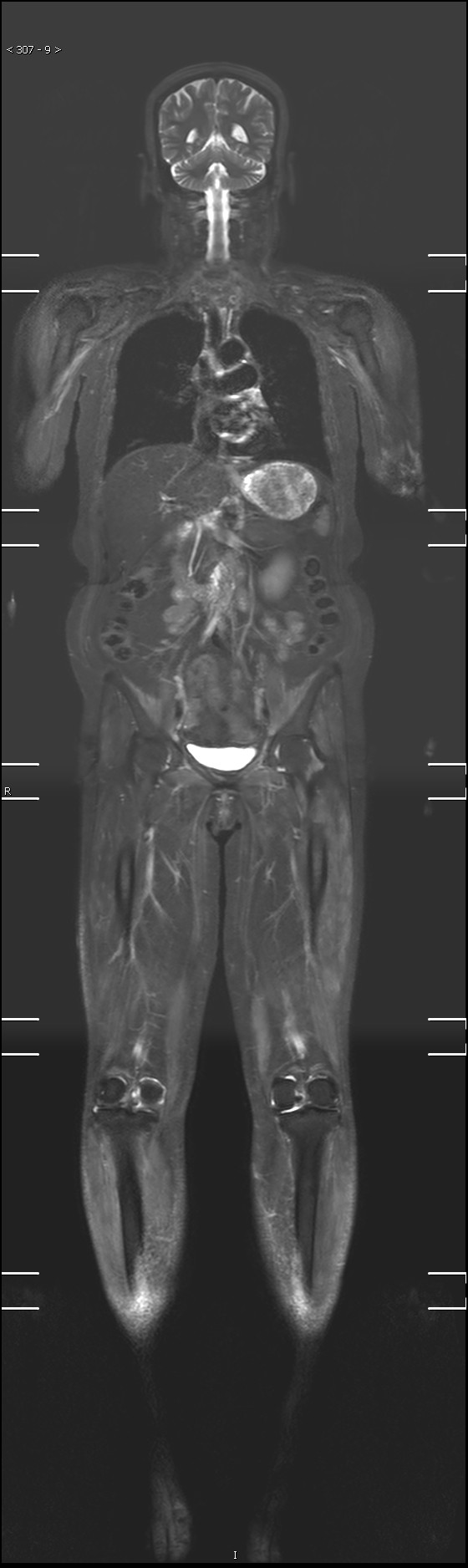

Supplement: S6 Fig — (ZIP) [file pone.0181069.s006.zip › S6/09.jpg]

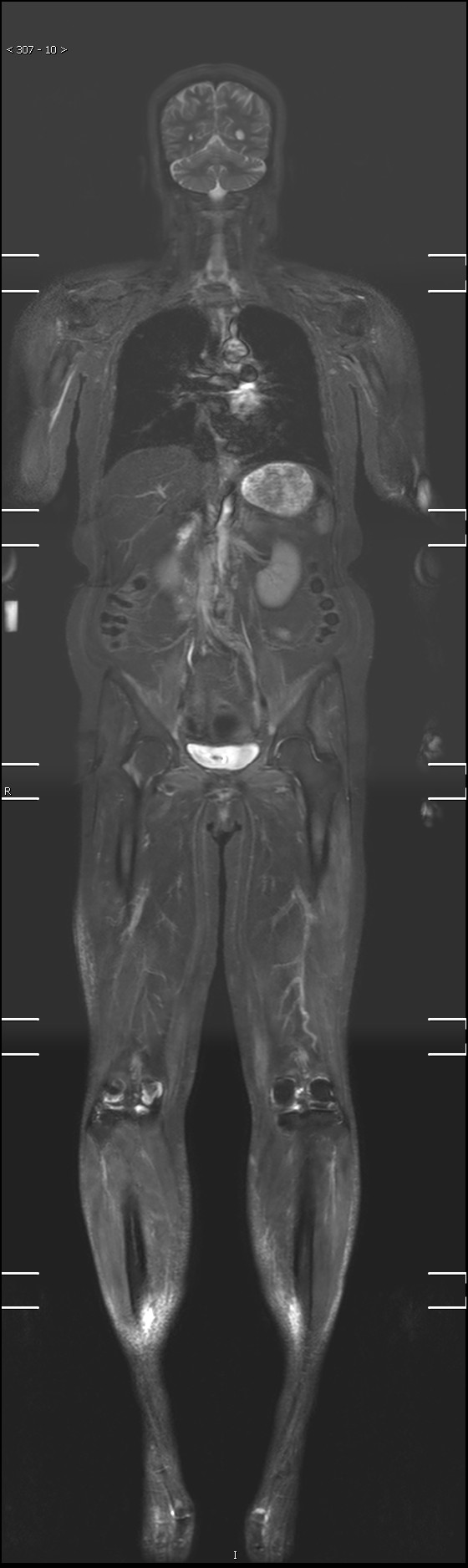

Supplement: S6 Fig — (ZIP) [file pone.0181069.s006.zip › S6/10.jpg]

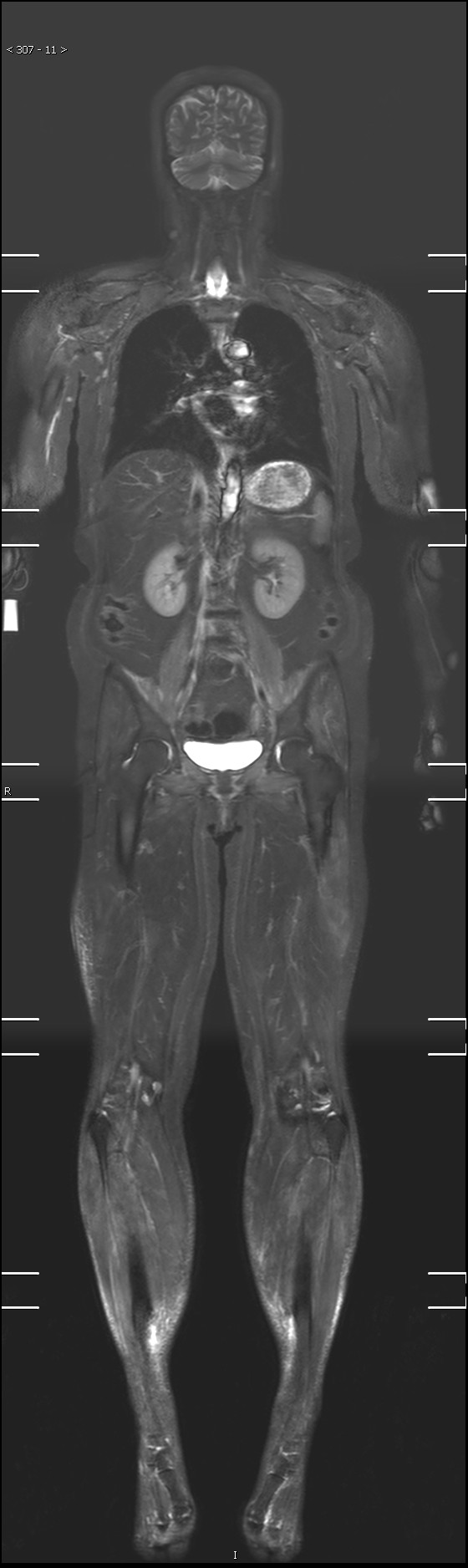

Supplement: S6 Fig — (ZIP) [file pone.0181069.s006.zip › S6/11.jpg]

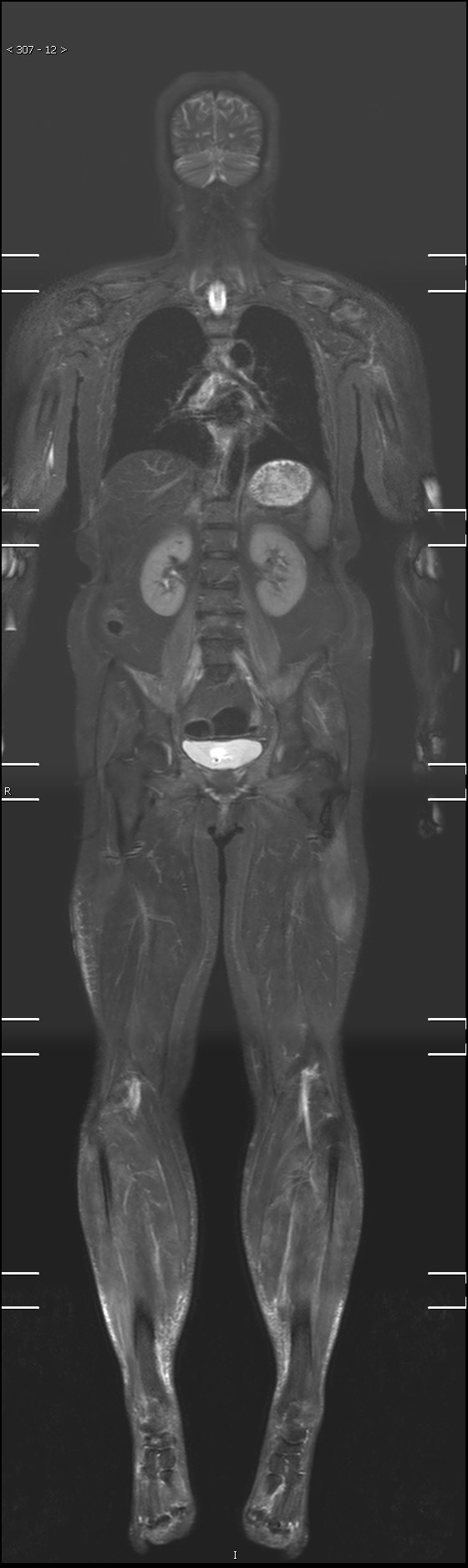

Supplement: S6 Fig — (ZIP) [file pone.0181069.s006.zip › S6/12.jpg]

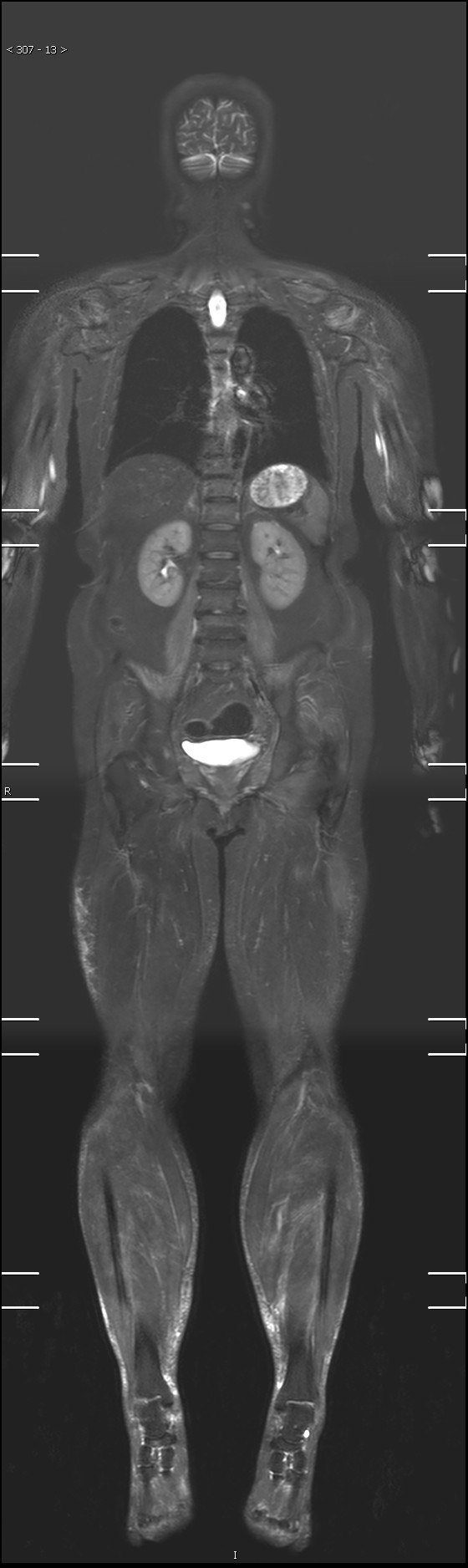

Supplement: S6 Fig — (ZIP) [file pone.0181069.s006.zip › S6/13.jpg]

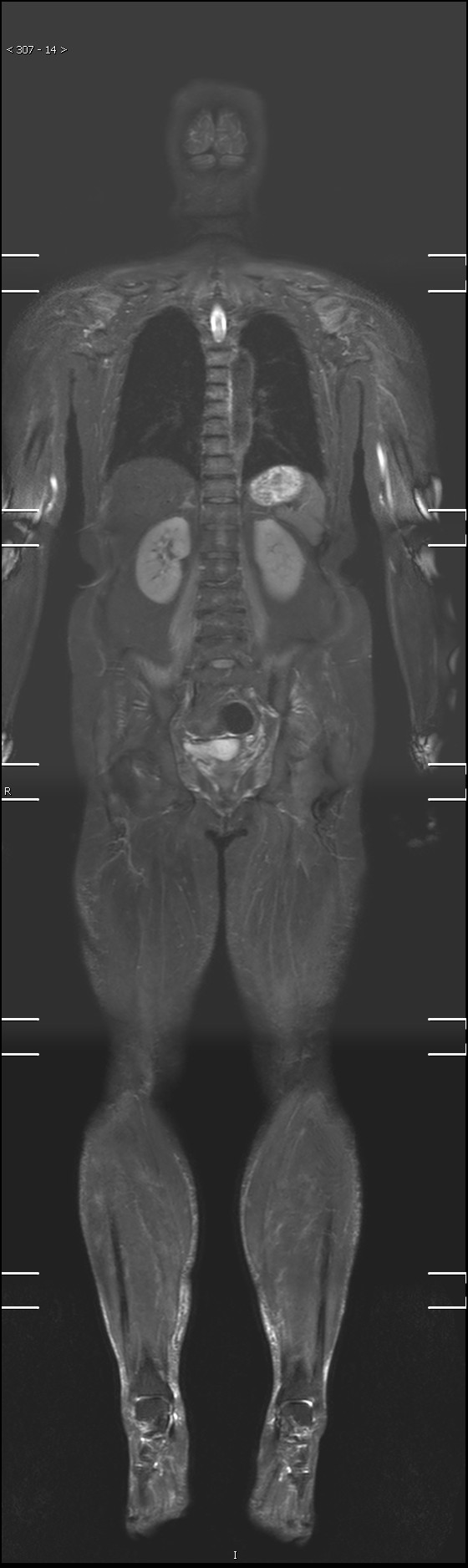

Supplement: S6 Fig — (ZIP) [file pone.0181069.s006.zip › S6/14.jpg]

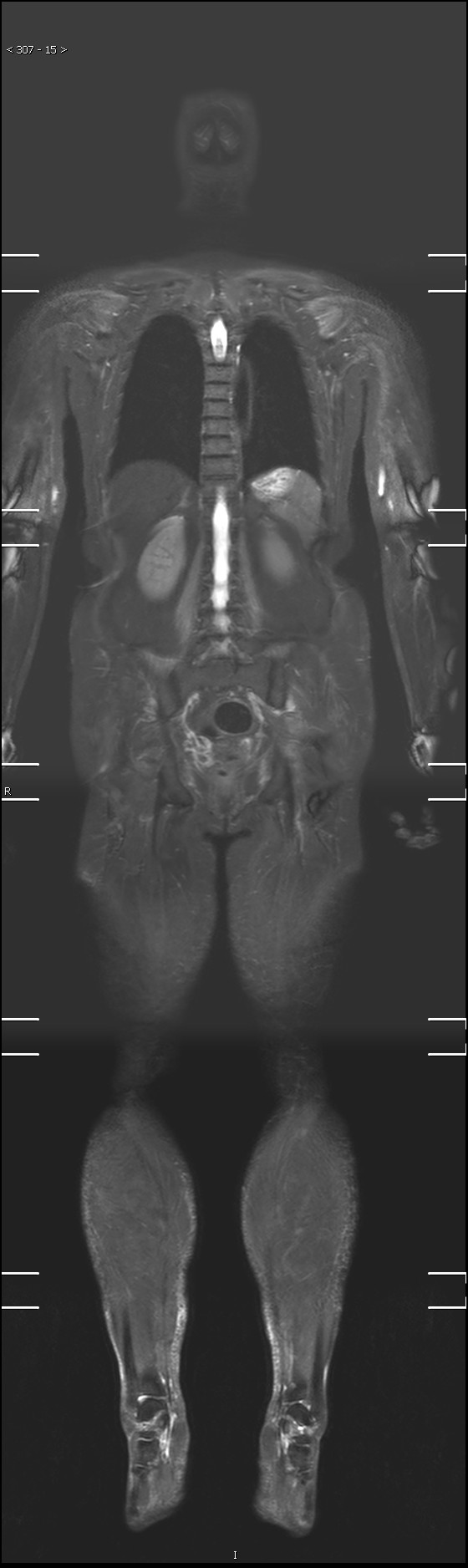

Supplement: S6 Fig — (ZIP) [file pone.0181069.s006.zip › S6/15.jpg]

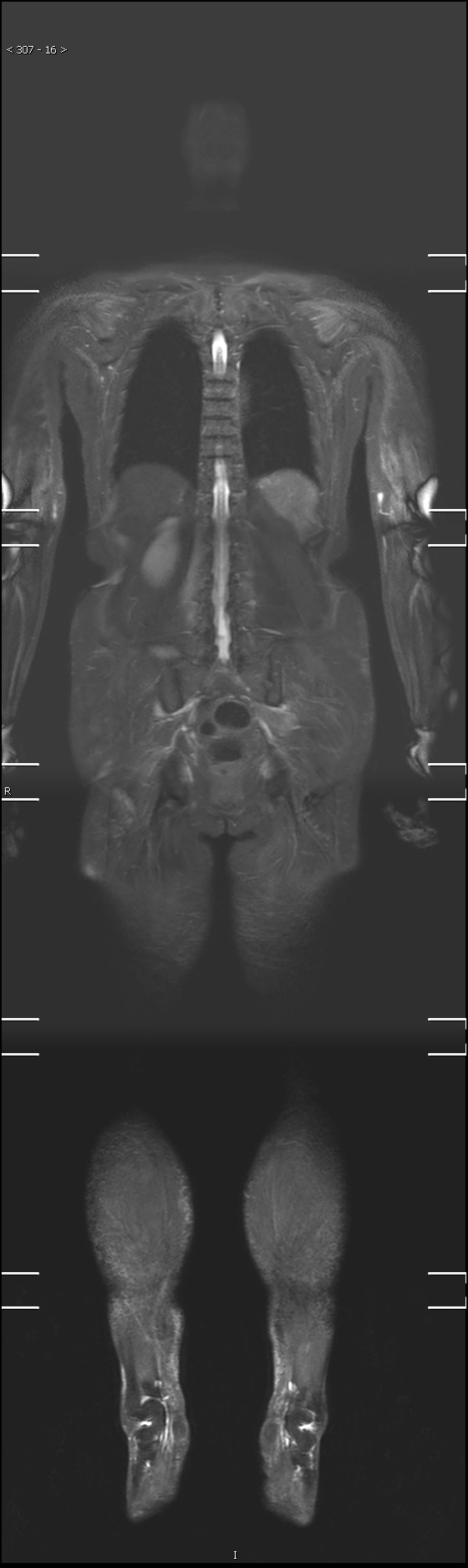

Supplement: S6 Fig — (ZIP) [file pone.0181069.s006.zip › S6/16.jpg]

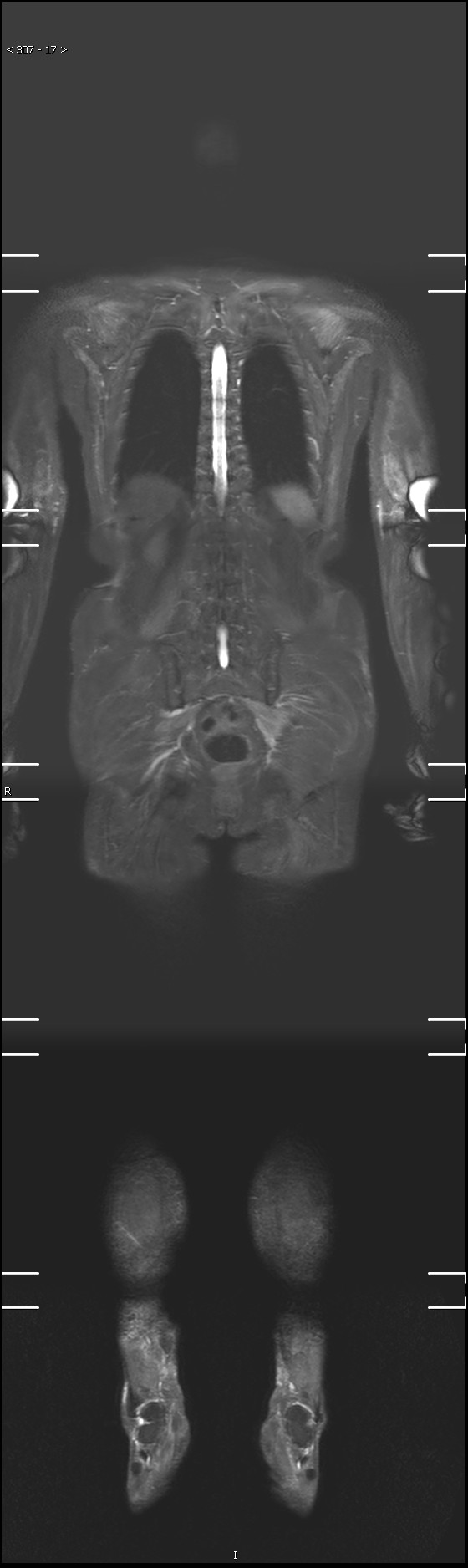

Supplement: S6 Fig — (ZIP) [file pone.0181069.s006.zip › S6/17.jpg]

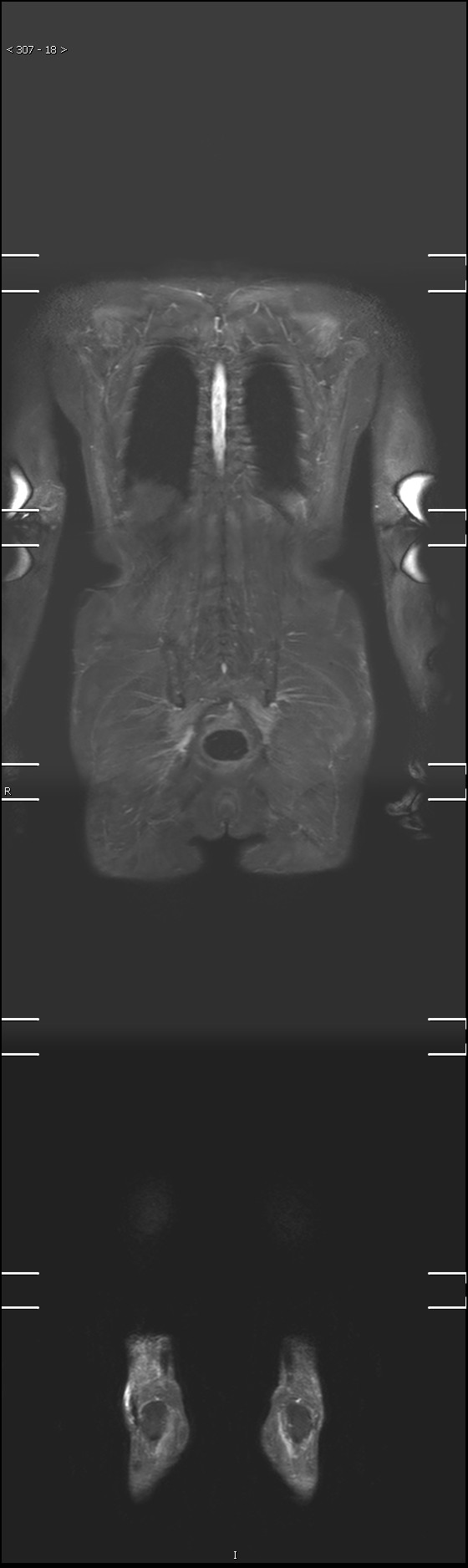

Supplement: S6 Fig — (ZIP) [file pone.0181069.s006.zip › S6/18.jpg]

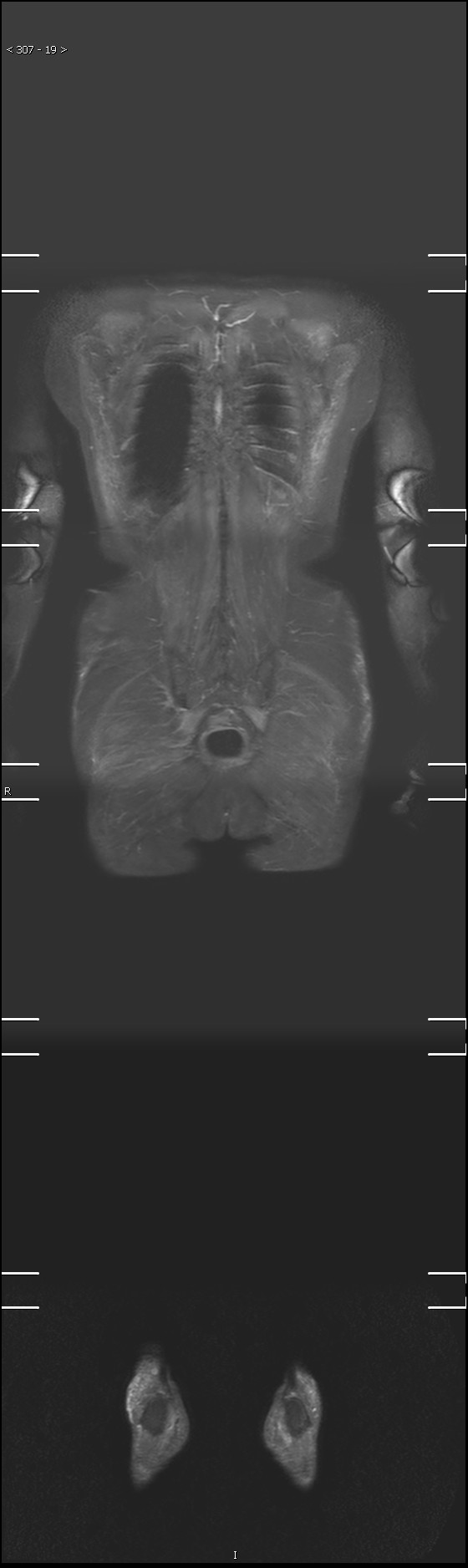

Supplement: S6 Fig — (ZIP) [file pone.0181069.s006.zip › S6/19.jpg]

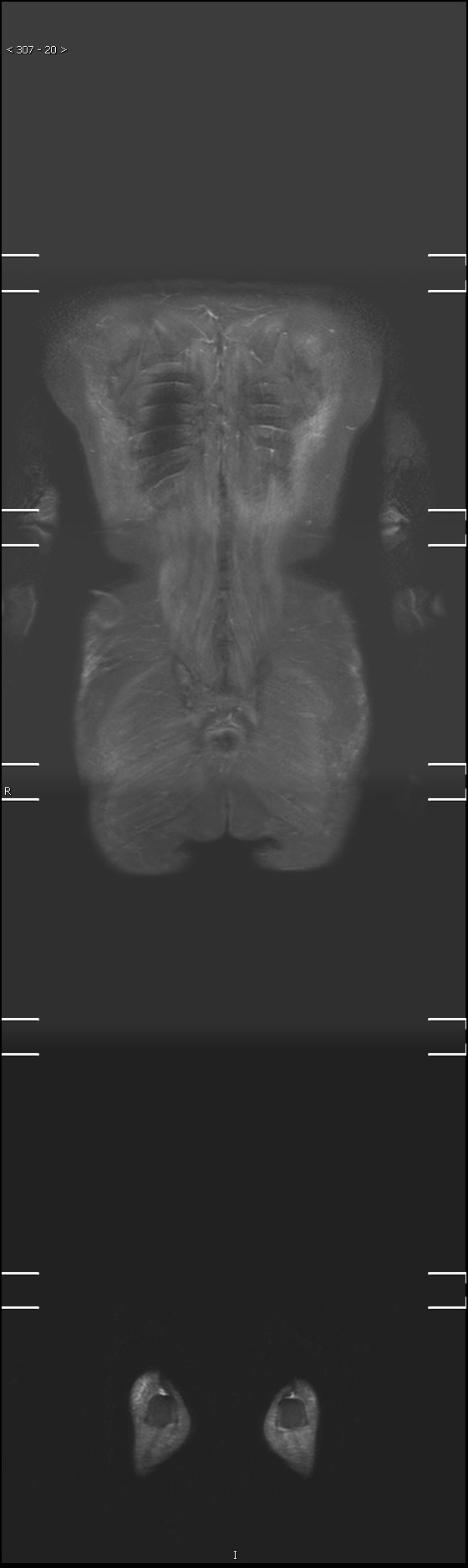

Supplement: S6 Fig — (ZIP) [file pone.0181069.s006.zip › S6/20.jpg]
